# Studies towards the Synthesis of Novel 3-Aminopropoxy-Substituted Dioxins Suitable for the Development of Aptamers for Photonic Biosensor Applications

**DOI:** 10.3390/ma14164727

**Published:** 2021-08-21

**Authors:** Stefania Kalantzi, Sofia Leonardi, Eleanna Vachlioti, Eleni G. Kaliatsi, Κοnstantina Papachristopoulou, Constantinos Stathopoulos, Nikolaos Vainos, Dionissios Papaioannou

**Affiliations:** 1 Laboratory of Synthetic Organic Chemistry, Department of Chemistry, University of Patras, GR-26504 Patras, Greece; s.kalantzi8@gmail.com (S.K.); ssofiaa30@gmail.com (S.L.); eleannavah@yahoo.gr (E.V.); 2 Department of Biochemistry, School of Medicine, University of Patras, GR-26504 Patras, Greece; lenakaliatsi@hotmail.gr (E.G.K.); cstath@upatras.gr (C.S.); 3 Photonics Nanotechnology Research Laboratory, Department of Materials Science, University of Patras, GR-26504 Patras, Greece; k.papachristopoulou@upnet.gr (K.P.); vainos@upatras.gr (N.V.)

**Keywords:** dioxins, tetrachlorodibenzodioxins, tetrachlorodibenzofurans, hydroxyl-substituted dioxins, amino-substituted dioxins, carboxy-substituted magnetic beads, immobilization

## Abstract

Hydroxy-substituted tetrachlorodibenzo[*b,e*][1,4]dioxin and tetrachlorodibenzo[*b,d*]furans have been synthesized using 3,4-dichloroanisole, 2,3,6-trichlorophenol and 4,5-dichlorocatechol as starting materials and electrophilic and/or nucleophilic aromatic substitution reactions for the assembly of the dibenzo[*b,e*][1,4]dioxin and dibenzo[*b,d*]furan systems. The thus-obtained phenolic compounds were then alkylated with N-1-(4,4-dimethyl-2,6-dioxocyclohexylidene)ethyl (Dde)-protected 3-bromopropan-1-amine to give the corresponding N-Dde protected 3-aminopropoxy-substituted tetrachlorodibenzo[*b,e*][1,4]dioxin and tetrachlorodibenzo[*b,d*]furans, respectively. Hydrazinolysis-mediated Dde removal from the former compound provided the corresponding amino-substituted dioxin, which was coupled to carboxy-substituted magnetic beads affording magnetic beads coated by the amino-substituted dioxin. The latter is an attractive intermediate for the development of selective single-standard DNA (ssDNA) aptamers, which constitute molecular recognition elements in photonic biosensors with potential application to the monitoring of the dangerous environmental pollutants, dioxins having serious implications in human health.

## 1. Introduction

**Figure 1:**
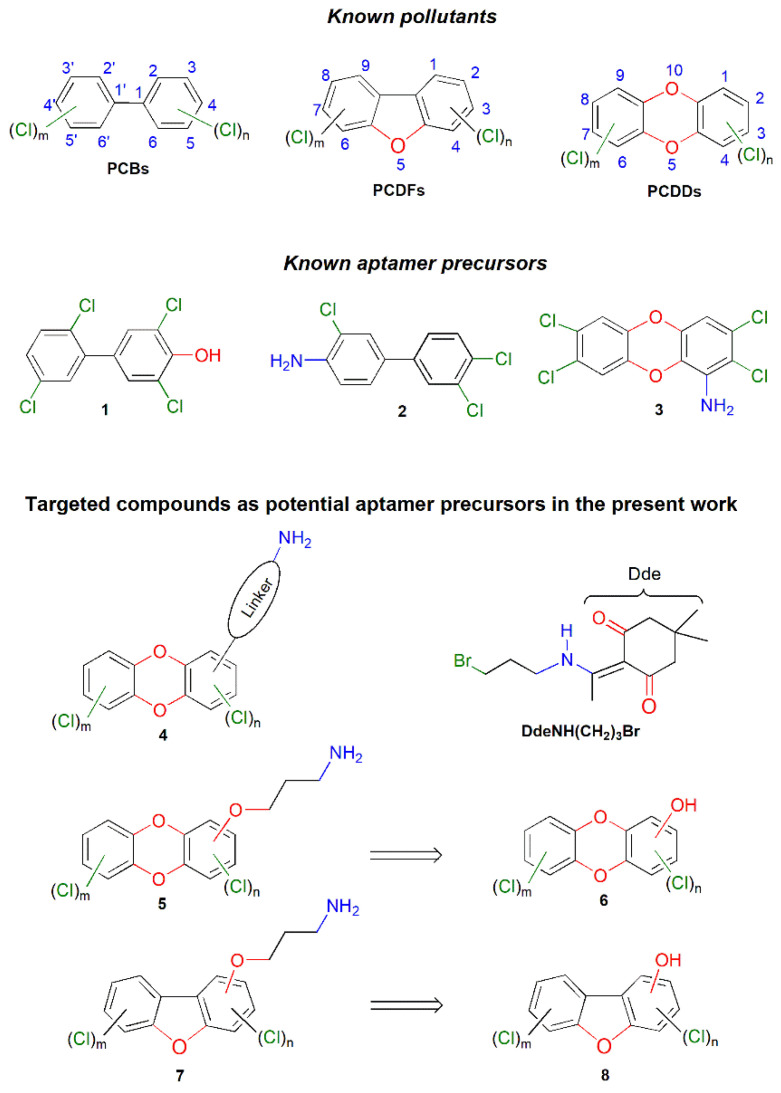
Structures of dioxins and related compounds referred to in this work.

Photonic biosensors are simple, portable, robust, and cost-effective analytical devices for the rapid and specific screening and sensitive monitoring of environmental pollutants, water, foods and beverages contaminants. Photonic biosensors combine molecular recognition elements (MREs) with optical transducer systems, which convert light rays into electronic signals, so that the specific binding of a target molecule to the MRE to be finally translated into a measurable electrical signal [1,2,3]. Aptamers are among the quite frequently used MREs in such devices for the quantification of environmental pollutants. Aptamers are short, target specific, single-stranded synthetic oligonucleotides (ssDNA, RNA) or peptide sequences, which fold into three-dimensional shapes capable of binding non-covalently and with high affinity to a target molecule. They are readily generated through an in vitro process called Systematic Evolution of Ligands by EXponential Enrichment (SELEX). A core classical SELEX technique for aptamer selection is the “Magnetic bead-based SELEX” [4], in which the target molecule is covalently attached on, e.g., carboxylic acid functionalized magnetic beads through an amide bond [5,6,7,8,9,10]. Although aptamers for a variety of environmental pollutants with serious implications in human health, including the dioxin-like polychlorinated biphenyls (PCBs), have been already developed, aptamers for polychlorinated dibenzofurans (PCDFs) and dibenzo-*p*-dioxins (PCDDs), a group of small organic molecules collectively known as “dioxins”, schematically shown in Figure 1, are missing [11,12]. In particular, aptamers for PCBs have been either obtained through anchoring hydroxyl-substituted PCBs, such as compound **1**, on carboxy-substituted magnetic beads through an ester bond [13], or amino-substituted PCBs, such as **2**, on an epoxy-activated agarose matrix through a N-C bond [14]. On the other hand, attempted anchoring of an amino-substituted PCDD, such as **3** (Figure 1) on a Sepharose resin was problematic due to the low nucleophilicity of the aromatic amino function [12].

Accordingly, we decided initially to study the feasibility of synthesizing amino-substituted PCDDs of the general type **4** (Figure 1) from commercially available PCDDs and using reactions, e.g., Heck reaction or formylation followed by Wittig reaction, we have previously successfully applied in the psoralen nucleus [15]. In compounds **4**, the required amino functionality was designed to be attached on the dioxin nucleus through a suitable hydrocarbon linker. However, due to the high toxicity, the low availability and cost problems associated with the appropriate PCDDs (also PCDFs) as starting materials, we had to change our strategy and attempt the total synthesis of amino-substituted tetrachlorodibenzodioxins of the general type **5** using readily available and cost-affordable starting materials, such as chlorinated anisoles or phenols and catechols. In compounds **5**, the required amino group is connected to the dioxin nucleus through a propoxy-type linker which could be readily formed from the corresponding hydroxy-substituted PCDDs **6**, through *O*-alkylation with a suitable bromide, for example the 3-bromo-*N*-1-(4,4-dimethyl-2,6-dioxocyclohexylidene)ethylpropan-1-amine [DdeNHCH_2_)_3_Br], which has been recently used by our research group to alkylate the *o*-nitrophenylsulfonylated primary amino function of polyamines [16]. Notably, the 1-(4,4-dimethyl-2,6-dioxocyclohexylidene)ethyl (Dde) group has been used to protect selectively primary amino functions and is stable to acids and bases but is readily removed by a 2% solution of hydrazine in *N*,*N*-dimethylformamide (DMF) at ambient temperature.

Similar methodology could be furthermore designed for the synthesis of amino-substituted tetrachlorodibenzofurans of the general type **7**, which could arise from the corresponding hydroxy-substituted PCDFs, **8**.

## 2. Materials and Methods

### 2.1. General Information

Melting points were determined with an Electrothermal apparatus (Electrothermal, Essex, UK) and are uncorrected. Nuclear Magnetic Resonance (NMR) spectra were recorded on Bruker Avance III HD instrument (Bruker Daltonics GmbH & Co. KG, Bremen, Germany) operating at 600 MHz and 150 MHz for ^1^H and ^13^C nuclei, respectively. Chemical shifts (*δ*) are reported for CDCl_3_ solutions in parts per million (ppm) downfield from tetramethylsilane (TMS), used as internal standard; all homocoupling patterns (^n^*J*_H,H_ values) are given in Hertz (Hz). Electron-spray ionization (ESI) mass spectra were recorded at 30 eV, on a Waters Micromass ZQ spectrometer (Waters Corporation, Milford, MA, USA) using High Performance Liquid Chromatography (HPLC) grade MeOH as solvent. Ultraviolet (UV) spectra were recorded with a Thermo Scientific^TM^ Multiscan Sky Microplate spectrophotometer (Thermo Fischer Scientific, Waltham, MA, USA) and Infrared (IR) spectra were obtained on a Shimadzu IRTracer-100 ATR-FTIR spectrometer (Shimadzu, Kyoto, Japan). Elemental analyses for new compounds were determined on a Carlo Erba EA 1108 CHNS elemental analyzer (Carlo Erba, MI, Italy).

Flash column chromatography (FCC) was performed on Acros Organics silica gel 0.035–0.070 mm, 60 Å and Thin Layer Chromatography (TLC) on Merck silica gel 60 F_254_ films (0.2 mm) precoated on aluminium foil. Spots were visualized with UV light at 254 nm and by spraying with a ninhydrine solution (0.3 g ninhydrin, 3 mL gl. acetic acid, 97 mL 1-butanol), only applicable to final product **17**. The eluents or mixtures thereof (*v/v*) used for the development of TLC or FCC were: (A) Hexane/Toluene (9:1), (B) Hexane/Toluene (8:2), (C) Hexane/Toluene (7:3), (D) Hexane/Toluene (6:4), (E) Hexane/Toluene (1:1), (F) Hexane/Toluene (1:9), (G) 100% Toluene, (H) Hexane/Ethyl acetate (19:1), (I) Hexane/Ethyl acetate (8:2), (J) Hexane/Ethyl acetate (1:1), (K) Hexane/Dichloromethane (1:1), (L) Toluene/Ethyl acetate (95:5), (M) Toluene/Ethyl acetate (6:4), (N) Toluene/Ethyl acetate (3:7), (O) 100% Ethyl acetate, (P) 100% Chloroform, (Q) Chloroform/Methanol/conc. NH_3_ (9:1:0.1).

All solvents were dried and/or purified according to standard procedures prior to use. Solvents were routinely removed at ca. 40 °C under reduced pressure on Bücki Rotavapor RE 111. Air-sensitive reagents were handled under inert atmosphere (Ar). The starting materials for the syntheses, that is 3,4-dichloroanisole, 2,3,6-trichlorophenol, 4,5-dichlorocatechol and 2,3,5,6-tetrachloroanisole were purchased from Alfa Aesar (Haverhill, MA, USA), TCI (Tokyo, Japan), TRC (Tokyo, Japan) and CHEMSERVICE (West Chester, PA, USA), respectively. All reagents employed in the present work were purchased from either Alfa Aesar or Acros Organics (Ferrand, NJ, USA) and were used without further purification. Magnetic beads used for the immobilization of dioxin **17** were Merck Millipore PureProteome^TM^ 1.0 μM Carboxy FlexiBend Magnetic Bead System with a capacity of 97–114 μmole carboxylic acid/g beads. Compound **20** is commercially available but for the needs of the present work it was prepared from phenol **S2** (Supplementary Materials). Compounds **S1** and **19** are described in ref. [17] but herein they were prepared differently as described in the Supplementary Materials. Compounds **S3**–**S6** and **21–22** were synthesized (Supplementary Materials) for the needs of this work according to ref. [17].

### 2.2. 3,4-Dichloro-2,6-dinitroanisole (***10***)

A solution of 3,4-dichloroanisole (**9**) (0.40 mL, 3.0 mmol) in 96% H_2_SO_4_ (5 mL) was cooled to −10 °C and KNO_3_ (0.67 g, 6.6 mmol) was added portion-wise over 20 min. The resulting suspension was stirred overnight at room temperature. The completion of the reaction was confirmed by TLC using system D. Crushed ice (20 g) was then added to the reaction mixture and neutralization followed to pH 6–7 with saturated aqueous NaOH solution. The resulting mixture was extracted twice with ethyl acetate. The organic phase was washed once with H_2_O and twice with brine and then dried over Na_2_SO_4_ and evaporated to dryness to give pure product **10**. Brown solid. Yield 0.74 g (92%). M.p. 55.5–57.0 °C. R*_f_* (D): 0.40. ^1^H-NMR (600 MHz, CDCl_3_): *δ*_H_ 8.22 (s, 1H, H-5), 4.05 (s, 3H, OMe) ppm. ^13^C-NMR (150 MHz, CDCl_3_): *δ*_C_ 145.7, 130.6, 129.6, 127.4, 65.3 ppm. Elemental analysis for C_7_H_4_Cl_2_N_2_O_5_: calcd C 31.49, H 1.51, N 10.49; found C 31.62, H 1.38, N 10.29.

### 2.3. 4,7,8-Trichloro-1-methoxy-2-nitrodibenzo[b,e][1,4]dioxine (***12***)

To a solution of 4,5-dichlorocatechol (**11**) (0.11 g, 0.60 mmol) in acetone (1.2 mL), K_2_CO_3_ (0.17 g, 1.2 mmol) was added under an inert atmosphere. The mixture was refluxed for 30 min, after which 3,4-dichloro-2,6-dinitroanisole (**10**) (0.16 g, 0.60 mmol) in acetone (1.2 mL) was added. The resulting mixture was refluxed for 2 h. The completion of the reaction was confirmed by TLC using system L. The reaction mixture was then diluted with H_2_O and extracted with ethyl acetate. The aqueous phase was re-extracted twice with ethyl acetate and the combined organic phases were washed once with H_2_O, and once with brine, and then dried over Na_2_SO_4_ and evaporated to dryness. FCC of the residue, using system E for elution, gave pure product **12**. Slightly yellow solid. Yield 0.15 g (70%). M.p. 203.5–205.5 °C. R*_f_* (E): 0.36. ^1^H-NMR (600 MHz, CDCl_3_): *δ*_H_ 7.62 (s, 1H, H-3), 7.16 (s, 1H, H-6/9), 7.13 (s, 1H, H-9/6), 4.02 (s, 3H, OMe) ppm. ^13^C-NMR (150 MHz, CDCl_3_): *δ*_C_ 142.6, 141.8, 139.6, 139.2, 139.2, 137.4, 128.7, 128.3, 120.7, 118.5, 118.2, 116.4, 62.8 ppm. Elemental analysis for C_13_H_6_Cl_3_NO_5_: calcd C 43.07, H 1.67, N 3.86; found C 42.91, H 1.84, N 3.99.

### 2.4. 4,7,8-Trichloro-1-methoxydibenzo[b,e][1,4]dioxin-2-amine (***13***)

A suspension of compound **12** (0.14 g, 0.39 mmol), FeCl_3_∙6H_2_O (3.0 mg) and activated C (3.0 mg) in MeOH (3.4 mL) and H_2_O (0.80 mL) was heated to 70 °C. Then H_2_NNH_2_∙H_2_O (90 μL, 1.90 mmol) was added and the reaction mixture was stirred under reflux for 3 h. The progress of the reaction was monitored by TLC using system E. Then, additional H_2_NNH_2_∙H_2_O (90 μL, 1.90 mmol) was added and the reaction mixture was further refluxed for additional 2 h. Following confirmation of the completion of the reaction, the resulting reaction mixture was diluted with ethyl acetate and washed twice with 5% aq. NaHCO_3_ and twice with brine. The organic layer was dried over Na_2_SO_4_ and evaporated to dryness. FCC of the residue, using system F for elution, gave pure product **13**. Beige solid. Yield 91 mg (70%). M.p. 196–199 °C. R*_f_* (F): 0.17. ^1^H-NMR (600 MHz, CDCl_3_): *δ*_H_ 7.07 (s, 1H, H-6/9), 7.06 (s, 1H, H-9/6), 6.38 (s, 1H, H-3), 3.87 (s, 3H) ppm. ^13^C-NMR (150 MHz, CDCl_3_): *δ*_C_ 140.8, 140.0, 136.7, 136.2, 133.8, 130.5, 127.1, 126.6, 118.1, 117.8, 115.9, 109.7, 60.8 ppm. Elemental analysis for C_13_H_8_Cl_3_NO_3_: calcd C 46.95, H 2.42, N 4.21; found C 47.11, H 2.23, N 4.14.

### 2.5. 2,4,7,8-Tetrachloro-1-methoxydibenzo[b,e][1,4]dioxine (***14***)

a. Preparation of the copper(I) chloride solution

Copper(II) sulfate pentahydrate (80 mg, 0.32 mmol) were dissolved in H_2_O (0.30 mL) in a reaction tube placed in a steam bath and then NaCl (38 mg, 0.64 mmol) was added. A solution of Na_2_SO_3_ (16 mg, 0.13 mmol) in H_2_O (0.10 mL) was then added dropwise to the hot copper(II) sulfate solution. The reaction mixture was stirred well, and then allowed to cool in ice while diazotizing the amine. When the copper(I) chloride was ready for use, H_2_O above the solid was removed, the white solid was washed once with H_2_O, H_2_O was then removed again, and the solid was finally dissolved in 37% HCl (0.15 mL).

b. Diazotization of aryl amine **13**

To an ice-cold solution of compound **13** (90 mg, 0.27 mmol) in 37% HCl (1.0 mL), an ice-cold solution of NaNO_2_ (20 mg, 0.28 mmol) in H_2_O (0.10 mL) was added. The yellow reaction mixture was stirred at 0 °C for 30 min, whereby the completion of the reaction was confirmed by TLC using system F.

c. Sandmeyer reaction

The cooled copper(I) chloride solution was added to the diazonium chloride solution dropwise with thorough mixing. The brownish reaction mixture was then let to reach the ambient temperature and bubbles of nitrogen gas were evolved. The reaction mixture was placed in a steam bath for 30 min. To the resulting yellow mixture H_2_O (2.0 mL) was added and further stirred at the refluxing temperature for 40 min whereby completion of the reaction was confirmed by TLC, using eluent G.

The mixture was then diluted with H_2_O and extracted with dichloromethane (DCM). The aqueous phase was re-extracted twice with DCM and then the combined organic layers were washed once with H_2_O and once with brine. Drying over Na_2_SO_4_ and evaporation to dryness left a residue. Finally, FCC of the residue, using system A for elution, gave pure product **14**. White solid. Yield 60 mg (63%). M.p. 164.5–165.5 °C. R*_f_* (A): 0.21. ^1^H-NMR (600 MHz, CDCl_3_): *δ*_H_ 7.11 (s, 1H, H-3), 7.09 (s, 1H, H-6/9), 7.05 (s, 1H, H-9/6), 3.91 (s, 3H, OMe) ppm. ^13^C-NMR (150 MHz, CDCl_3_): *δ*_C_ 143.3, 140.0, 139.8, 137.7, 137.0, 127.7, 127.6, 124.3, 123.3, 118.3, 118.0, 116.5, 61.5 ppm. Elemental analysis for C_13_H_6_Cl_4_O_3_: calcd C 44.36, H 1.72; found C 44.55, H 1.48.

### 2.6. 2,4,7,8-Tetrachlorodibenzo[b,e][1,4]dioxin-1-ol (***15***)

A solution of compound **14** (60 mg, 0.17 mmol) in dry DCM (0.15 mL) was cooled to −10 °C under an inert atmosphere and then 1 M BBr_3_ in DCM (0.34 mL) was added. The reaction mixture was stirred at that temperature for 30 min and then at ambient temperature for 2 h. The completion of the reaction was confirmed by TLC using system B. The reaction mixture was then diluted with DCM and washed carefully twice with 5% aqueous NaHCO_3_. The organic phase was subsequently washed once with 0.6 N aqueous HCl and twice with H_2_O. Drying over Na_2_SO_4_ and evaporation to dryness left pure product **15**. White solid. Yield 50 mg (87%). M.p. 207.5–210 °C; R*_f_* (G): 0.30. ^1^H-NMR (600 MHz, CDCl_3_): *δ*_H_ 7.13 (s, 1H, H-6/9), 7.09 (s, 1H, H-9/6), 7.03 (s, 1H, H-3), 5.50 (br. s, 1H, OH) ppm. ^13^C-NMR (150 MHz, CDCl_3_): *δ*_C_ 139.9, 139.7, 139.4, 137.4, 131.1, 127.8, 127.7, 123.8, 118.4, 118.0, 115.7, 112.8 ppm. Elemental analysis for C_12_H_4_Cl_4_O_3_: calcd C 42.73, H 1.12; found C 42.45, H 1.38.

### 2.7. 5,5-Dimethyl-2-(1-((3-((2,4,7,8-tetrachlorodibenzo[b,e][1,4]dioxin-1-yl)oxy)propyl) amino)ethylidene)cyclohexane-1,3-dione (***16***)

A suspension of compound **15** (47 mg, 0.14 mmol), *N*-Dde-protected 3-bromo-propylamine (45 mg, 0.15 mmol) and K_2_CO_3_ (58 mg, 0.42 mmol) in dry DMF (1.0 mL) was stirred vigorously at ambient temperature for 2 h under an inert atmosphere. The completion of the reaction was confirmed by TLC using the solvent system L. The reaction mixture was directly applied on the top of a chromatography column and subjected to FCC, using system M for elution, to give pure compound **16**. Beige solid. Yield 67 mg (86%). M.p. 153–155 °C. R*_f_* (M): 0.42. ^1^H-NMR (600 MHz, CDCl_3_): *δ*_H_ 13.65 (br. s, 1H, NH), 7.11 (s, 1H, H-3), 7.10 (s, 1H, H-6/9), 7.05 (s, 1H, H-9/6), 4.15 (t, ^3^*J_H,H_* = 6 Hz, 2H, OC*H*_2_)), 3.74 (q, ^3^*J_H,H_* = 6 Hz, 2H, NHC*H*_2_), 2.64 (s, 3H, =CC*H*_3_), 2.38 (s, 4H, two C*H*_2_CO), 2.17 (quint., ^3^*J_H,H_* = 6 Hz, 2H, CH_2_C*H*_2_CH_2_) 1.03 (s, 6H, two CC*H*_3_) ppm. ^13^C-NMR (150 MHz, CDCl_3_): *δ*_C_ 173.8, 141.9, 139.9, 139.7, 137.7, 137.0, 127.8, 127.6, 124.4, 123.1, 118.2, 118.1, 116.8, 108.0, 70.3, 39.7, 30.1, 29.7, 28.3, 17.9 ppm. Elemental analysis for C_25_H_23_Cl_4_NO_5_: calcd C 53.69, H 4.15, N 2.50; found C 53.49, H 4.01, N 2.34.

Note: Ιn the ^13^C-NMR spectrum of compound **16**, the two *C*O and the two CH_2_CO carbons of the Dde protecting group were not observed.

### 2.8. 3-((2,4,7,8-Tetrachlorodibenzo[b,e][1,4]dioxin-1-yl)oxy)propan-1-amine (***17***)

To a solution of compound **16** (56 mg, 0.10 mmol) in DMF (1.3 mL), H_2_NNH_2_∙H_2_O (30 μL, 0.50 mmol) was added. The reaction mixture was stirred at ambient temperature for 30 min. The progress of the reaction was monitored by TLC using solvent system M as eluant. The reaction mixture was then diluted with DCM and washed twice with H_2_O and once with brine. The organic layer was dried over Na_2_SO_4_ and evaporated to dryness to give pure product **17**, following FCC purification using system Q. Slightly yellow solid. Yield 32 mg (80%). M.p. 109–110.5 °C. R*_f_* (Q): 0.46. ^1^H-NMR (600 MHz, CDCl_3_): *δ*_H_ 7.10 (s, 1H, H-3), 7.06 (s, 1H, H-6/9), 7.04 (s, 1H, H-9/6), 4.12 (t, ^3^*J_H,H_* = 6 Hz, 2H, OC*H*_2_), 3.00 (t, ^3^*J_H,H_* = 6 Hz, 2H, NHC*H*_2_), 1.95 (quint. ^3^*J_H,H_* = 6 Hz, 2H, CH_2_C*H*_2_CH_2_) 1.70 (br. s, 2H, NH_2_) ppm. ^13^C-NMR (150 MHz, CDCl_3_): *δ*_C_ 142.4, 140.0, 139.8, 137.7, 137.0, 127.7, 127.6, 124.4, 123.4, 118.3, 118.0, 116.4, 72.5, 39.1, 33.7 ppm; MS (ESI, 30 eV): *m/z* 394.18, 396.20, 398.22 and 399.41 [M+H]^+^. Elemental analysis for C_15_H_11_Cl_4_NO_3_: calcd C 45.60, H 2.81, N 3.55; found C 45.35, H 3.02, N 3.65.

### 2.9. Conjugate ***18***-Attachment of Amino-Substituted Dioxin ***17*** on the Carboxy-Substituted Magnetic Beads

For the immobilization process [13], 2.5 mg of carboxy-substituted magnetic beads (0.242–0.285 μmole carboxylic acid) were used and treated according to the manufacturer’s instructions. Briefly, the beads were washed twice in DMF (1.5 mL each) and re-suspended in 1.1 mL of the same solvent. Subsequently 0.6 mg (2.91 μmole) of *N,N’*-dicyclohexylcarbodiimide (DCC) and 1 mg (2.53 μmole) of amino-substituted dioxin **17**, each dissolved in 0.2 mL DMF, were added sequentially to the beads and the resulting reaction mixture was incubated at 37 °C for 24 h with gentle agitation to ensure complete dispersion of the beads. Following removal of the reaction medium, the unreacted carboxyl groups on the beads were then quenched with 1.5 mL of a 80 mM ethanolamine solution in DMF at ambient temperature for 1 h. Finally, the beads were washed twice with DMF, re-suspended in DMF and stored at 4 °C. As a negative control, 2.5 mg of magnetic beads were coated with ethanolamine only using 1.5 mL of a 80 mM ethanolamine solution in DMF, containing 0.6 mg DCC, for 1 h at ambient temperature.

To assess the amount of amino-substituted dioxin **17** immobilized on the magnetic beads, the UV spectra of amino-substituted dioxin **17** and of aliquots of the coupling solution were recorded immediately after the addition of DCC and amino-substituted dioxin **17** and after the 24 h incubation at 37 °C (see Supplementary Materials, Figures S1 and S2). Taking into consideration the UV cutoff wavelength of DMF (268 nm), the measurement of the concentration of amino-substituted dioxin **17** before and after coupling was carried at λ_max_ 303 with ε_max_ 2173 mol^−1^·cm^−1^. The amount of amino-substituted dioxin **17** immobilized on the magnetic beads (2.5 mg) was then calculated indirectly and found to be 0.27 μmole (0.107 mg).

When the experiment was repeated at lower temperature (25 °C), using less excess of amino-substituted dioxin **17** (0.76 μmole, 0.3 mg) and shorter incubation time (12 h), significantly less loading (0.101 μmole, 0.04 mg) of amino-substituted dioxin **17** immobilized on the magnetic beads was affected.

Aliquots of uncoated magnetic beads, dioxin-coated magnetic beads and ethanolamine-coated magnetic beads were washed sequentially with DMF and diethyl ether and then, following removal of the solvents, dried overnight at ambient temperature over P_2_O_5_. For these samples and a sample of the amino-substituted dioxin **17** powder, ATR-FTIR spectra were recorded (see Supplementary Materials, Figures S3–S6) in an effort to provide additional qualitative evidence for the immobilization of amino-substituted dioxin **17** and/or ethanolamine on the magnetic beads [13]. The IR spectra were recorded at a resolution of 4 cm^−1^ and were averaged over 100 scans. The anticipated C_aryl_-Cl stretching frequencies in the area 1100–1035 cm^−1^ of the IR spectrum of dioxin-coated magnetic beads could not be however identified with certainty. On the other hand, changes noticed in the IR spectrum of uncoated magnetic beads when coated with amino-substituted dioxin **17** and/or ethanolamine might be taken to indicate immobilization of amino-substituted dioxin **17** and/or ethanolamine on the beads.

### 2.10. 5,5-Dimethyl-2-(1-((3-((2,4,7,8-tetrachlorodibenzo[b,d]furan-3-yl)oxy)propyl)ami-no)ethylidene)cyclohexane-1,3-dione (***23***)

A suspension of compound **21** (23 mg, 0.07 mmol), *N*-Dde-protected 3-bromopropan-1-amine (24 mg, 0.08 mmol) and K_2_CO_3_ (29 mg, 0.21 mmol) in dry DMF (0.40 mL) was stirred vigorously at ambient temperature for 2 h under an inert atmosphere (Ar). Additional *N*-Dde-protected 3-bromopropan-1-amine (8.0 mg) was added and the reaction mixture was further stirred for 48 h. The completion of the reaction was confirmed by TLC using system E. The reaction mixture was directly applied on the top of a chromatography column and subjected to FCC, using system E initially and then eluent Q, to give compound **23**. White solid. Yield 18 mg (48%). R*_f_* (J) 0.34. NMR spectra for compound **23** are provided as supplementary material. From these spectra, compound **23** seemed to contain a small amount of its isomeric compound **24**. ^1^H-NMR (600 MHz, CDCl_3_): *δ*_H_ 13.64 (br.s, 1H, N*H*), 7.94 (s, 1H, H-1), 7.81 (s, 1H, H-9), 7.75 (s, 1H, H-6), 4.23 (t, ^3^*J_H,H_* = 6 Hz, 2H, OC*H*_2_), 3.84 (q, ^3^*J_H,H_* = 6 Hz, 2H, NHC*H*_2_), 2.67 (s, 4H, two COC*H*_2_), 2.40 (s, 3H, =CC*H*_3_), 2.27 (quint., ^3^*J_H,H_* = 6 Hz, 2H, CH_2_C*H*_2_CH_2_), 1.06 (s, 6H, two CC*H*_3_) ppm. ^13^C-NMR (150 MHz, CDCl_3_): *δ*_C_ 173.9, 155.0, 152.3, 151.0, 131.9, 128.2, 124.9, 123.2, 121.8, 120.3, 119.5, 114.1, 113.3, 108.1, 70.5, 52.9, 40.1 30.1, 29.8, 28.2, 18.0 ppm.

Note: Ιn the ^13^C-NMR spectrum of compound **23**, the two *C*O carbons of the Dde protecting group were not observed

### 2.11. 5,5-Dimethyl-2-(1-((3-((2,3,7,8-tetrachlorodibenzo[b,d]furan-4-yl)oxy)propyl)ami-no)ethylidene)cyclohexane-1,3-dione (***24***)

A suspension of compound **22** (16 mg, 0.05 mmol), *N*-Dde-protected 3-bromo-propylamine (18 mg, 0.06 mmol) and K_2_CO_3_ (21 mg, 0.15 mmol) in dry DMF (0.30 mL) was stirred vigorously at ambient temperature for 24 h under an inert atmosphere (Ar). The completion of the reaction was confirmed by TLC using system J. The reaction mixture was directly applied on the top of a chromatography column and subjected to FCC, using system J initially, then eluent O and finally eluent P, to give pure compound **24**. Colorless oil. Yield 22 mg (82%). R*_f_* (J) 0.35. ^1^H-NMR (600 MHz, CDCl_3_): *δ*_H_ 13.66 (br.s, 1H, N*H*), 7.95 (s, 1H, H-1), 7.73 (s, 1H, H-9), 7.71 (s, 1H, H-6), 4.57 (t, ^3^*J_H,H_* = 6 Hz, 2H, OC*H*_2_), 3.82 (q, ^3^*J_H,H_* = 6 Hz, 2H, NHC*H*_2_), 2.66 (s, 3H, =CC*H*_3_), 2.38 (br. s, 4H, two COC*H*_2_), 2.26 (quint., ^3^*J_H,H_* = 6 Hz, 2H, CH_2_C*H*_2_CH_2_), 1.05 (s, 6H, two CC*H*_3_) ppm. ^13^C-NMR (150 MHz, CDCl_3_): *δ*_C_ 173.9, 154.7, 146.8, 141.4, 132.2, 128.8, 128.1, 124.6, 123.5, 122.9, 121.8, 115.7, 114.2, 108.1, 70.2, 39.9, 30.1, 29.7, 28.3, 17.9 ppm.

Note: Ιn the ^13^C-NMR spectrum of compound **24**, the two *C*O and the two *C*H_2_CO carbons of the Dde protecting group were not observed.

## 3. Results and Discussion

PCDDs have been synthesized through the condensation of chlorinated catechols or their corresponding dipotassium salts and chlorinated aromatics, bearing two chlorine atoms in adjacent positions, at the refluxing point (189 °C) of dimethyl sulfoxide (DMSO) [18,19]. However, in our hands, the attempted direct condensation of the commercially available 4,5-dichlorocatechol and 2,4,5,6-anisole in the presence of KOH at refluxing DMSO failed to produce any detectable amount of the anticipated 1-methoxy-2,3,7,8-tetrachlorodibenzo-*p*-dioxin. It has been reported however that this reaction can take place under milder reaction conditions, e.g., using a weaker base (K_2_CO_3_) in hexamethylphosphoramide (HMPA) at 190 °C [20] or in refluxing acetone [18], in case the polychlorinated aromatics contain a nitro group, which obviously serves to facilitate the initial nucleophilic aromatic substitution step [18,20], acting at the same time acts as a leaving group.

**Scheme 1:**
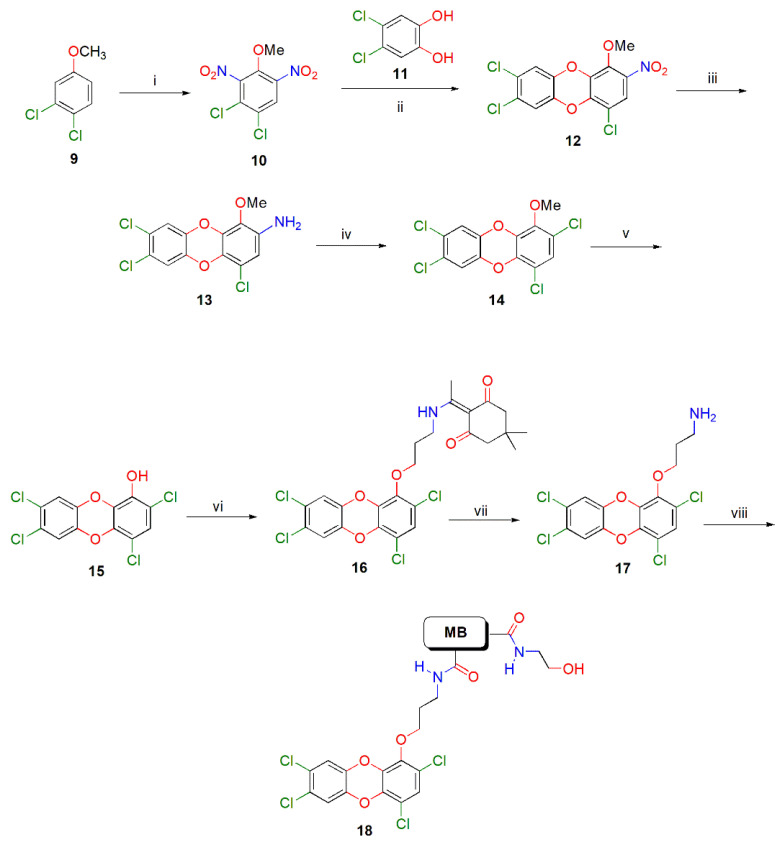
Synthesis of the 3-aminopropoxy-substituted tetrachlorodibenzo[*b,e*][1,4]dioxin **17** and conjugate **18** with carboxy-substituted magnetic beads thereof. *Reagents and conditions*: (i) KNO_3_/96% H_2_SO_4_, −10 °C, 1 h then 25 °C, 12 h, 92%; (ii) K_2_CO_3_, Me_2_C = O, 56 °C 2 h, 70%; (iii) N_2_H_4_·H_2_O, FeCl_3_·6H_2_O (cat.), activated C, 80% aq. MeOH, 65–70 °C, 5 h, 70%; (iv) NaNO_2_, 37% HCl, 0 °C, 30 min then Cu_2_Cl_2_, 100 °C, 40 min, 63%; (v) 1 M BBr_3_ in DCM, dry DCM, −10 °C, 30 min then 25 °C, 4 h, 87%; (vi) DdeNH(CH_2_)_3_Br, K_2_CO_3_, DMF, 25 °C, 2 h, 86%; (vii) 2% H_2_NNH_2_ in DMF, 25 °C, 30 min, 80%; (viii) Carboxy-substituted magnetic beads, DCC, DMF, 37 °C, 24 h, then 80 mM H_2_N(CH_2_)_2_OH in DMF, 25 °C, 1 h.

Taking into consideration that (a) the methoxy group is, in fact, a protected form of aromatic hydroxyl functions [20], (b) nitro groups in the polychlorinated aromatics facilitate the afore mentioned reaction, and (c) a nitro group can be readily replaced at a later stage by a chlorine atom through reduction and Sandmeyer reaction [21], we examined the condensation of 3,4-dichloro-2,6-dinitroanisole (**10**) with 4,5-dichlorocatechol (**11**) (Scheme 1). The former was readily obtained in 92% yield from 3,4-dichloanisole (**9**) upon nitration with the use of KNO_3_/96% H_2_SO_4_ system for 12 h at ambient temperature [22].

Next, anisole **10** and catechol **11** were condensed under mild conditions, that is using potassium carbonate as base in refluxing acetone for 2 h, yielding the anticipated substituted PCDD **12** in 70% yield. The reduction of nitro group of compound **12** was effected with hydrazine hydrate in the presence of ferric chloride trihydrate and activated C [22] to give the corresponding amino-substituted PCDD **13** in 70% yield. Compound **13**, was then subjected to a Sandmeyer chlorination reaction, using initially sodium nitrite and 37% aqueous HCl at 0 °C for 30 min and then Cu_2_Cl_2_ at 100 °C for 40 min, to give the methoxy-substituted tetrachlorodibenzo[*b,e*][1,4]dioxin (TCDD) **14** in 63% yield. From the latter compound, the methyl protecting group was removed using 1 M BBr_3_ in DCM at 0 °C for 30 min and then at ambient temperature for 4 h [22] to yield the hydroxyl-substituted TCDD **15** in 87% yield. The free phenolic hydroxyl was then subjected to Williamson etherification using the Dde-protected 3-bromopropan-1-amine [22] in DMF in the presence of potassium carbonate at ambient temperature for 2 h, affording the ether **16** in 86% yield. The resulting alkoxy-substituted TCDD **16** was then Dde-deprotected upon treatment with 2% hydrazine hydrate in DMF at ambient temperature for 30 min to provide, after purification with FCC, the targeted 3-(2,4,7,8-tetrachlorodibenzo[*b,e*][1,4]dioxin-1-yloxy)propan-1-amine (**17**) in 80% yield. Finally, coupling of amino-substituted dioxin **17** with carboxy-substituted magnetic beads (MB) in the presence of DCC, followed by capping of the unreacted carboxyl functions on the MB with ethanolamine, proceeded readily producing the targeted conjugate **18** (dioxin-coated magnetic beads) with high loading of dioxin **17**, as supported by means of UV spectroscopy measurements (see Materials and Methods and Supplementary Materials). The amount of immobilized dioxin **17** on the beads was calculated to be 0.108 μmole/mg beads whereas the capacity of the beads used was 0.097–0.114 μmole carboxylic acid/mg beads.

**Scheme 2:**
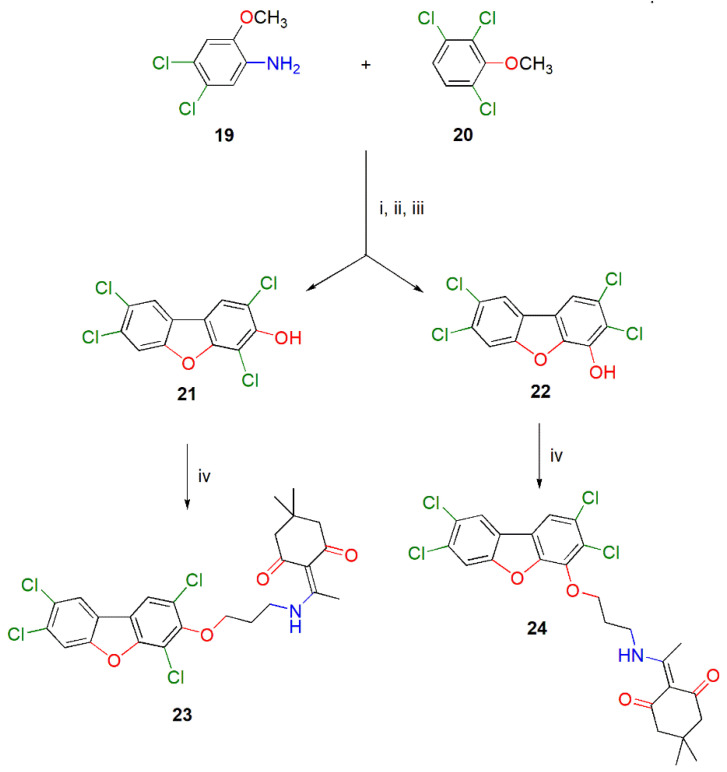
Synthesis of the N-Dde-protected 3-aminopropoxy-substituted tetrachlorodibenzo[*b,d*]furans **23** and **24**. *Reagents and conditions*: (i) Me_2_CHCH_2_CH_2_ON = O, 120 °C, 6 h, 16%; (ii) 1 M BBr_3_ in DCM, dry DCM, −10 °C, 30 min then 25 °C, 4 h then 40 °C, 1 h, 63%; (iii) K_2_CO_3_, DMSO, 155–160 °C, 8 h, then FCC, 39% (**21**) and 23% (**22**); (iv) DdeNH(CH_2_)_3_Br, K_2_CO_3_, DMF, 25 °C, 48 h for **21** or 24 h for **22**, 48% (**23**) and 82% (**24**).

In principle, the above described methodology could be easily adapted as well to the synthesis of the 3-aminoalkoxy-substituted tetrachlorodibenzo[*b,d*]furans (TCDFs). Starting materials for theses syntheses would be the corresponding hydroxyl-substituted TCDFs, for example compounds **21** and **22**. The synthesis of the regioisomeric 2,4,7,8-tetrachloro-dibenzo[*b,d*]furan-3-ol (**21**) and 2,3,7,8-tetrachlorodibenzo[*b,d*]furan-4-ol (**22**), which was performed according to reported procedure [17], is briefly depicted in Scheme 2 and fully detailed in the Supporting Materials. It involves the electrophilic substitution of 2,3,6-trichloroanisole (**20**) by the diazonium salt from 4,5-dichloro-*o*-anisidine (**19**), followed by deprotection of the phenolic hydroxyls and subsequent intramolecular nucleophilic aromatic substitution. For the needs of the present work, the required reactants **19** and **20** for the assembly of the tetrachlorodibenzo[*b,d*]furan skeleton, were prepared from the commercially available 3,4-dichloroanisole (**9**) and 2,3,6-trichlorophenol (**S2**), respectively, as described in the Supplementary Materials and depicted in Scheme S1 therein.

The thus-obtained hydroxyl-substituted TCDFs **21** and **22** were then reacted separately with Dde-NH(CH_2_)_3_Br [16] in DMF in the presence of potassium carbonate at ambient temperature for 48 h and 24 h, respectively, to afford the anticipated N-Dde-protected 3-aminopropoxy-substituted TCDFs **23** and **24** in 48% and 82% yield, respectively. The lower yield and slower reaction times for the former compound might, most likely, be attributed to the steric hindrance promoted by the two adjacent chlorine atoms during the alkylation reaction. Compounds **23** and **24** are convenient sources of the corresponding 3-aminopropoxy-substituted TCDFs, which could be also attached to carboxy-substituted magnetic beads for the development of aptamers specific for TCDFs. We are currently embarked on the application of the presently described methodology for the synthesis of other ω-aminoalkoxy-substituted PCDDs and PCDFs, as well as the development of ssDNA aptamers specific for 3-aminopropoxy-substituted dioxins for use as molecular recognition elements in photonic biosensors with environmental/alimentary interest.

## 4. Conclusions

Syntheses of N-Dde-protected 3-aminoalkoxy-substituted tetrachlorodibenzo-*p*-dioxin (TCDD) and tetrachlorodibenzo[*b,d*]furans (TCDFs), were successfully designed and realized using readily available and cost-affordable simple starting materials, such as 3,4-dichloroanisole, 2,3,6-trichlorophenol, 4,5-dichlorocatechol and 3-aminopropan-1-ol, and established reactions. The aliphatic amino function of these novel TCDD and TCDF derivatives, which is connected to the TCDD and TCDF nuclei through a propoxy linker, can be readily unmasked through hydrazinolysis and condense efficiently with the carboxyl function of carboxy-substituted magnetic beads under carbodiimide activation, as this was exemplified with the case of the 3-aminoalkoxy-substituted TCDD **17**. The resulting dioxin-coated magnetic beads are expected to facilitate the development, for the first time to the best of our knowledge, of specific aptamers for these dioxins of environmental/alimentary interest, and their integration in photonic biosensors for the rapid and reliable quantification of dioxins in foods.

## Data Availability

Not applicable.

## Supplementary Materials

The following are available online at https://www.mdpi.com/article/10.3390/ma14164727/s1, Details for the synthesis of hydroxyl-substituted TCDFs. ^1^H-NMR and ^13^C-NMR spectra for synthesized compounds. UV and ATR-FTIR spectra related to the immobilization of amino-substituted dioxin **17** on carboxy-substituted magnetic beads. Scheme S1: Synthesis of hydroxy-substituted tetrachlorodibenzo[*b,d*]furans **21** and **22**. *Reagents and conditions*: (i) KNO_3_, 96% H_2_SO_4_, −10 °C, 1 h then 25 °C, 12 h, 38%; (ii) N_2_H_4_·H_2_O, FeCl_3_·6H_2_O (cat.), activated C, 80% aq. MeOH, 65–70 °C, 5 h, 84%; (iii) MeOH, Ph_3_P, ^i^PrO_2_CN=NCO_2_^i^Pr (DIAD), dry THF, 0 °C then 25 °C, 1 h, 86%; (iv) Me_2_CHCH_2_CH_2_ON=O, 120 °C, 6 h, 16%; (v) 1M BBr_3_ in DCM, dry DCM, −10 °C, 30 min then 25 °C, 4 h then 40 °C, 1 h, 63%; (vi) K_2_CO_3_, DMSO, 155-160 °C, 8 h, then FCC, 39% (21) and 23% (22), Figure S1: The UV spectrum (250–350 nm) of amino-substituted dioxin **17** in DMF, Figure S2: The UV spectrum (275–350 nm), in DMF, of amino-substituted dioxin **17** before (green line) and after (red line) coupling with the carboxy-substituted magnetic beads, Figure S3: The ATR-FTIR spectrum of amino-substituted dioxin **17**, Figure S4: The ATR-FTIR spectrum of magnetic beads, Figure S5: The ATR-FTIR spectrum of magnetic beads coated with the amino-substituted dioxin **17**, Figure S6: The ATR-FTIR spectrum of magnetic beads coated with ethanolamine.
