# Supplementary material for: Studies towards the Synthesis of Novel 3-Aminopropoxy-Substituted Dioxins Suitable for the Development of Aptamers for Photonic Biosensor Applications
Source: Materials (Basel). 2021 Aug 21;14(16):4727. doi: 10.3390/ma14164727 (PMC8401930; doi:10.3390/ma14164727)
Supplement: Supplementary file 1 [file materials-14-04727-s001.zip › materials-1312053-SI.pdf]

Supplementary Materials

Studies towards the Synthesis of Novel 3-Aminopropoxy-SubStituted Dioxins Suitable for the Development of Aptamers for Photonic Biosensor Applications

Stefania Kalantzi [1], Sofia Leonardi [1], Eleanna Vachlioti [1], Eleni G. Kaliatsi [2], Konstantina Papachristopoulou [3], Constantinos Stathopoulos [2], Nikolaos Vainos [3] and Dionissios Papaioannou [1,*]

[1] Laboratory of Synthetic Organic Chemistry, Department of Chemistry, University of Patras, GR-26504 Patras, Greece; s.kalantzi8@gmail.com (S.K.); ssofiaa30@gmail.com (S.L.); eleannavah@yahoo.gr (E.V.)
[2] Department of Biochemistry, School of Medicine, University of Patras, GR-26504 Patras, Greece; lenakaliatsi@hotmail.gr (E.G.K.); cstath@upatras.gr (C.S.)
[3] Photonics Nanotechnology Research Laboratory, Department of Materials Science, University of Patras, GR-26504 Patras, Greece; k.papachristopoulou@upnet.gr (K.P.); vainos@upatras.gr (N.V.)
* Correspondence: dapapaio@upatras.gr; Tel.: +30-2610-962954

Table of Contents

Synthesis of hydroxy-substituted TCDFs ***21*** *and* ***22*** S-3

Experimental procedures S-6

Copy of 1H NMR spectrum of **10** S-9

Copy of ^{13}C NMR spectrum of **10** S-10

Copy of ^{1}H NMR spectrum of **12** S-11

Copy of ^{13}C NMR spectrum of **12** S-12

Copy of ^{1}H NMR spectrum of **13** S-13

Copy of ^{13}C NMR spectrum of **13** S-14

Copy of ^{1}H NMR spectrum of **14** S-15

Copy of ^{13}C NMR spectrum of **14** S-16

Copy of ^{1}H NMR spectrum of **15** S-17

Copy of ^{13}C NMR spectrum of **15** S-18

Copy of ^{1}H NMR spectrum of **16** S-19

Copy of ^{13}C NMR spectrum of **16** S-20

Copy of ^{1}H NMR spectrum of **17** S-21

Copy of ^{13}C NMR spectrum of **17** S-22

Copy of ^{1}H NMR spectrum of **S1** S-23

Copy of ^{13}C NMR spectrum of **S1** S-24

Copy of ^{1}H NMR spectrum of **19** S-25

Copy of ^{13}C NMR spectrum of **19** S-26

Copy of ^{1}H NMR spectrum of **20** S-27

Copy of ^{13}C NMR spectrum of **20** S-28

Copy of ^{1}H NMR spectrum of **S3** and **S4** S-29

Copy of ^{13}C NMR spectrum of **S3** and **S4** S-30

Copy of ^{1}H NMR spectrum of **S5** and **S6** S-31

Copy of ^{13}C NMR spectrum of **S5** and **S6** S-32

Copy of ^{1}H NMR spectrum of **21** S-33

Copy of ^{13}C NMR spectrum of **21** S-34

Copy of ^{1}H NMR spectrum of **22** S-35

Copy of ^{13}C NMR spectrum of **22** S-36

Copy of ^{1}H NMR spectrum of **23** S-37

Copy of ^{13}C NMR spectrum of **23** S-38

Copy of ^{1}H NMR spectrum of **24** S-39

Copy of ^{13}C NMR spectrum of **24** S-40

Figure S1 S-41

Figure S2 S-42

Figure S3 S-43

Figure S4 S-44

Figure S5 S-45

Figure S6 S-46

Synthesis of hydroxy-substituted tetrachlorodibenzo[*b*,*d*]furans (TCDFs) 21 and 22

The synthesis of the hydroxy-substituted-TCDFs **21** and **22** was effected according to a reported methodology using as key-step the electrophilic substitution of 2,3,6-trichloroanisole (**20**) by the diazonium salt from 4,5-dichloro-*o*-anisidine (**19**), followed by deprotection of the phenolic hydroxyls and subsequent intramolecular nucleophilic aromatic substitution [S1]. In the context of the present work, the required reactants for the assembly of the skeleton, namely compounds **19** and **20**, were prepared from the commercially available 3,4-dichloroanisole (**9**) and 2,3,6-trichlorophenol (**S2**), respectively, as follows (Scheme S1). Nitration of compound **9** was attempted using the system KNO_3/conc. H_2SO_4 [S2], at 0 ^{o}C and then ambient temperature overnight, under the ratios substrate (**9**):KNO_3 = 1:2.2, 1:1.5, 1:1.25, 1:1 and 1:0.9. The ratio 1:2.2 produced exclusively the dinitro derivative **10** (Scheme 1), whereas the other ratios also produced the dinitro derivative as the main product along with 21%, 38%, 27% and 30% yield, respectively, of the targeted mononitro derivative **S1** (Scheme 1). Compound **10** was also the sole product of the nitration reaction of anisole **1** using the system ammonium nitrate/trifluoroacetic anhydride [S3]. The reduction of nitro compound **S1** was effected with hydrazine hydrate in the presence of ferric chloride trihydrate and activated C to give 4,5-dichloro-*o*-anisole (**19**) in 84% yield. On the other hand, protection of the hydroxyl group of phenol **S2** was realized as the corresponding methyl ether **20** in 86% yield upon subjecting the former compound to Mitsunobu reaction with methanol.

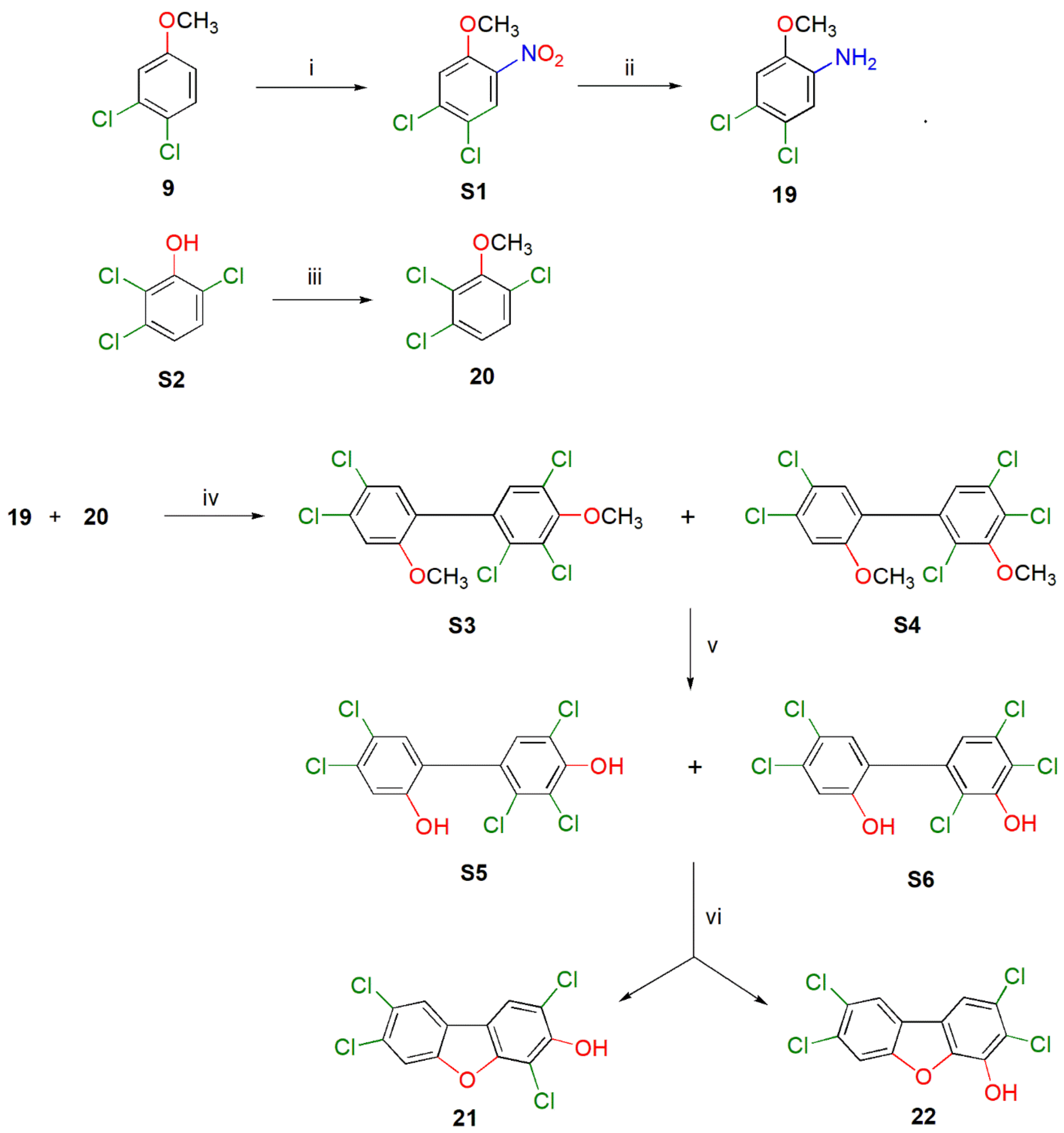

Scheme S1. Synthesis of hydroxy-substituted tetrachlorodibenzo[*b*,*d*]furans 21 and 22. *Reagents and conditions*: (i) KNO_3, 96% H_2SO_4, −10 °C, 1 h then 25 °C, 12 h, 38%; (ii) $N_2H_4{\cdot}H_2O$, $FeCl_3{\cdot}6H_2O$ (cat.), activated C, 80% aq. MeOH, 65–70 °C, 5 h, 84%; (iii) MeOH, Ph_3P, $^iPrO_2CN{=}NCO_2{}^iPr$ (DIAD), dry THF, 0 °C then 25 °C, 1 h, 86%; (iv) $Me_2CHCH_2CH_2ON{=}O$, 120 °C, 6 h, 16%; (v) 1M BBr_3 in DCM, dry DCM, -10 °C, 30 min then 25 °C, 4 h then 40 °C, 1 h, 63%; (vi) K_2CO_3, DMSO, 155-160 °C, 8 h, then FCC, 39% (**21**) and 23% (**22**).

Coupling of compounds **19** and **20** was effected on treating their neat mixture with isopentyl nitrite at 120 °C for 6 h to produce in 16% yield an equimolar mixture of the two substituted

biphenyls 2,3,4′,5,5′-pentachloro-2′,4-dimethoxy-1,1′-biphenyl (**S3**) and 2,4,4′,5,5′-pentachloro-2′,3-dimethoxy-1,1′-biphenyl (**S4**) [S1] which could not be separated by Flash Column Chromatography (FCC). This mixture was also found by H^1-NMR to contain a small amount of the side product 3,4-dichloroanisole (**9**), which obviously resulted from the reduction of the corresponding intermediate diazonium salt. The afore mentioned mixture was treated with a 1M solution of BBr_3 in dichloromethane (DCM) at −10 °C for 30 min and then sequentially at ambient temperature and reflux temperature for 4 h and 30 min, respectively [S2], to produce the also inseparable equimolar mixture of the corresponding dihydroxybiphenyls, namely 2,3,4′,5,5′-pentachloro-2′,4-dihydroxy-1,1′-biphenyl (**S5**) and 2,4,4′,5,5′-pentachloro-2′,3-dihydroxy-1,1′-biphenyl (**S6**), in 63% yield. This new mixture in DMSO was finally heated at 155–160 °C for 8 h, in the presence of potassium carbonate, to produce a crude mixture of the targeted hydroxy-substituted-TCDFs 2,4,7′,8′-tetrachlorodibenzo[*b,d*]furan-3-ol (**21**) and 2,3,7′,8′-tetrachlorodibenzo[*b,d*]furan-4-ol (**22**) [S1], from which the pure products (compounds **21** and **22**) could be now separated by FCC and isolated in 39% and 23% yields, respectively.

References

[S1]. Burka, L.T.; Overstreet, D. Synthesis of possible metabolites of 2,3,7,8-tetrachlorodibenzofuran. *J. Agric. Food Chem.* **1989**, *37*, 1528–1532.

[S2]. Rassias, G.; Leonardi, S.; Rigopoulou, D.; Vachlioti, E.; Afratis, K.; Piperigkou, Z.; Koutsakis, C.; Karamanos, N.K.; Gavras, H.; Papaioannou, D. Potent antiproliferative activity of bradykinin B2 receptor selective agonist FR-190997 and analogue structures thereof: A paradox resolved? *Eur. J. Med. Chem.* **2021**, *210*, 112948, https://doi.org/10.1016/j.ejmech.2020.112948.

[S3]. Oliver, J.E.; Ruth, J.M. Nitration of two TCDD's and their conversion to 1,2,3,6,7,8-HCDD. *Chemosphere* **1983**, *12*, 1497–1503.

Experimental procedures

*4,5-Dichloro-2-nitroanisole (**S1**)*

To a solution of 3,4-dichloroanisole (**9**) (1.5 g, 8.5 mmol) in conc. sulfuric acid (15 mL), kept at −10 °C, potassium nitrate (1.1 g, 10.7 mmol) was added portion-wise within 1 h. The reaction mixture was further stirred at ambient temperature for 12 h. Then ice (150 g) was introduced whereby precipitation takes place. Dilution with H_2O, extraction twice with ethyl acetate and washing of the combined organic phases once with an ice-cold 5% aqueous solution of $NaHCO_3$, once with H_2O and twice with brine, followed by drying (anhydrous Na_2SO_4) and evaporation under reduced pressure to dryness gave a brown oil, consisted of the product **S1** and the corresponding dinitro compound **10** (Scheme 1). FCC of the mixture, using initially the system C, then the system E and finally eluent G, provided pure compounds **S1** and **10** (0.75 g). Yellow crystals. Yield 0.72 g (38%). R_f(D): 0.25 for **S1** and 0.40 for **10**. ^{1}H-NMR (600 MHz, $CDCl_3$): δ_H 8.02 (s, 1H, H-3), 7.21 (s, 1H, H-6), 3.99 (s, 3H, OMe) ppm. ^{13}C-NMR (150 MHz, $CDCl_3$): δ_C 152.0, 138.7, 127.1, 124.1, 115.6, 57.2 ppm.

*4,5-Dichloro-*o*-anisidine (**19**)*

A suspension of nitroanisole **S1** (0.69 g, 3.10 mmol), $FeCl_3.6H_2O$ (25 mg) and activated C (25 mg) in MeOH (9 mL) and H_2O (2 mL) was heated at 65-70 °C and then hydrazine hydrate (0.37 mL, 7.60 mmol). The resulting mixture was stirred vigorously at the same temperature for 2 h and then three more times of 0.37 mL of hydrazine hydrate were added each time and the resulting mixture was stirred at that temperature for additional 1 h following each addition. Then it was diluted with ethyl acetate and extracted twice with a 5% aqueous solution of $NaHCO_3$ and twice with brine. The organic layer was dried over anhydrous Na_2SO_4 and evaporated to dryness leaving a solid residue. FCC of the residue using eluent G gave pure anisidine **19**. Pale yellow solid. Yield 0.5 g (84%). R_f(G): 0.33. ^{1}H-NMR (600 MHz, $CDCl_3$): δ_H 6.82 (s, 1H, H-3), 6.76 (s, 1H, H-6), 3.85 (s, 3H, OMe) ppm. ^{13}C-NMR (150 MHz, $CDCl_3$): δ_C 146.3, 136.0, 123.7, 120.2, 115.2, 112.1, 55.3 ppm.

*2,3,6-Trichloroanisole (**20**)*

To a solution of phenol **S2** (1.97 g, 10 mmol) in dry THF (20 mL) kept at 0 °C, Ph_3P (2.89 g, 11 mmol) and MeOH (0.61 mL, 15 mmol) were added sequentially, followed by the drop-wise

addition of DIAD (2.17 mL, 11 mmol). The reaction mixture was left to attain ambient temperature where it was kept for 1 h and then evaporated to dryness. Diethyl ether was added to the residue and the precipitated solid was filtered off and washed on the filter with cold diethyl ether. The filtrate was evaporated to dryness and the new residue was subjected to FCC, using the system B, to give pure anisole **20**. White solid. Yield 1.81 g (86 %). R_f(B): 0.53. ^{1}H-NMR (600 MHz, $CDCl_3$): δ_H 7.26 (d, $^3J_{H,H}$ = 8.4 Hz, 1H, H-5), 7.20 (d, $^3J_{H,H}$ = 8.4 Hz, 1H, H-4), 3.93 (s, 3H, OMe) ppm. ^{13}C-NMR (150 MHz, $CDCl_3$): δ_C 153.6, 132.2, 128.9, 128.4, 127.7, 125.9, 60.7 ppm.

*2,3,4′,5,5′-Pentachloro-2′,4-dimethoxy-1,1′-biphenyl (**S3**) and 2,4,4′,5,5′-pentachloro-3′,4-dimethoxy-1,1′-biphenyl (**S4**)*

To a stirred melted mixture of anisidine **19** (0.44 g, 2.3 mmol) and anisole **20** (2.09 g, 9.9 mmol) kept at 120 °C, was added isopentyl nitrite (0.50 mL, 3.7 mmol) portion-wise within 30 min. The resulting reaction mixture was further stirred at the same temperature for 6 h and at ambient temperature for 12 h. The solidified residue was subjected to FCC using system H for elution and the fractions with R_f 0.33, for the same system, were combined and evaporated to dryness to leave 0.14 g (ca. 16%) of an inseparable mixture of biphenyl isomers **S3** and **S4**, containing small quantities of the side product **9** (by ^{1}H-NMR), as a yellow oil. This mixture (NMR spectra are provided in pp. S-29 and S-30) was used as such into the next experiment.

*2,3,4′,5,5′-Pentachloro-1,1′-biphenyl-2′,4-diol (**S5**) and 2,4,4′,5,5′-pentachloro-1,1′-biphenyl-3′,4-diol (**S6**)*

To a cold (10 °C) solution of the afore mentioned mixture (0.14 g, 0.36 mmol) in dry DCM (0.20 mL) was added 1M BBr_3 in DCM (2.70 mL) and stirring was continued for 30 min at that temperature and for 2 h at ambient temperature. More 1M BBr_3 solution (0.90 mL) was introduced and stirring was continued for additional 2 h at ambient temperature and 1 h at the refluxing point. Dilution with DCM, careful washing with ice-cold 5% aqueous $NaHCO_3$ and then brine, drying (anhydrous Na_2SO_4) and evaporation under reduced pressure left an oily residue, which was subjected to FCC using system I. The fractions with R_f 0.16 for the same system were pooled and the solvents were evaporated under reduced pressure to leave 81 mg (63%) of an inseparable mixture of the regioisomeric diols **S5** and **S6** as a pale brown oil (NMR spectra are provided in pp. S-31 and S-32). This mixture was used as such into the next experiment.

*2,4,7,8-Tetrachlorodibenzo[*b,d*]furan-3-ol (***21***) and 2,3,7,8- dibenzo[*b,d*]furan-4-ol (***22***)*

To a solution of the afore mentioned mixture of diols **S5** and **S6** (81 mg, 0.23 mmol) in dry DMSO (1.5 mL) was added potassium carbonate (64 mg, 0.46 mmol) and the resulting mixture was stirred with heating at 155–160 ^{o}C for 8 h under argon. The reaction mixture was then diluted with aqueous 5% citric acid and extracted thrice with ethyl acetate. The combined organic layers were washed twice with water and once with brine, dried (anhydrous Na_2SO_4) and evaporated to dryness to leave a residue, which was subjected to FCC using system K. The fractions with R_f 0.16 for the same solvent system were pooled and solvents were removed under reduced pressure to give 29 mg (39%) of regioisomer **21**, whereas the fractions with R_f 0.10 were also pooled and solvents were removed under reduced pressure to give 17 mg (23%) regioisomer **22**. Both compounds were white solids. NMR spectra for both compounds are provided in pp. S-33 and S-34, and S-35 and S-36, respectively. Regioisomer **21** was found by ^{1}H-NMR to contain a small quantity of regioisomer **22** in the ratio 10:1, but used as such in the next experiment, that is Williamson etherification using the bromide DdeNH(CH_2)$_3$Br (see Materials and Methods section). It should be noted that the NMR spectra for regioisomer **22** were recorded in CD_3OD.

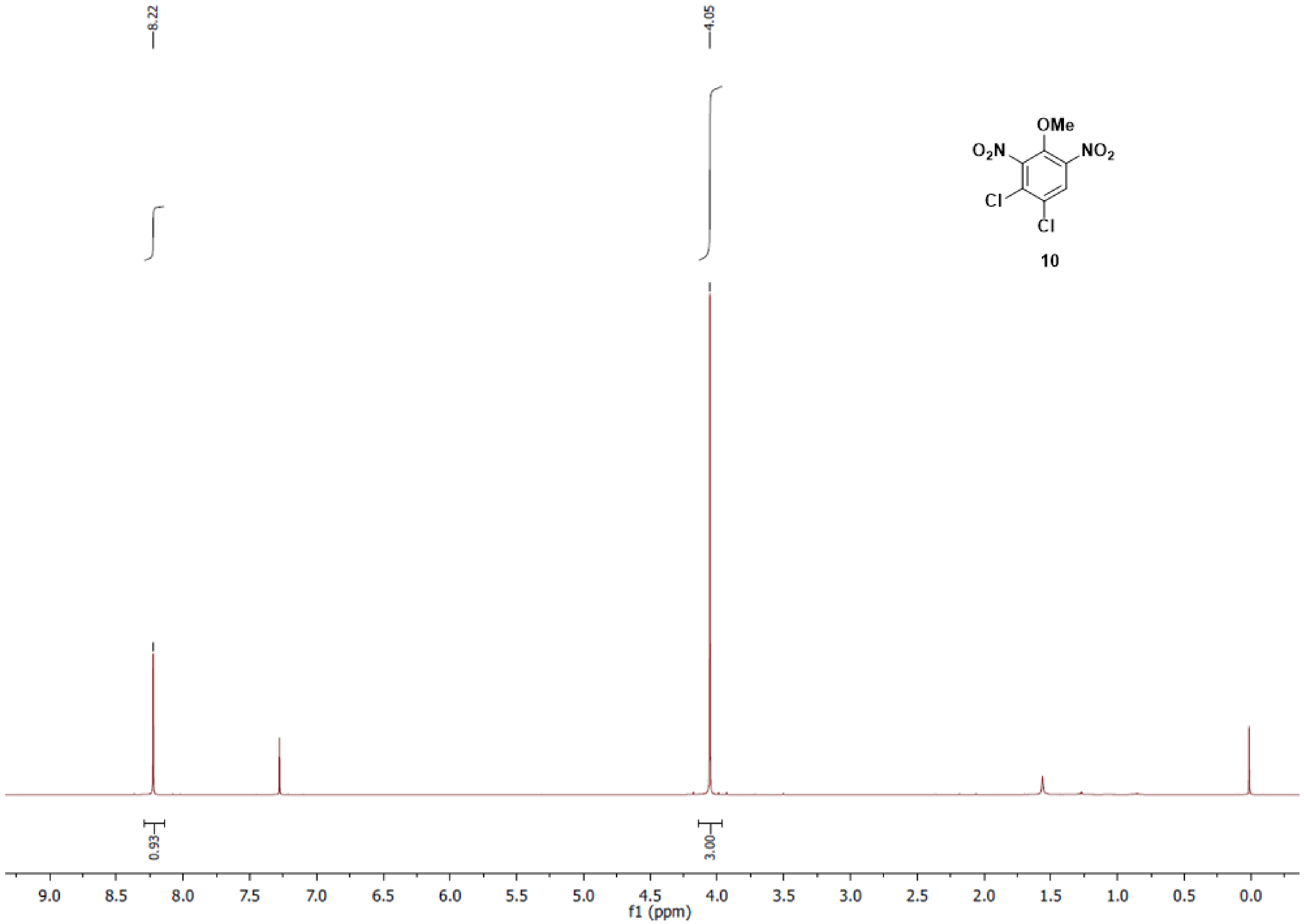
8.22
4.05
OMe
O2N
NO2
Cl
Cl
10
0.93
3.00
9.0
8.5
8.0
7.5
7.0
6.5
6.0
5.5
5.0
4.5
4.0
3.5
3.0
2.5
2.0
1.5
1.0
0.5
0.0
f1 (ppm)

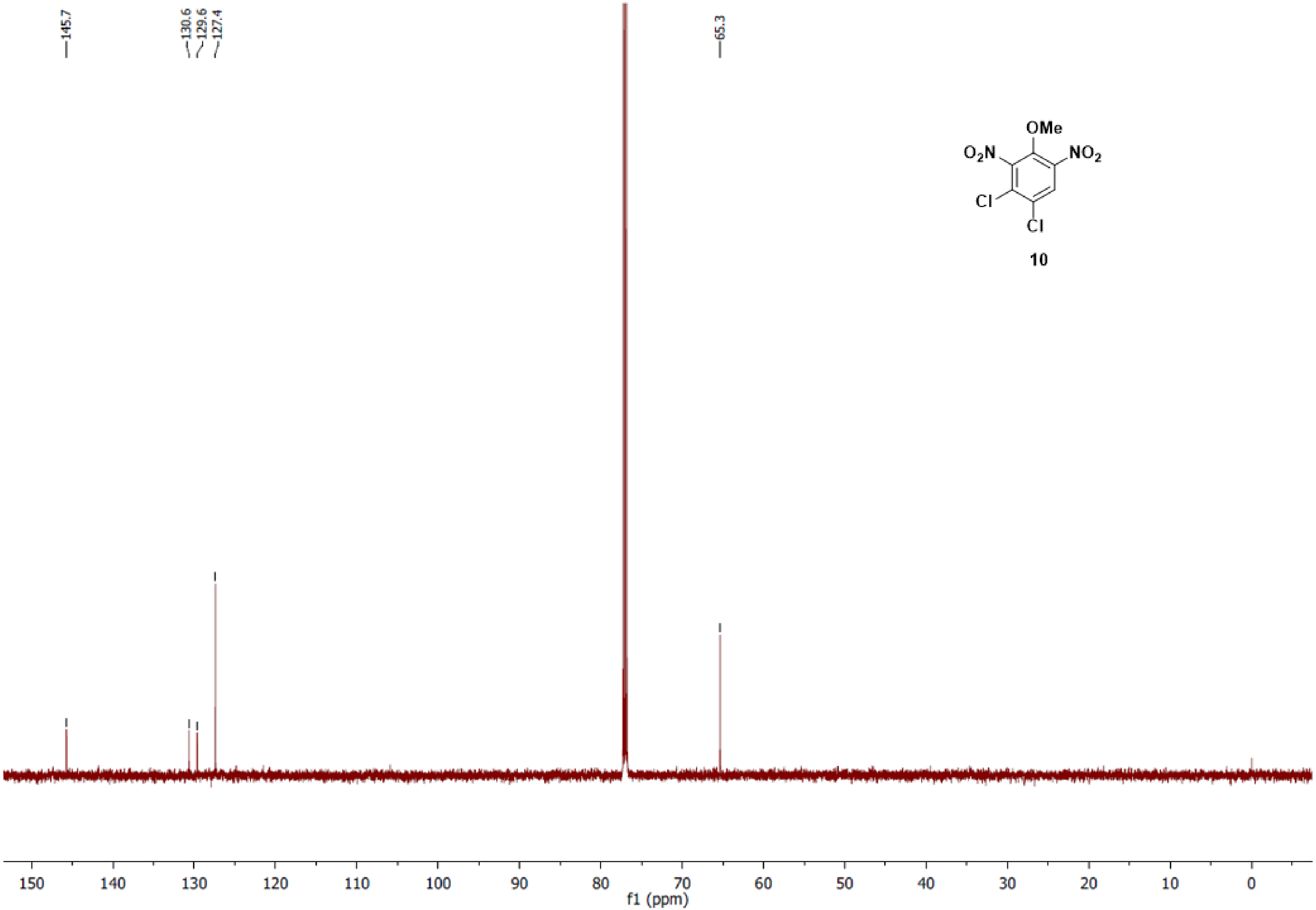
145.7
130.6
129.6
127.4
65.3
OMe
O_2N
NO_2
Cl
Cl
10
150 140 130 120 110 100 90 80 70 60 50 40 30 20 10 0
f1 (ppm)

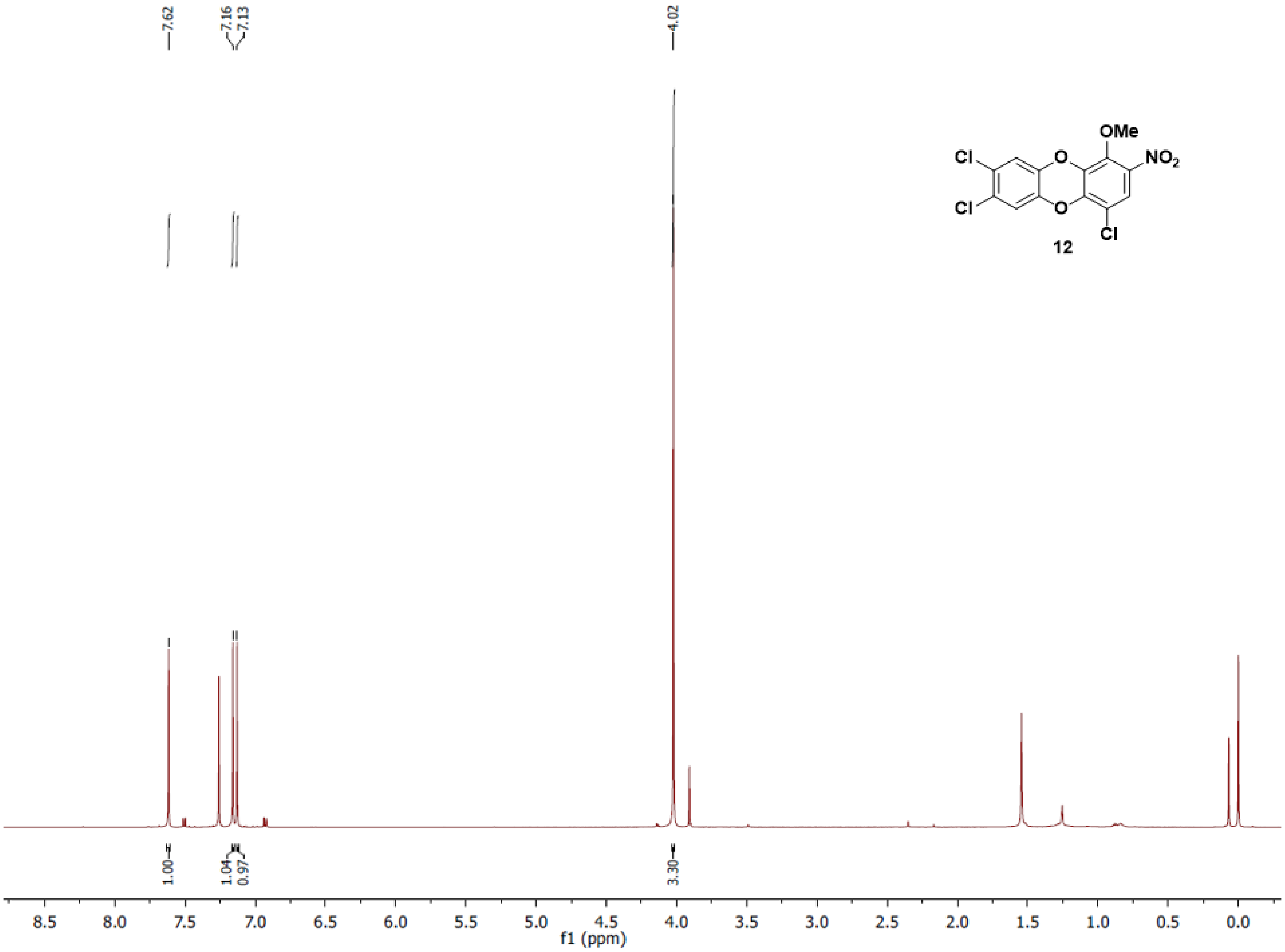

7.62
7.16
7.13
4.02
OMe
Cl
NO2
Cl
O
O
Cl
1.00
1.04
0.97
3.30
8.5
8.0
7.5
7.0
6.5
6.0
5.5
5.0
4.5
4.0
3.5
3.0
2.5
2.0
1.5
1.0
0.5
0.0
f1 (ppm)

12

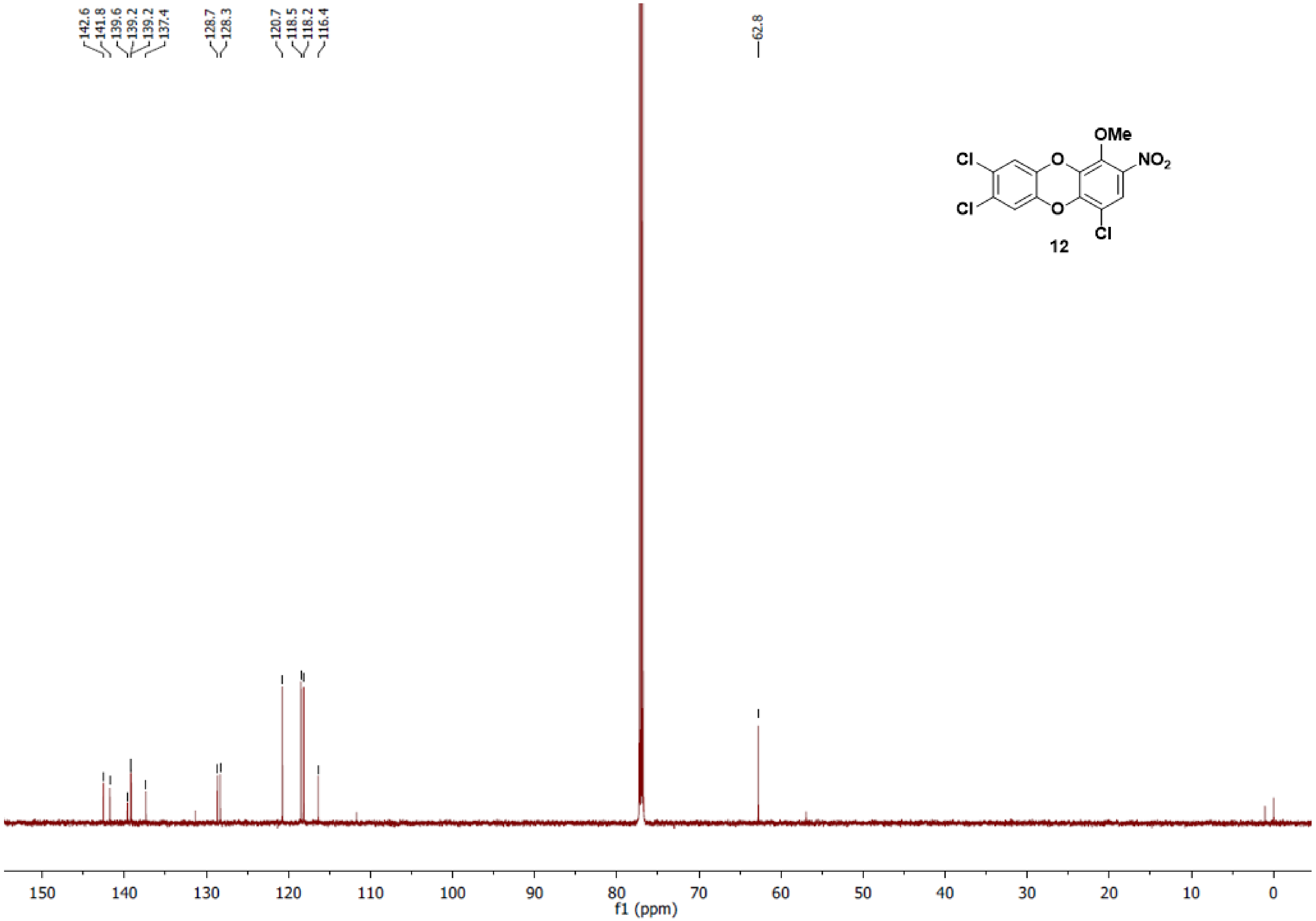

142.6
141.8
139.6
139.2
139.2
137.4
128.7
128.3
120.7
118.5
118.2
116.4
62.8
OMe
NO_2
Cl
Cl
Cl
O
O
12
150
140
130
120
110
100
90
80
70
60
50
40
30
20
10
0
f1 (ppm)

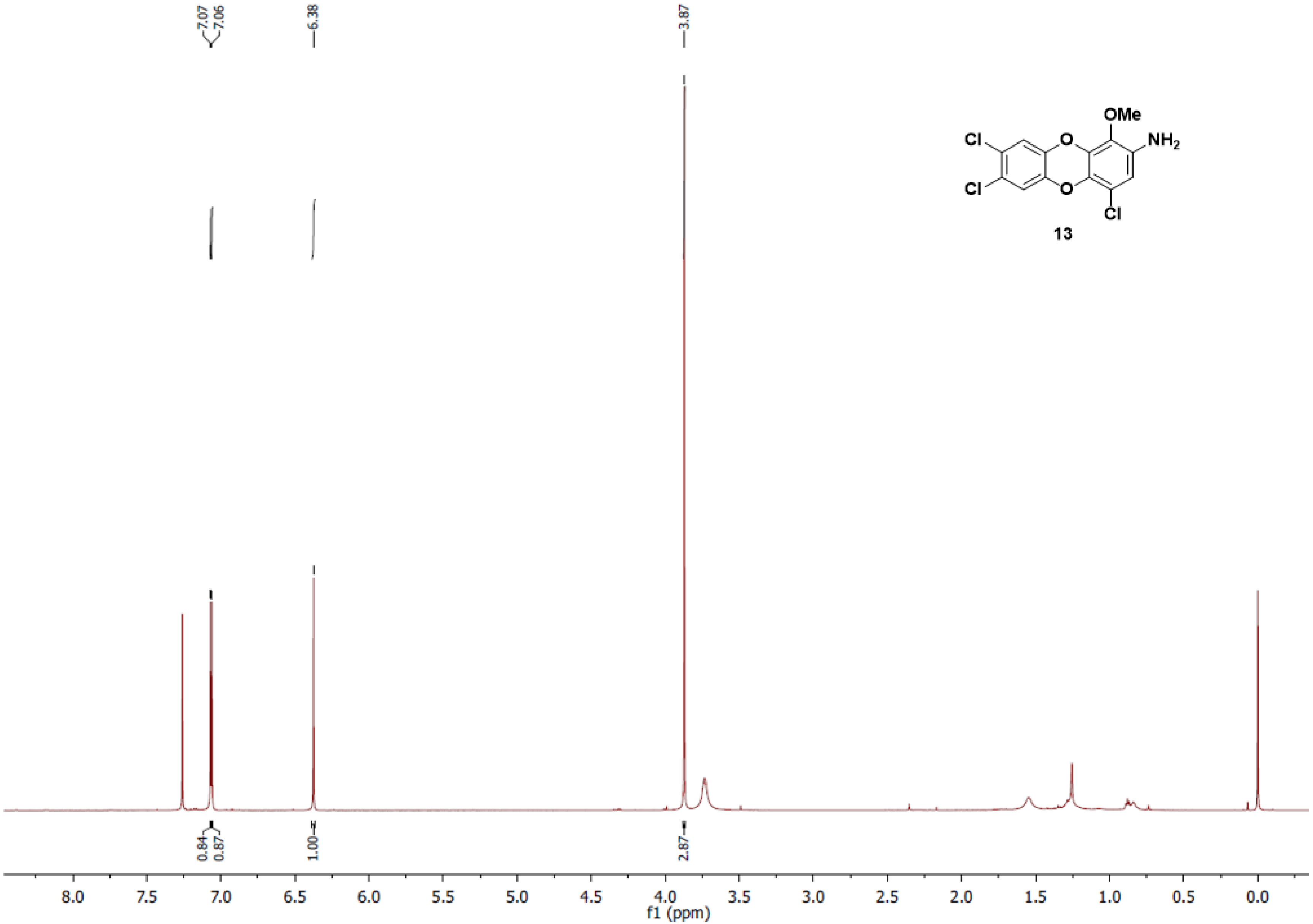

7.07
7.06
6.38
3.87
OMe
Cl
Cl
NH2
Cl
O
O
13
0.84
0.87
1.00
2.87
8.0
7.5
7.0
6.5
6.0
5.5
5.0
4.5
4.0
3.5
3.0
2.5
2.0
1.5
1.0
0.5
0.0
f1 (ppm)

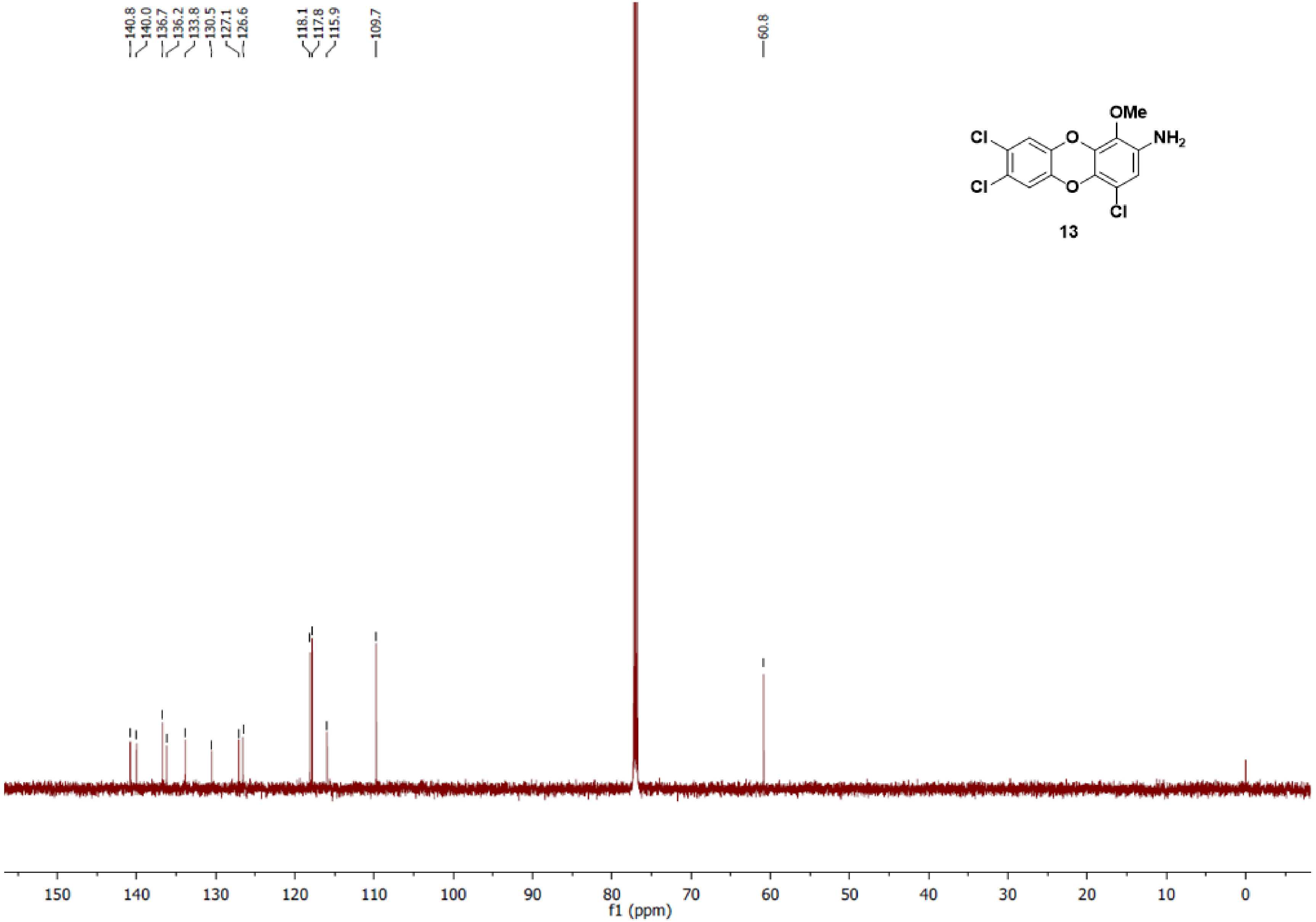
140.8
140.0
136.7
136.2
133.8
130.5
127.1
126.6
118.1
117.8
115.9
109.7
60.8
OMe
Cl
NH2
Cl
Cl
13
150
140
130
120
110
100
90
80
70
60
50
40
30
20
10
0
f1 (ppm)

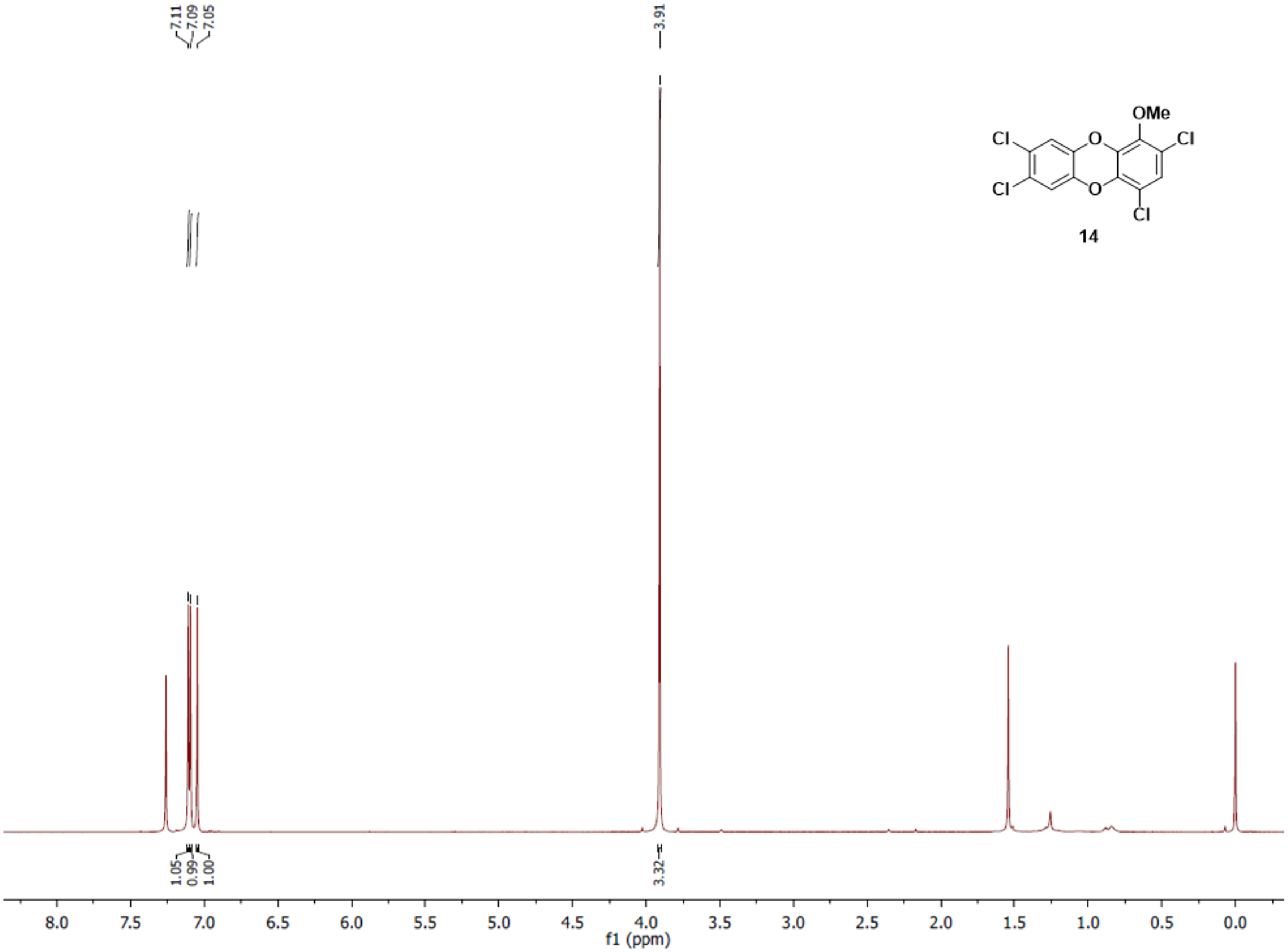
7.11
7.09
7.05
3.91
OMe
Cl
Cl
Cl
Cl
O
O
14
1.05
0.99
1.00
3.32
8.0 7.5 7.0 6.5 6.0 5.5 5.0 4.5 4.0 3.5 3.0 2.5 2.0 1.5 1.0 0.5 0.0
f1 (ppm)

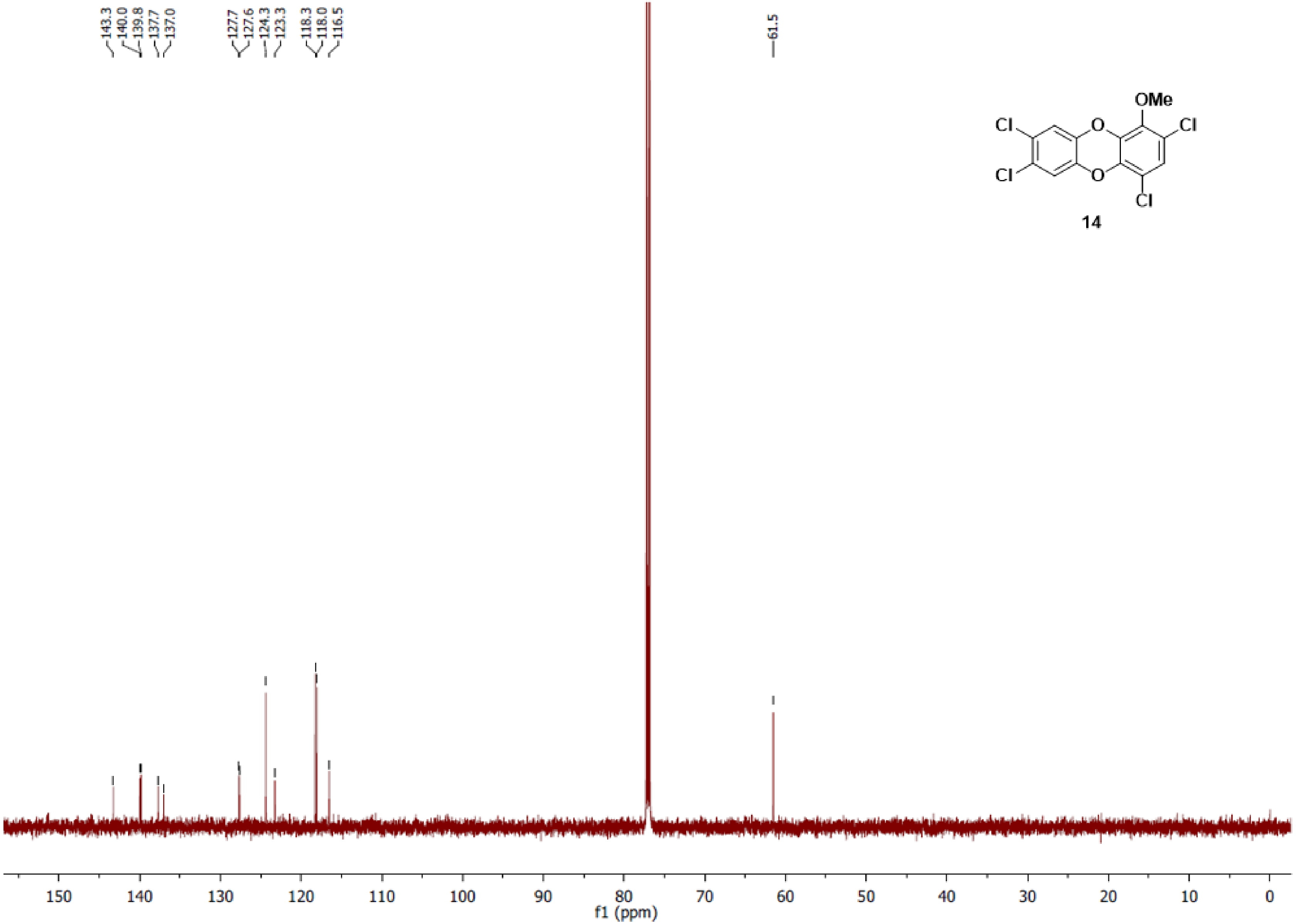
143.3
140.0
139.8
137.7
137.0
127.7
127.6
124.3
123.3
118.3
118.0
116.5
61.5
150
140
130
120
110
100
90
80
70
60
50
40
30
20
10
0
f1 (ppm)
OMe
Cl
Cl
Cl
Cl
O
O

14

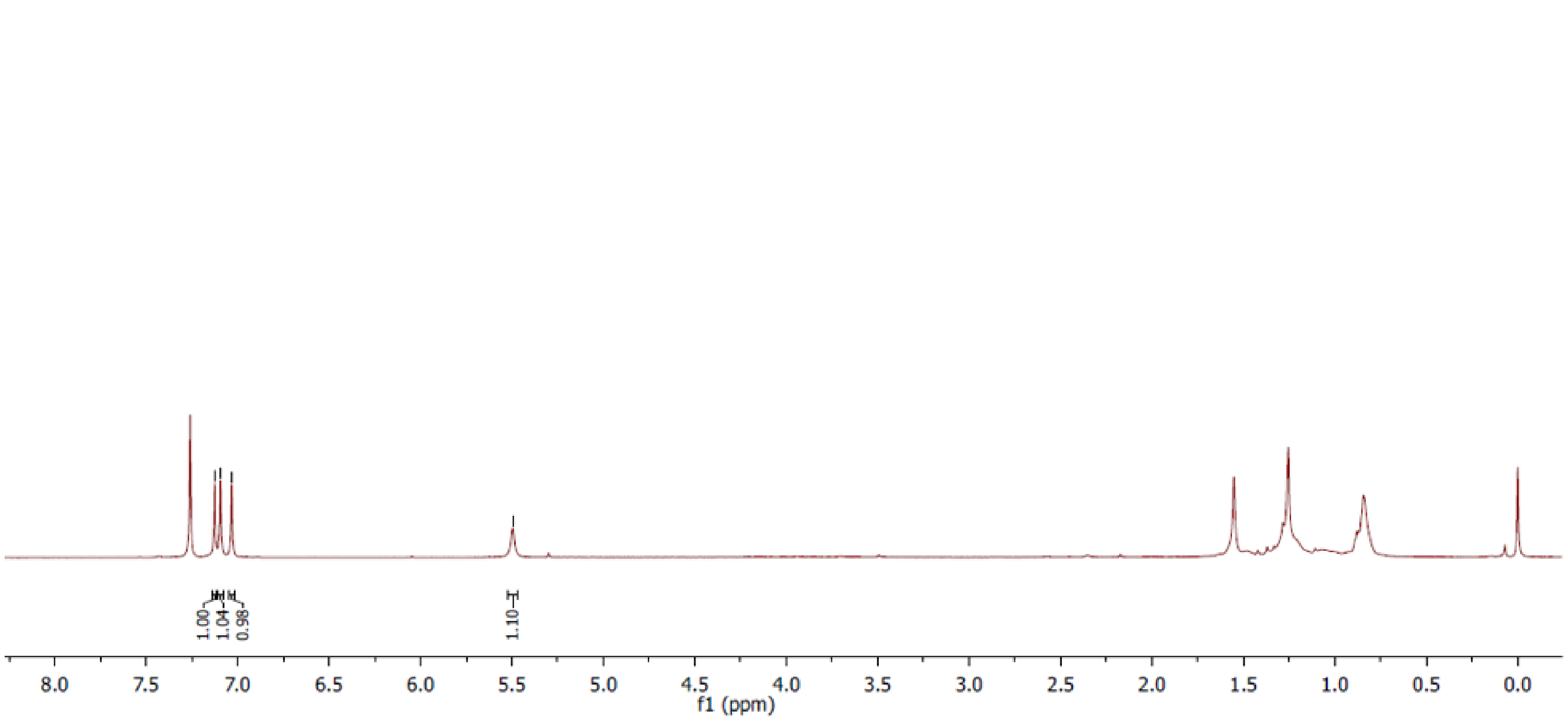
7.13
7.09
7.03
5.50
1.00
1.04
0.98
1.10
8.0
7.5
7.0
6.5
6.0
5.5
5.0
4.5
4.0
3.5
3.0
2.5
2.0
1.5
1.0
0.5
0.0
f1 (ppm)

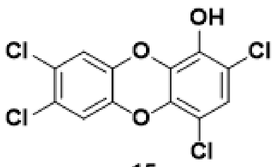
OH
Cl
Cl
Cl
Cl
O
O

15

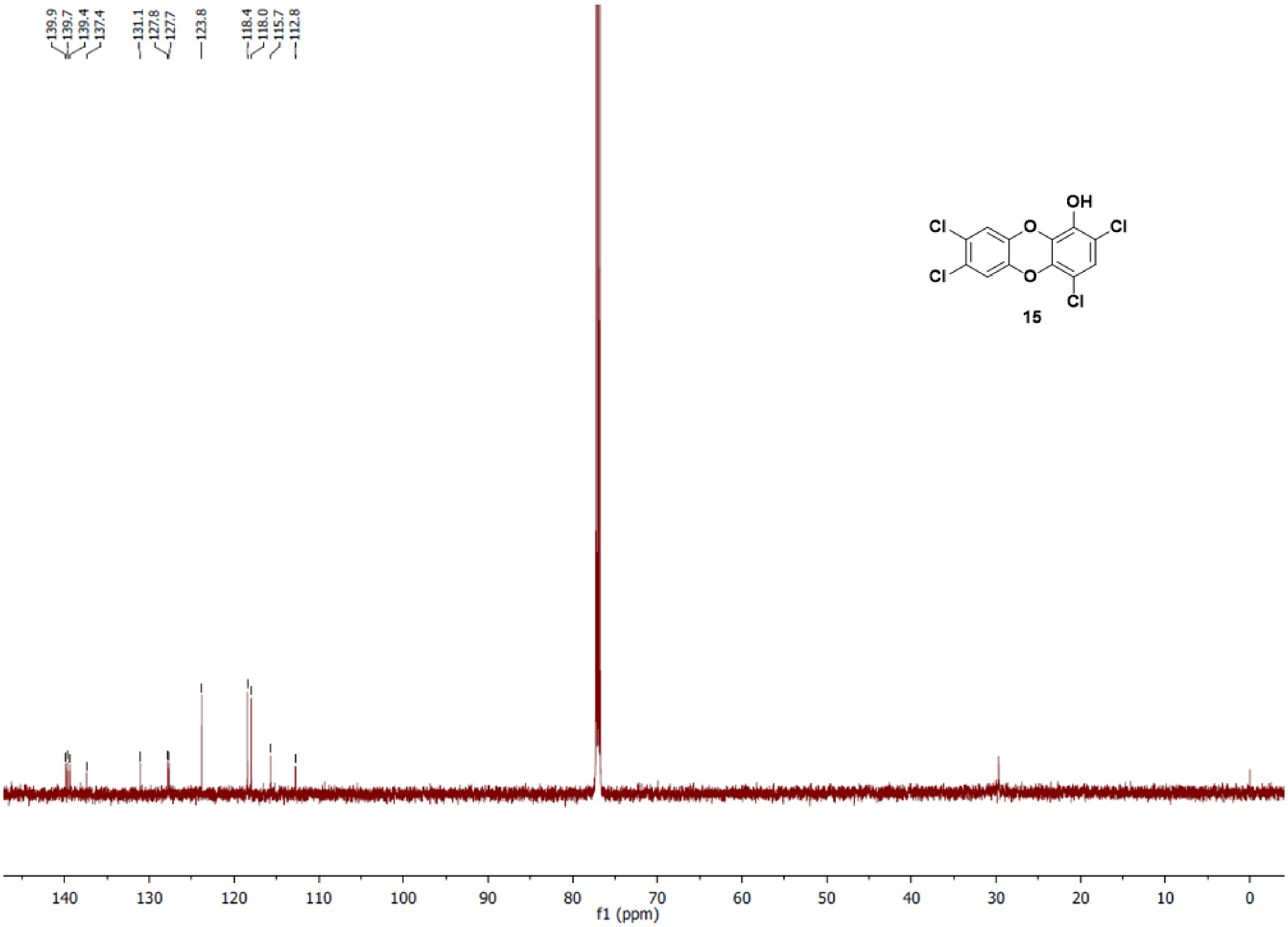
139.9
139.7
139.4
137.4
131.1
127.8
127.7
123.8
118.4
118.0
115.7
112.8
OH
Cl
Cl
Cl
Cl
O
O
15
140
130
120
110
100
90
80
70
60
50
40
30
20
10
0
f1 (ppm)

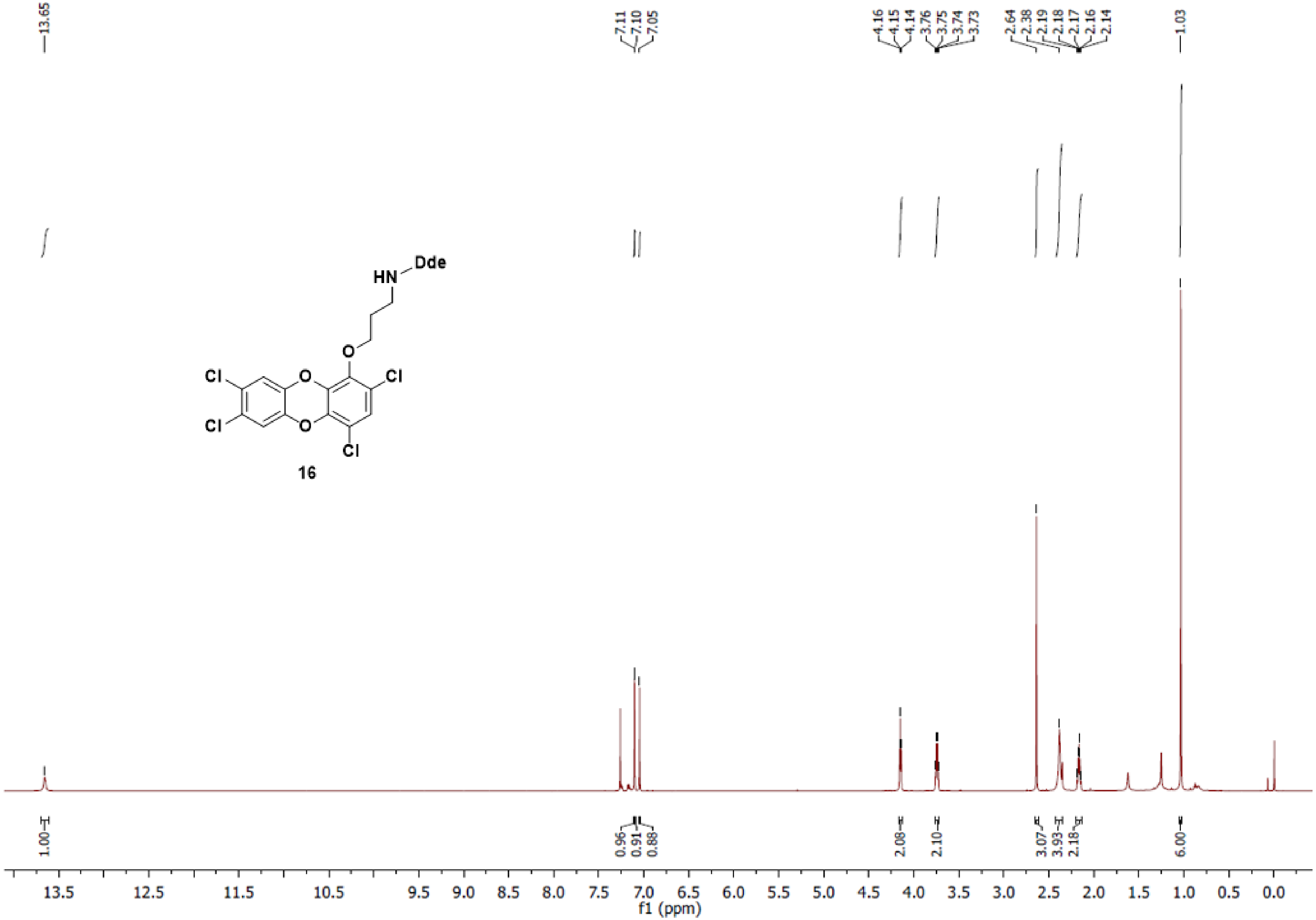
13.65
7.11
7.10
7.05
4.16
4.15
4.14
3.76
3.75
3.74
3.73
2.64
2.38
2.19
2.18
2.17
2.16
2.14
1.03
HN
Dde
O
Cl
Cl
Cl
Cl
16
1.00
0.96
0.91
0.88
2.08
2.10
3.07
3.93
2.18
6.00
13.5
12.5
11.5
10.5
9.5
9.0
8.5
8.0
7.5
7.0
6.5
6.0
5.5
5.0
4.5
4.0
3.5
3.0
2.5
2.0
1.5
1.0
0.5
0.0
f1 (ppm)

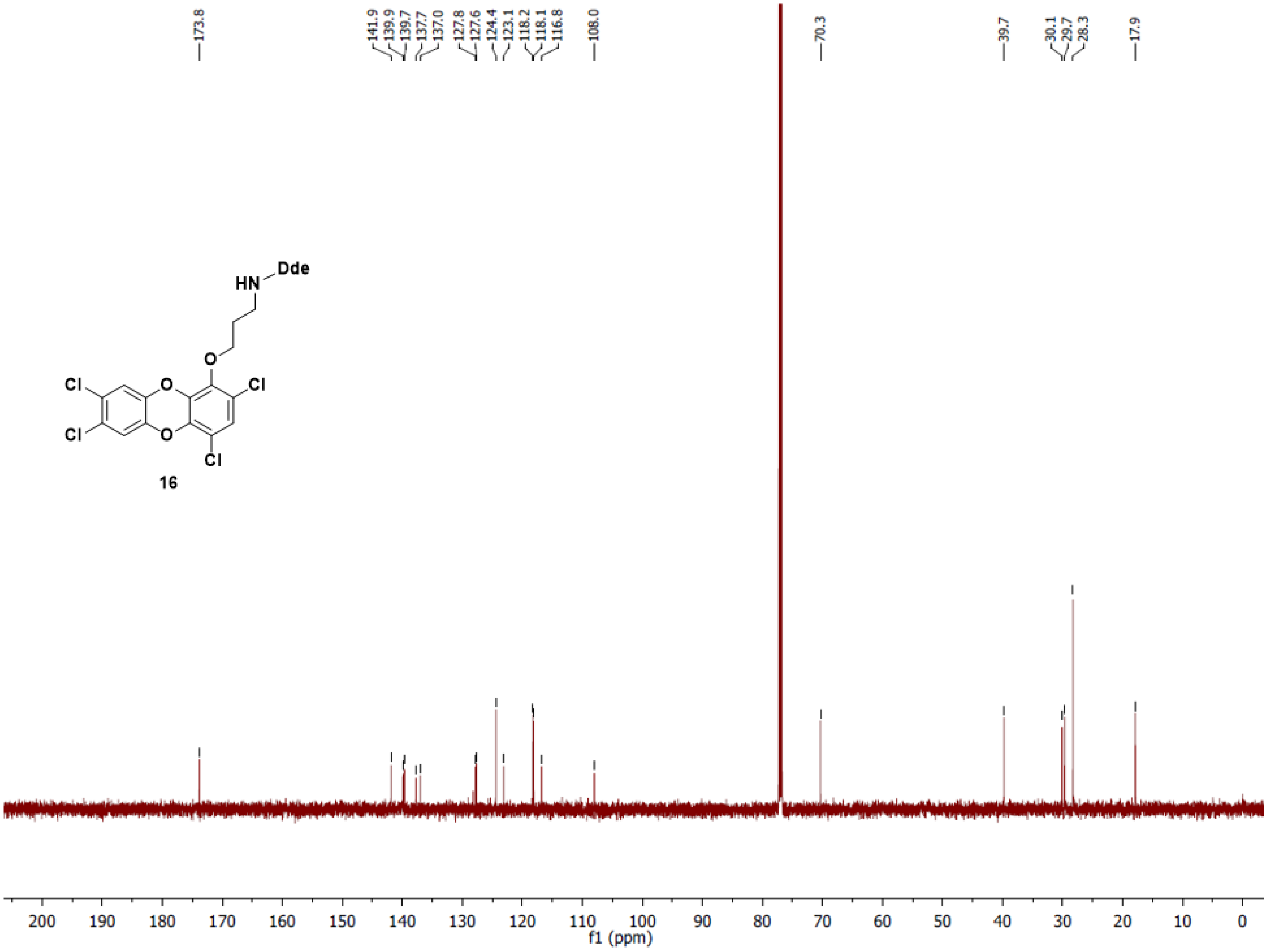

173.8
141.9
139.9
139.7
137.7
137.0
127.8
127.6
124.4
123.1
118.2
118.1
116.8
108.0
70.3
39.7
30.1
29.7
28.3
17.9
HN
Dde
O
Cl
O
Cl
Cl
O
Cl
200 190 180 170 160 150 140 130 120 110 100 90 80 70 60 50 40 30 20 10 0
f1 (ppm)

16

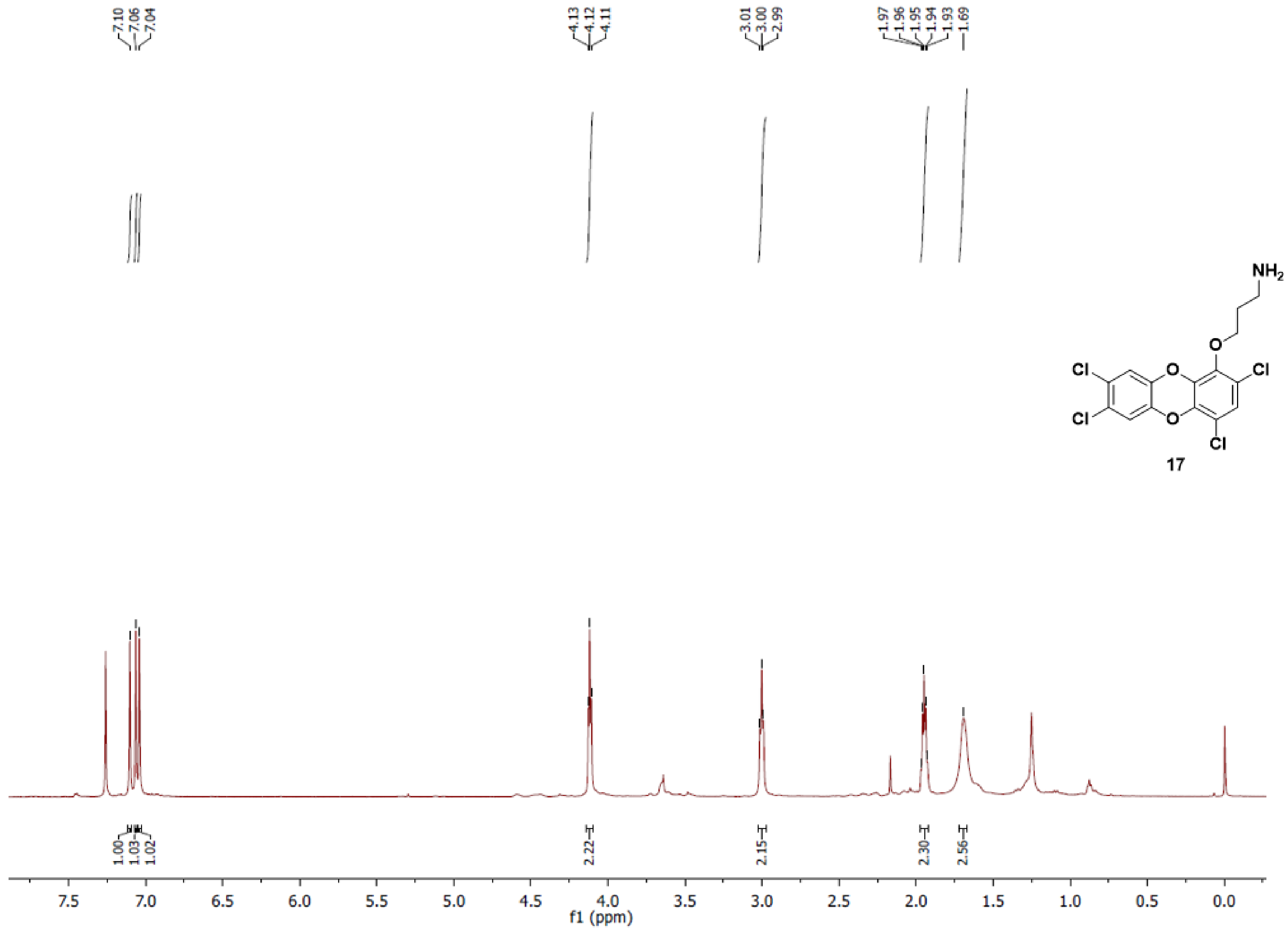

17

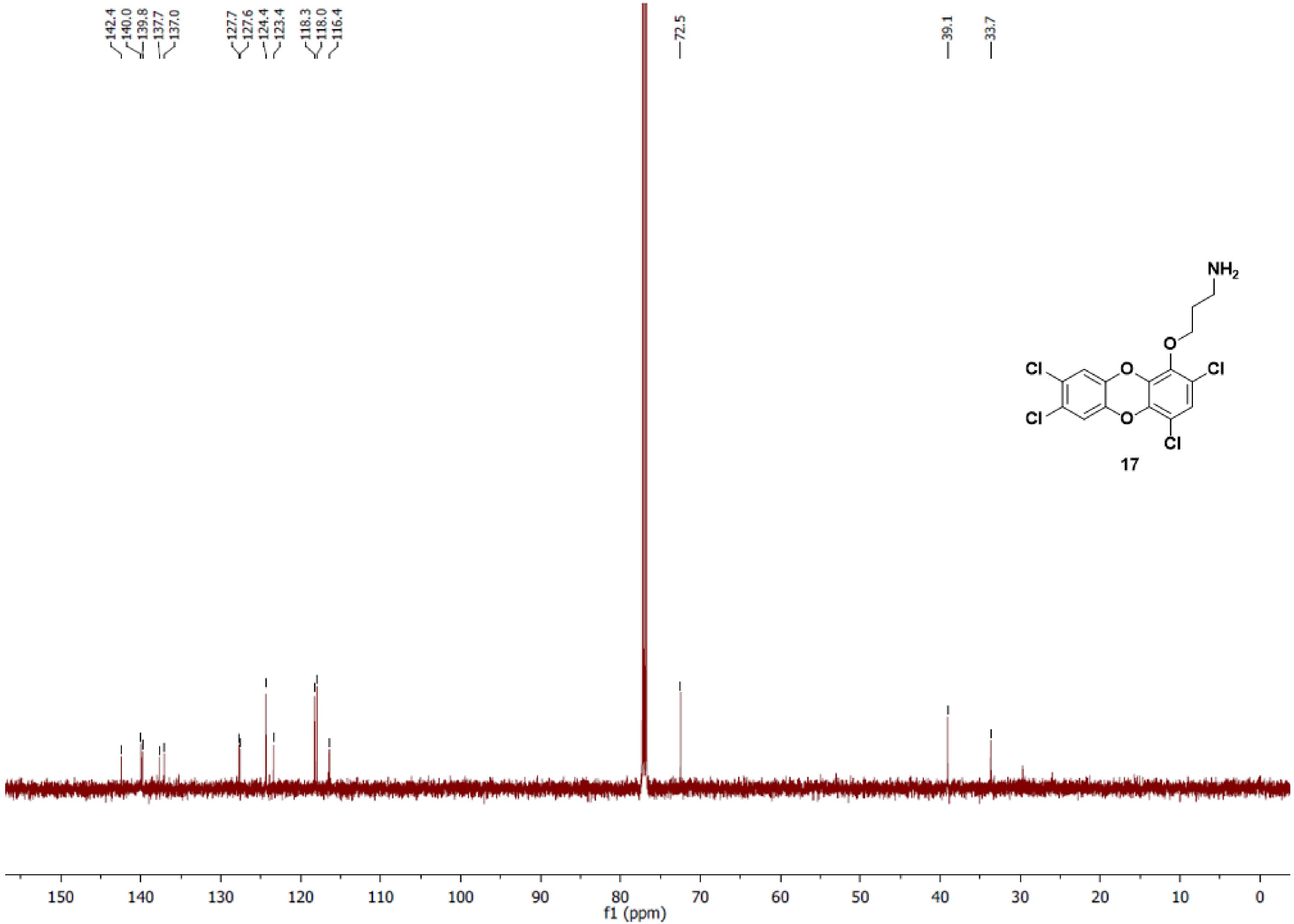

142.4
140.0
139.8
137.7
137.0
127.7
127.6
124.4
123.4
118.3
118.0
116.4
72.5
39.1
33.7
NH2
O
Cl
Cl
Cl
Cl
O
O
150
140
130
120
110
100
90
80
70
60
50
40
30
20
10
0
f1 (ppm)

17

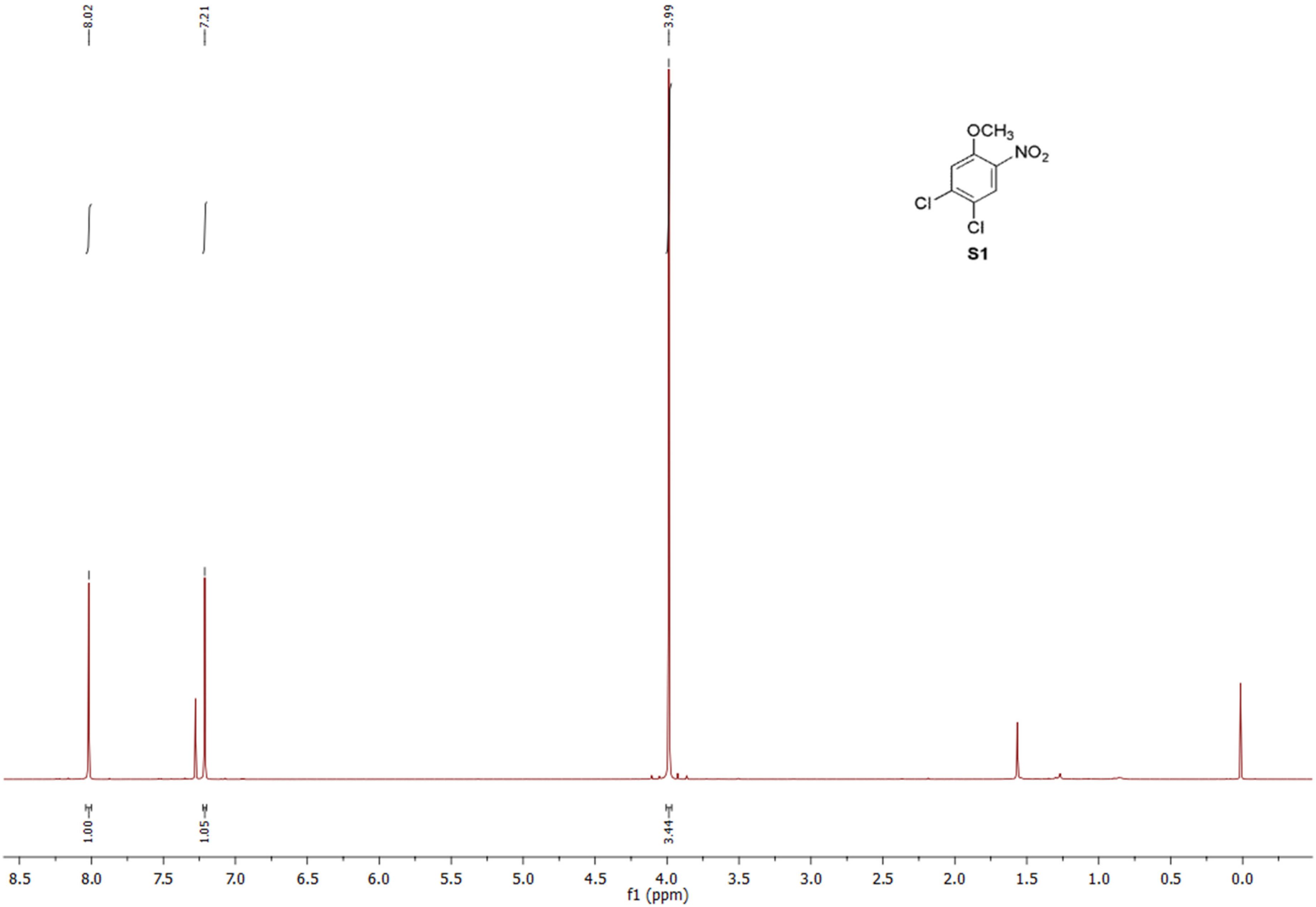
8.02
7.21
3.99
OCH3
NO2
Cl
Cl
S1
1.00
1.05
3.44
8.5 8.0 7.5 7.0 6.5 6.0 5.5 5.0 4.5 4.0 3.5 3.0 2.5 2.0 1.5 1.0 0.5 0.0
f1 (ppm)

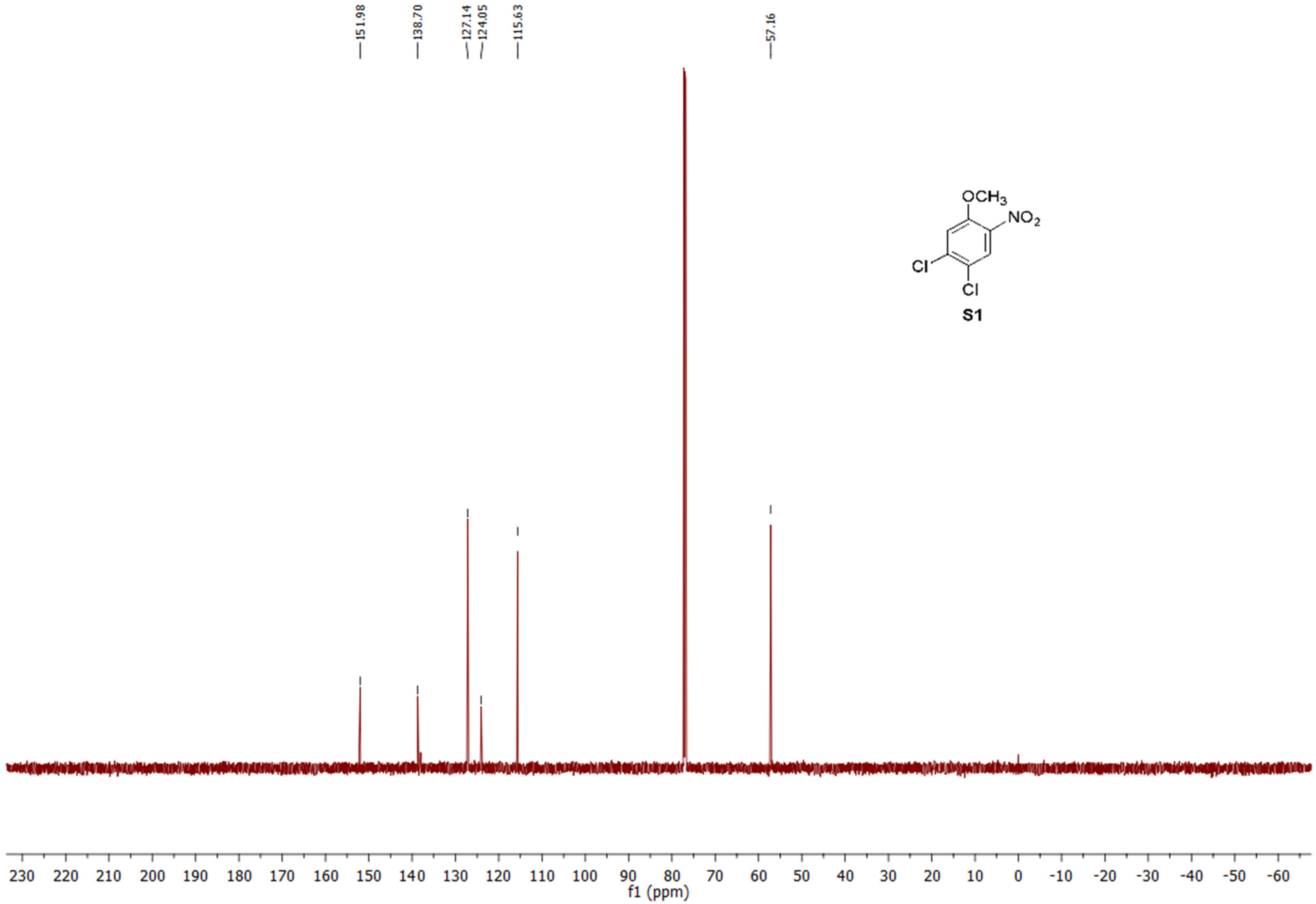

151.98
138.70
127.14
124.05
115.63
57.16
OCH3
NO2
Cl
Cl
S1
230 220 210 200 190 180 170 160 150 140 130 120 110 100 90 80 70 60 50 40 30 20 10 0 -10 -20 -30 -40 -50 -60
f1 (ppm)

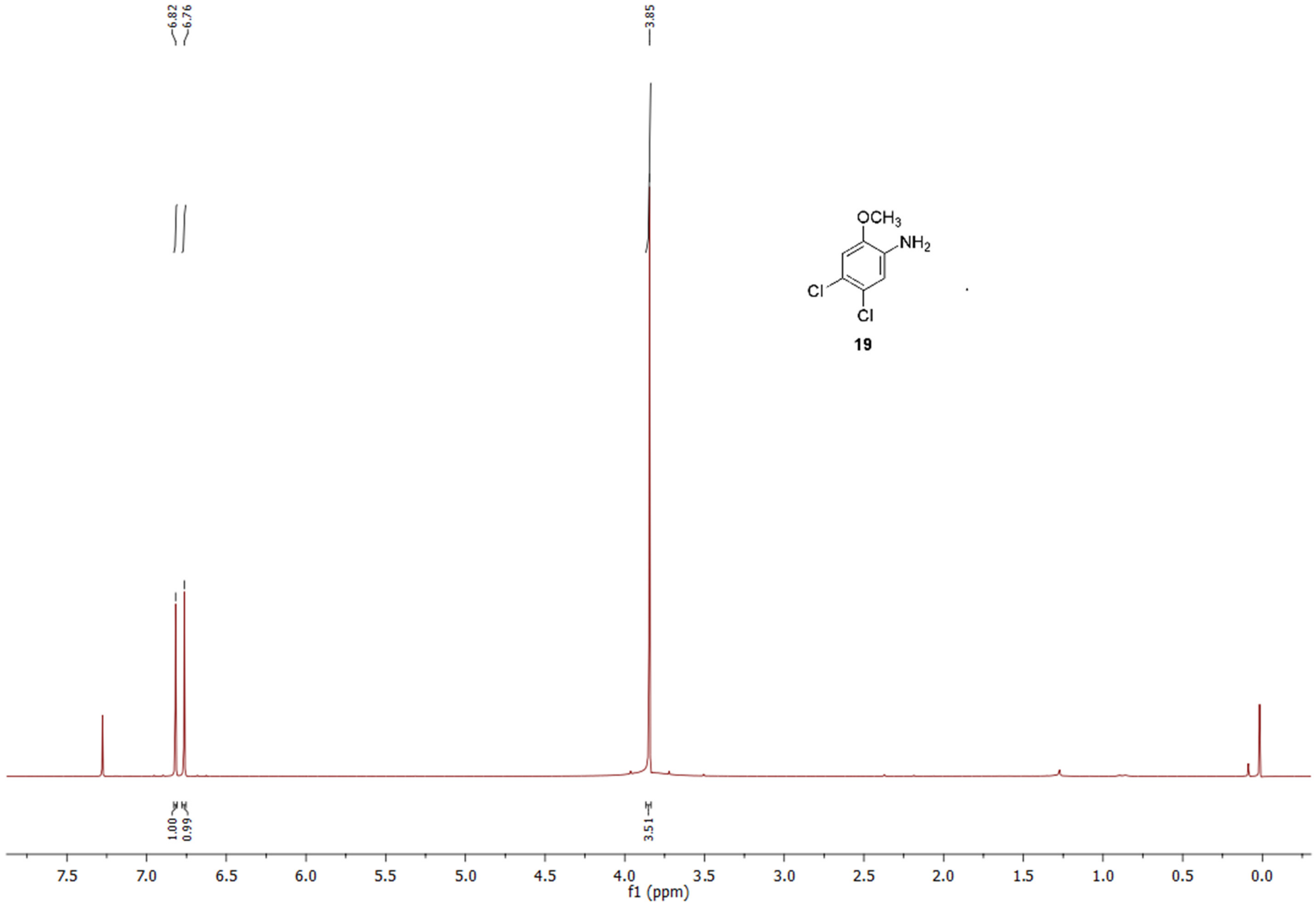

6.82
6.76
3.85
OCH3
NH2
Cl
Cl
19
1.00
0.99
3.51
7.5
7.0
6.5
6.0
5.5
5.0
4.5
4.0
3.5
3.0
2.5
2.0
1.5
1.0
0.5
0.0
f1 (ppm)

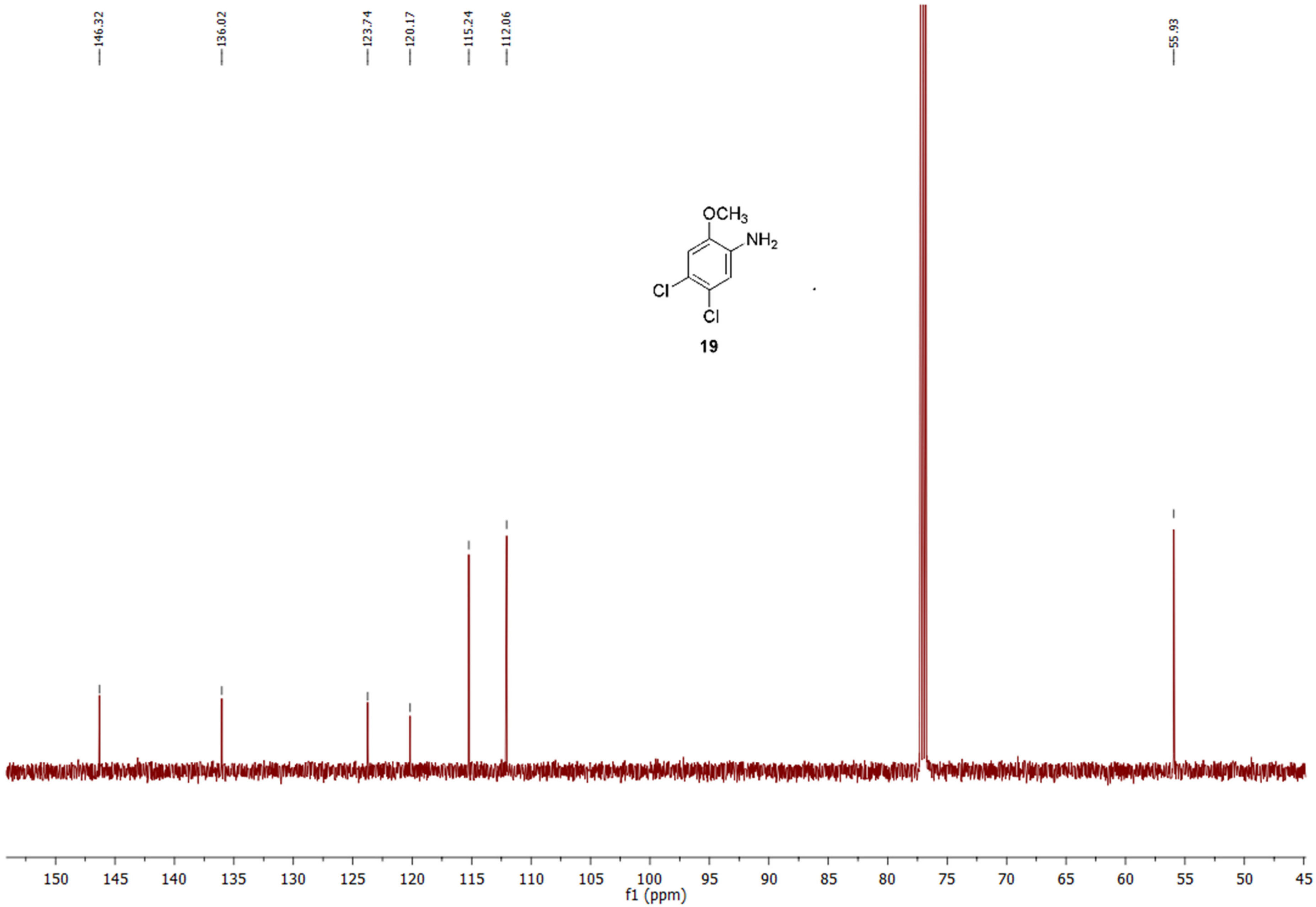
146.32
136.02
123.74
120.17
115.24
112.06
55.93
OCH3
NH2
Cl
Cl
19
150
145
140
135
130
125
120
115
110
105
100
95
90
85
80
75
70
65
60
55
50
45
f1 (ppm)

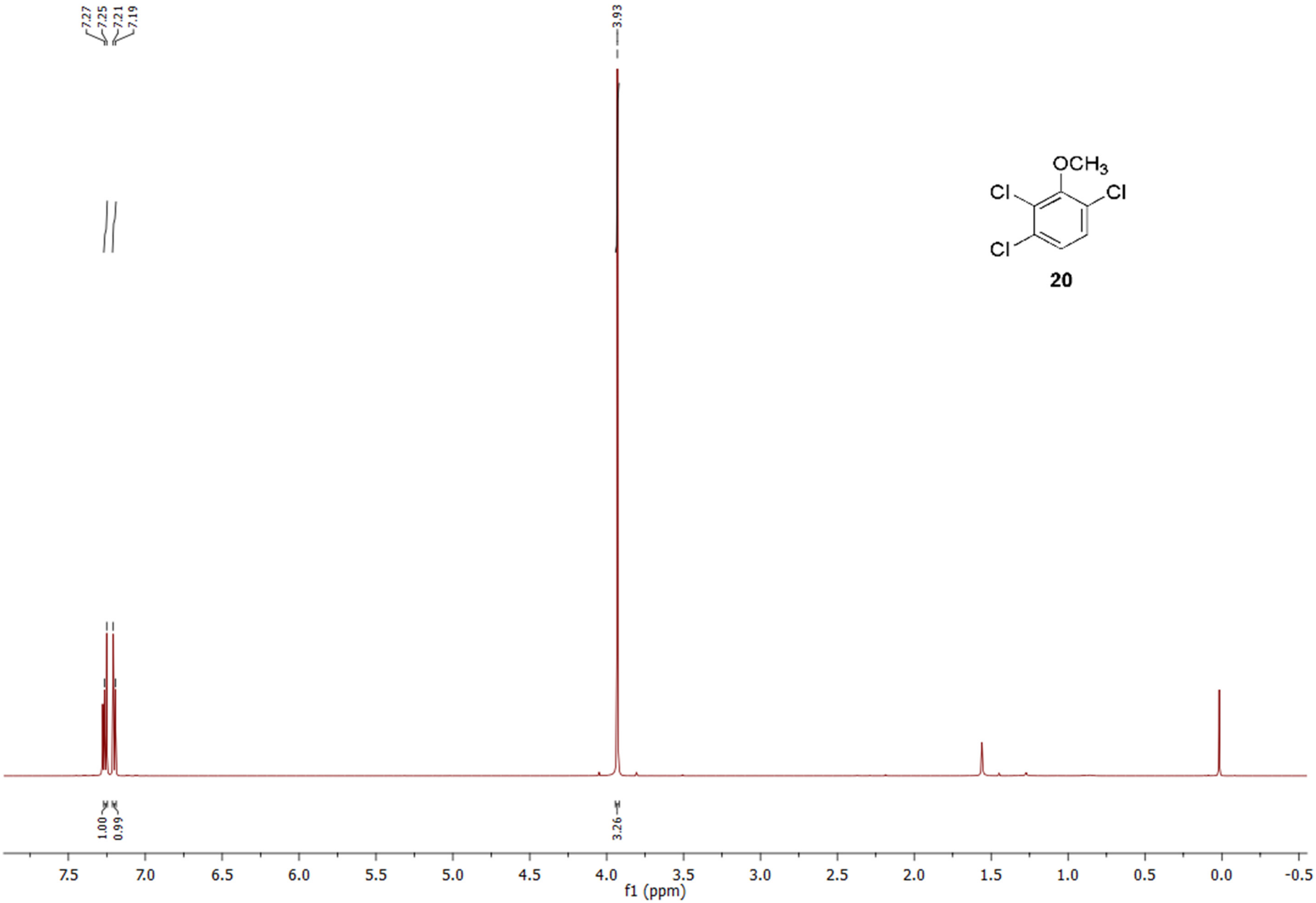
7.27
7.25
7.21
7.19
3.93
OCH3
Cl
Cl
Cl
20
1.00
0.99
3.26
7.5 7.0 6.5 6.0 5.5 5.0 4.5 4.0 3.5 3.0 2.5 2.0 1.5 1.0 0.5 0.0 -0.5
f1 (ppm)

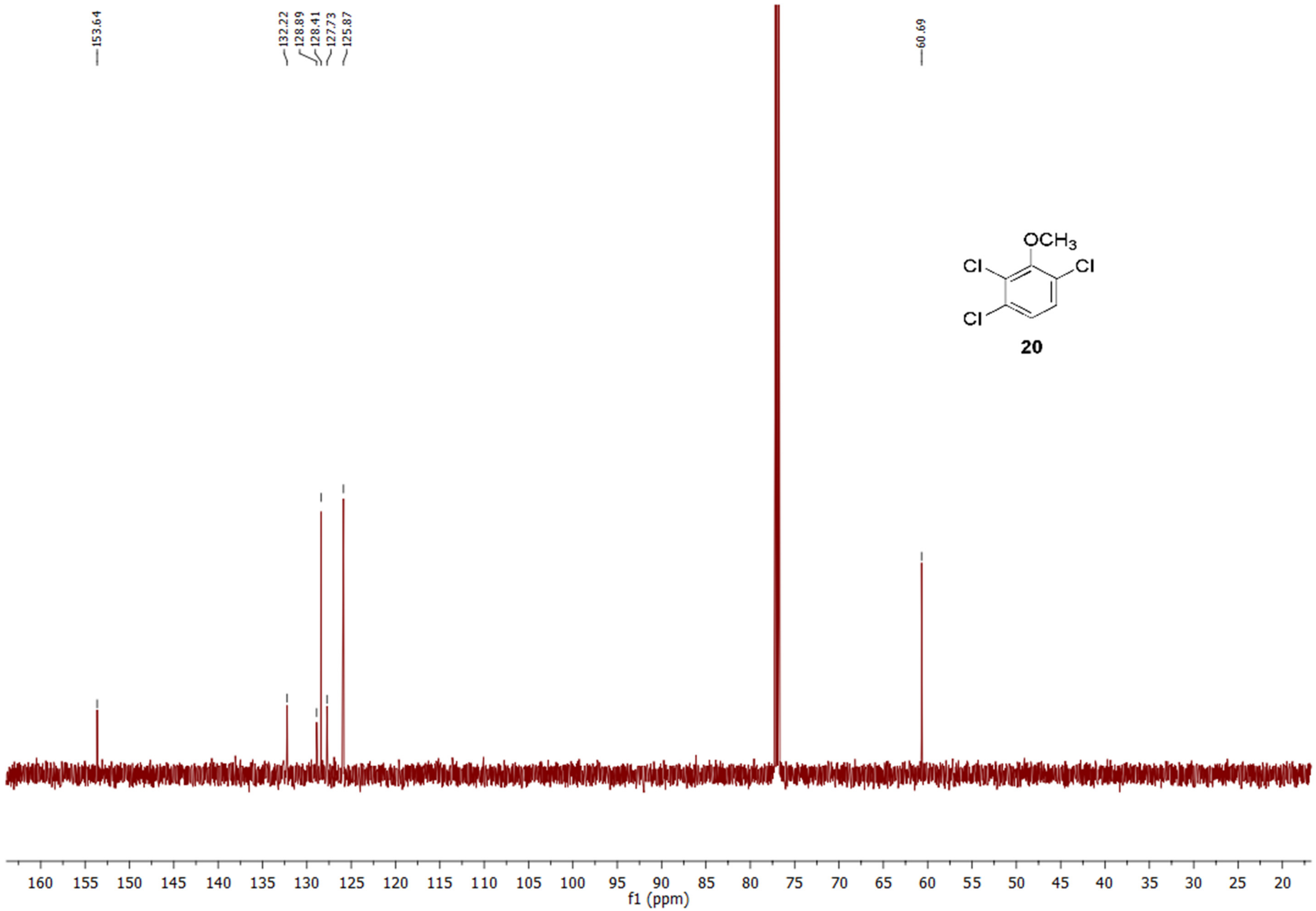
153.64
132.22
128.89
128.41
127.73
125.87
60.69
OCH3
Cl
Cl
Cl
20
160 155 150 145 140 135 130 125 120 115 110 105 100 95 90 85 80 75 70 65 60 55 50 45 40 35 30 25 20
f1 (ppm)

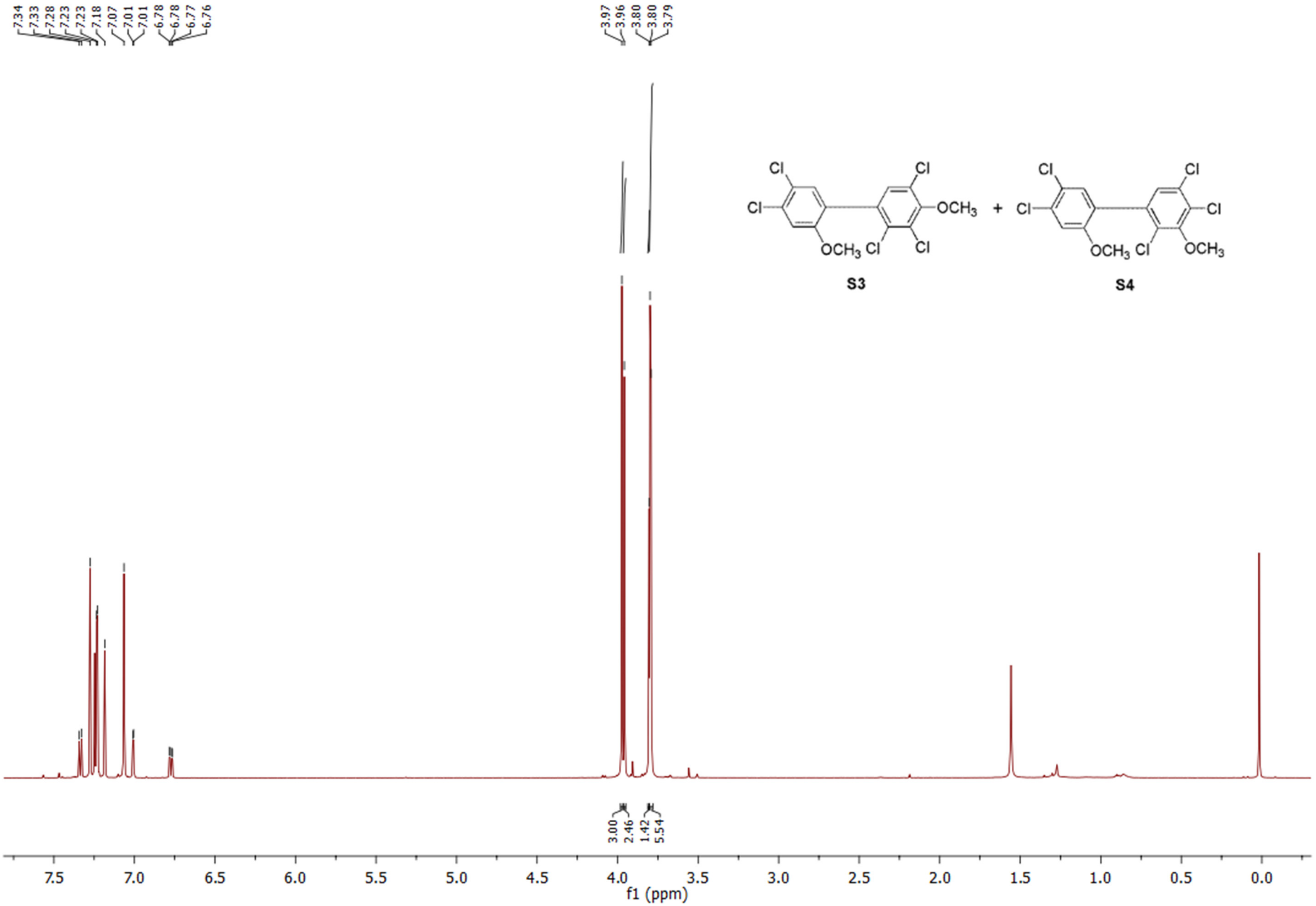
7.34
7.33
7.28
7.23
7.23
7.18
7.07
7.01
7.01
6.78
6.78
6.77
6.76
3.97
3.96
3.80
3.80
3.79
Cl
Cl
OCH3
Cl
Cl
Cl
OCH3
+
Cl
Cl
OCH3
Cl
Cl
Cl
OCH3
S3
S4
3.00
2.46
1.42
5.54
7.5
7.0
6.5
6.0
5.5
5.0
4.5
4.0
3.5
3.0
2.5
2.0
1.5
1.0
0.5
0.0
f1 (ppm)

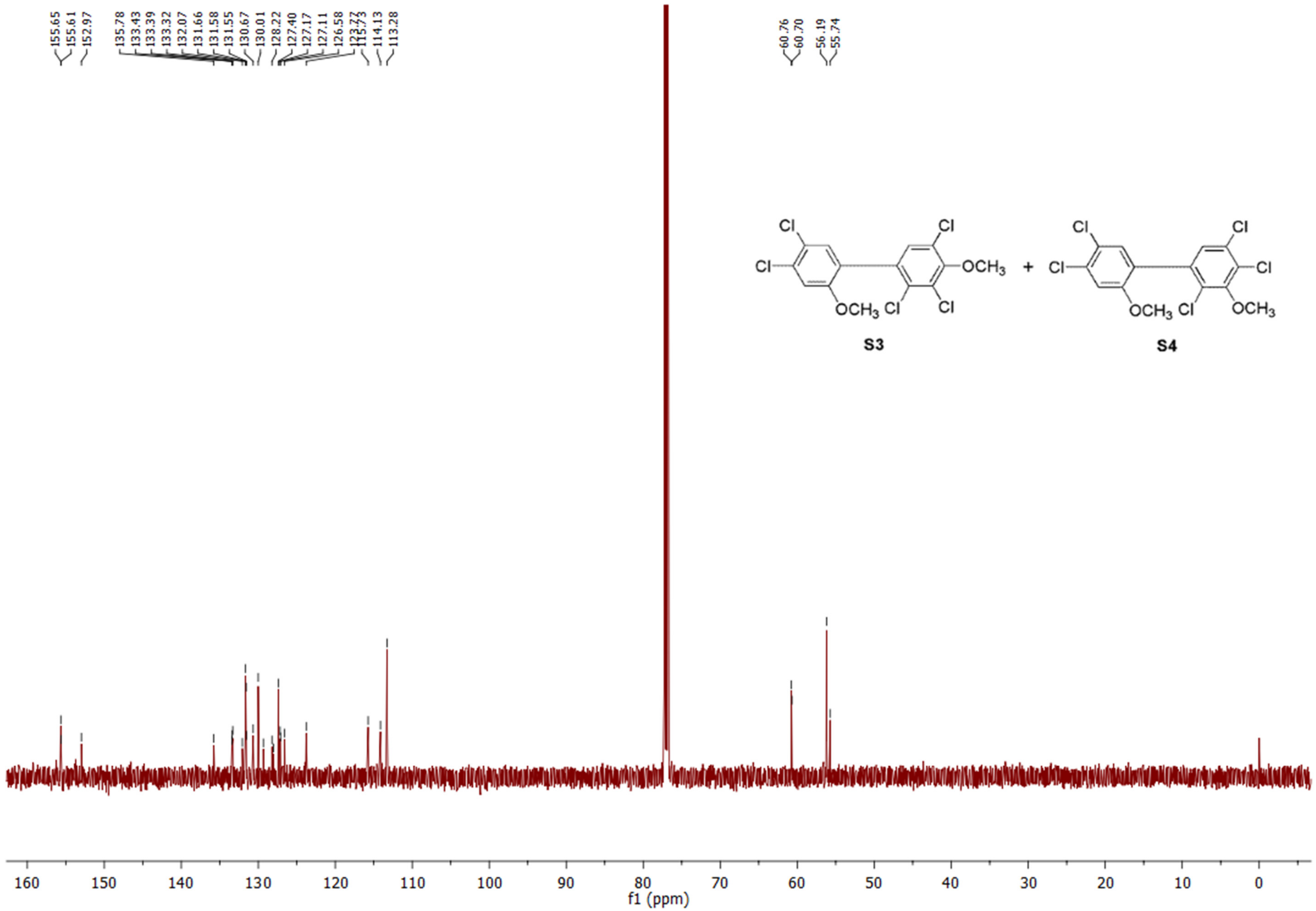
155.65
155.61
152.97
135.78
133.43
133.39
133.32
132.07
131.66
131.58
131.55
130.67
130.01
128.22
127.40
127.17
127.11
126.58
114.13
113.28
60.76
60.70
56.19
55.74
Cl
Cl
OCH3
Cl
OCH3
Cl
Cl
+
Cl
Cl
OCH3
Cl
Cl
Cl
OCH3
S3
S4
f1 (ppm)

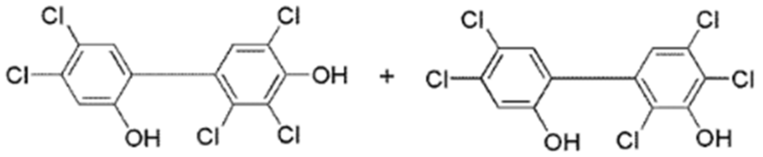

Cl
Cl
OH
Cl
Cl
Cl
OH
+

S5 **S6**

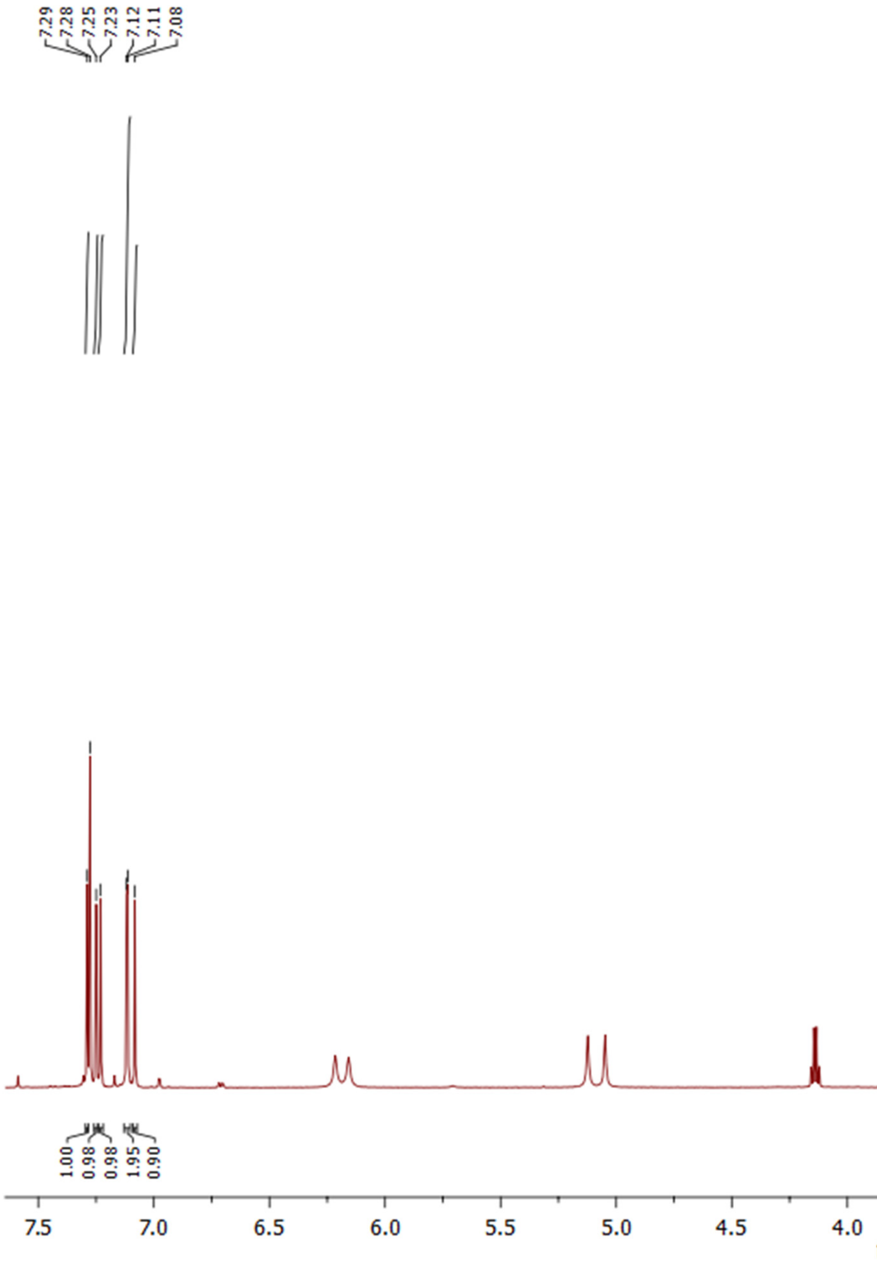

7.29
7.28
7.25
7.23
7.12
7.11
7.08
1.00
0.98
0.98
1.95
0.90
7.5 7.0 6.5 6.0 5.5 5.0 4.5 4.0 3.5 3.0 2.5 2.0 1.5 1.0 0.5 0.0
f1 (ppm)

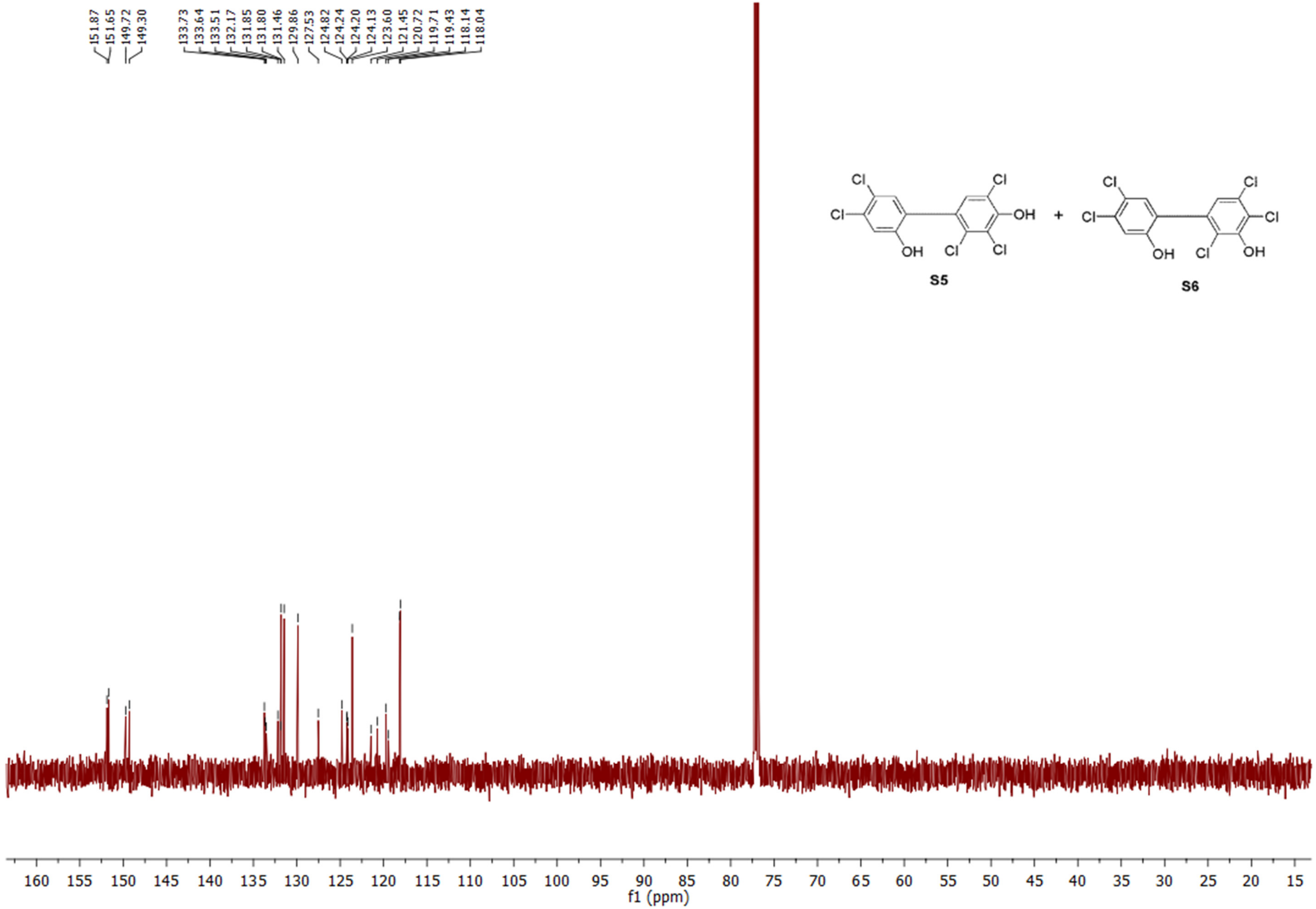
151.87
151.65
149.72
149.30
133.73
133.64
133.51
132.17
131.85
131.80
131.46
129.86
127.53
124.82
124.24
124.20
124.13
123.60
121.45
120.72
119.71
119.43
118.14
118.04
Cl
Cl
OH
Cl
Cl
Cl
OH
+
Cl
Cl
OH
Cl
Cl
Cl
OH
S5
S6
160 155 150 145 140 135 130 125 120 115 110 105 100 95 90 85 80 75 70 65 60 55 50 45 40 35 30 25 20 15
f1 (ppm)

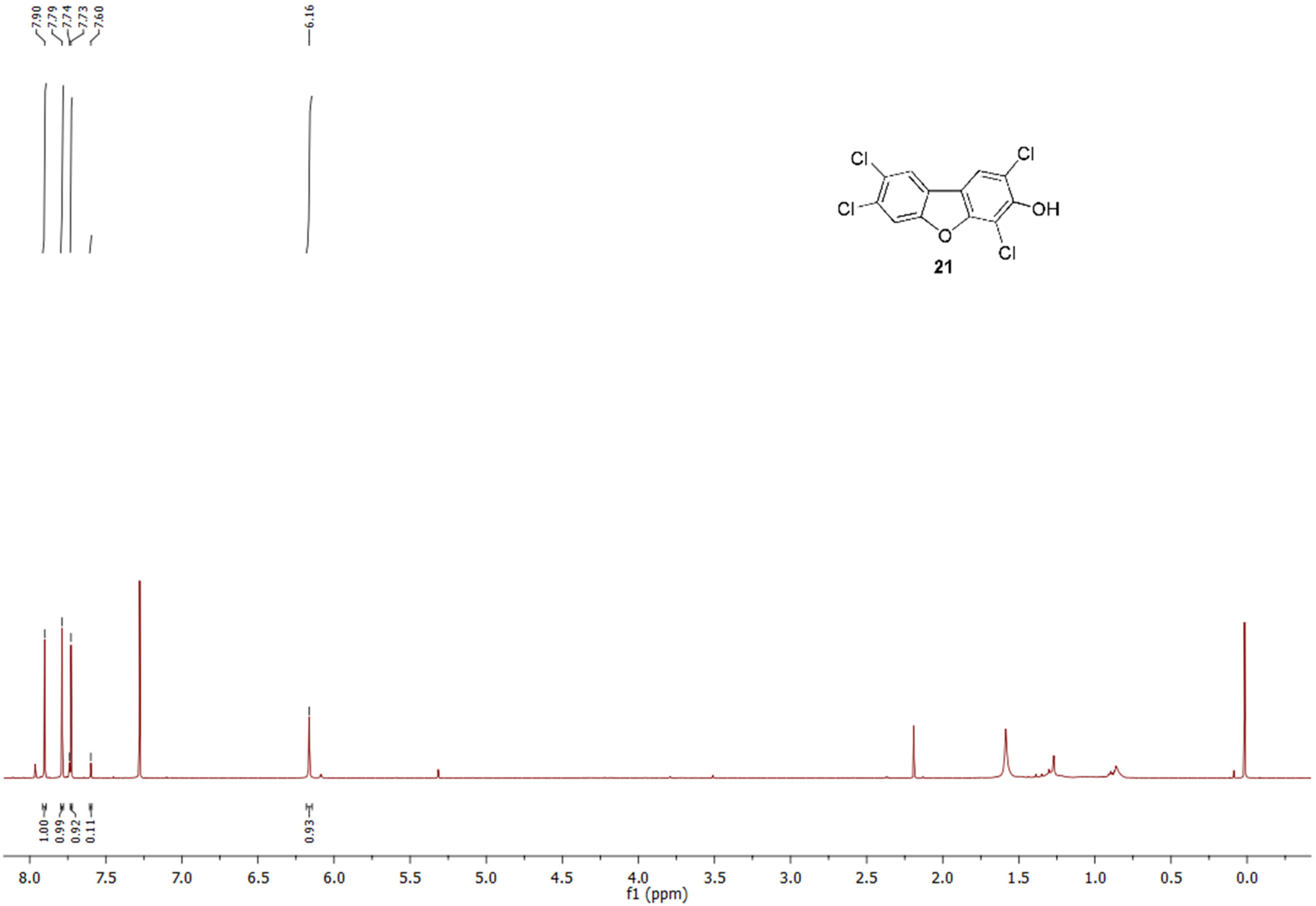
7.90
7.79
7.74
7.73
7.60
6.16
Cl
Cl
Cl
OH
O
Cl
21
1.00
0.99
0.92
0.11
0.93
8.0
7.5
7.0
6.5
6.0
5.5
5.0
4.5
4.0
3.5
3.0
2.5
2.0
1.5
1.0
0.5
0.0
f1 (ppm)

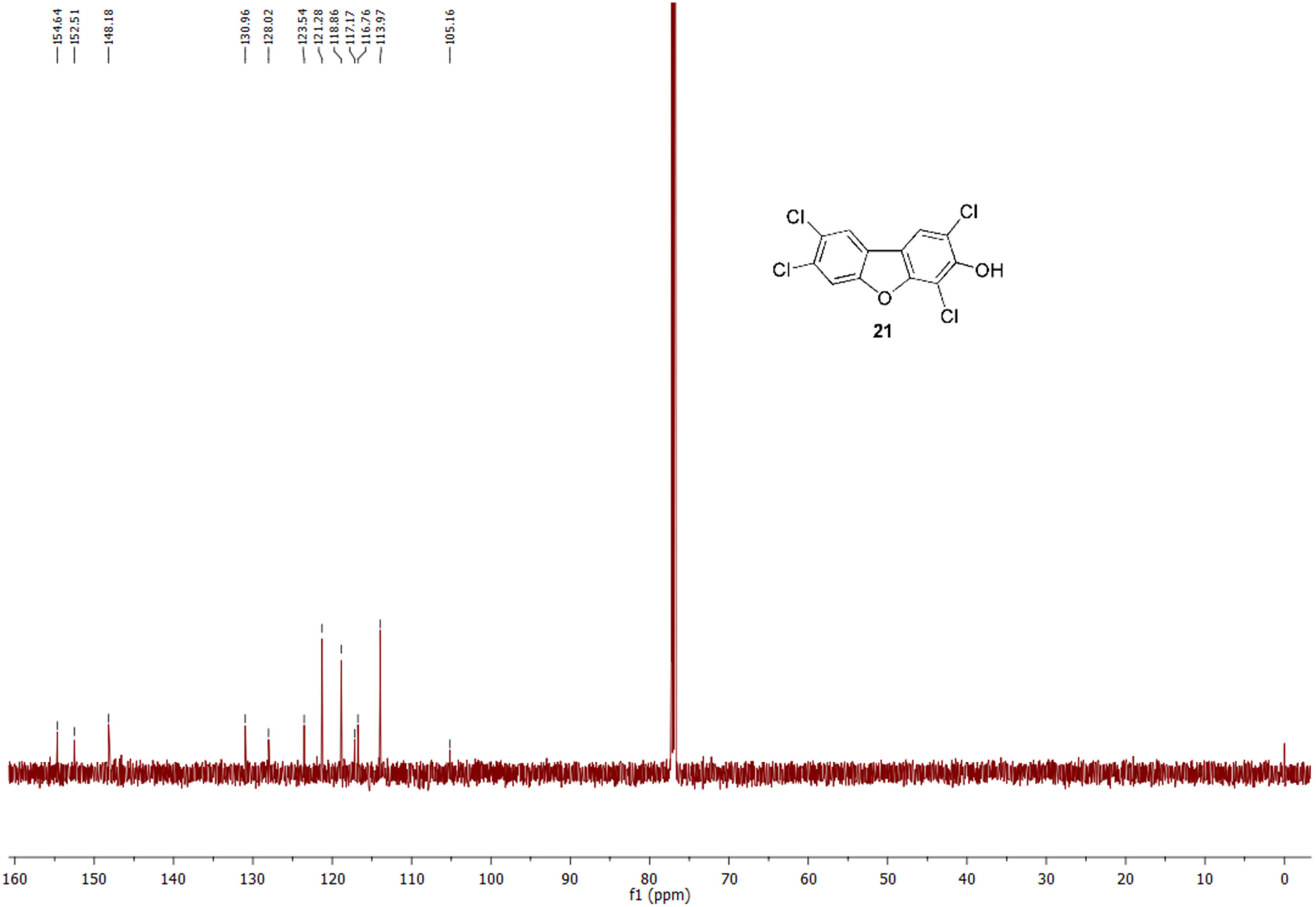
154.64
152.51
148.18
130.96
128.02
123.54
121.28
118.86
117.17
116.76
113.97
105.16
160
150
140
130
120
110
100
90
80
70
60
50
40
30
20
10
0
f1 (ppm)
Cl
Cl
Cl
OH
O
Cl

21

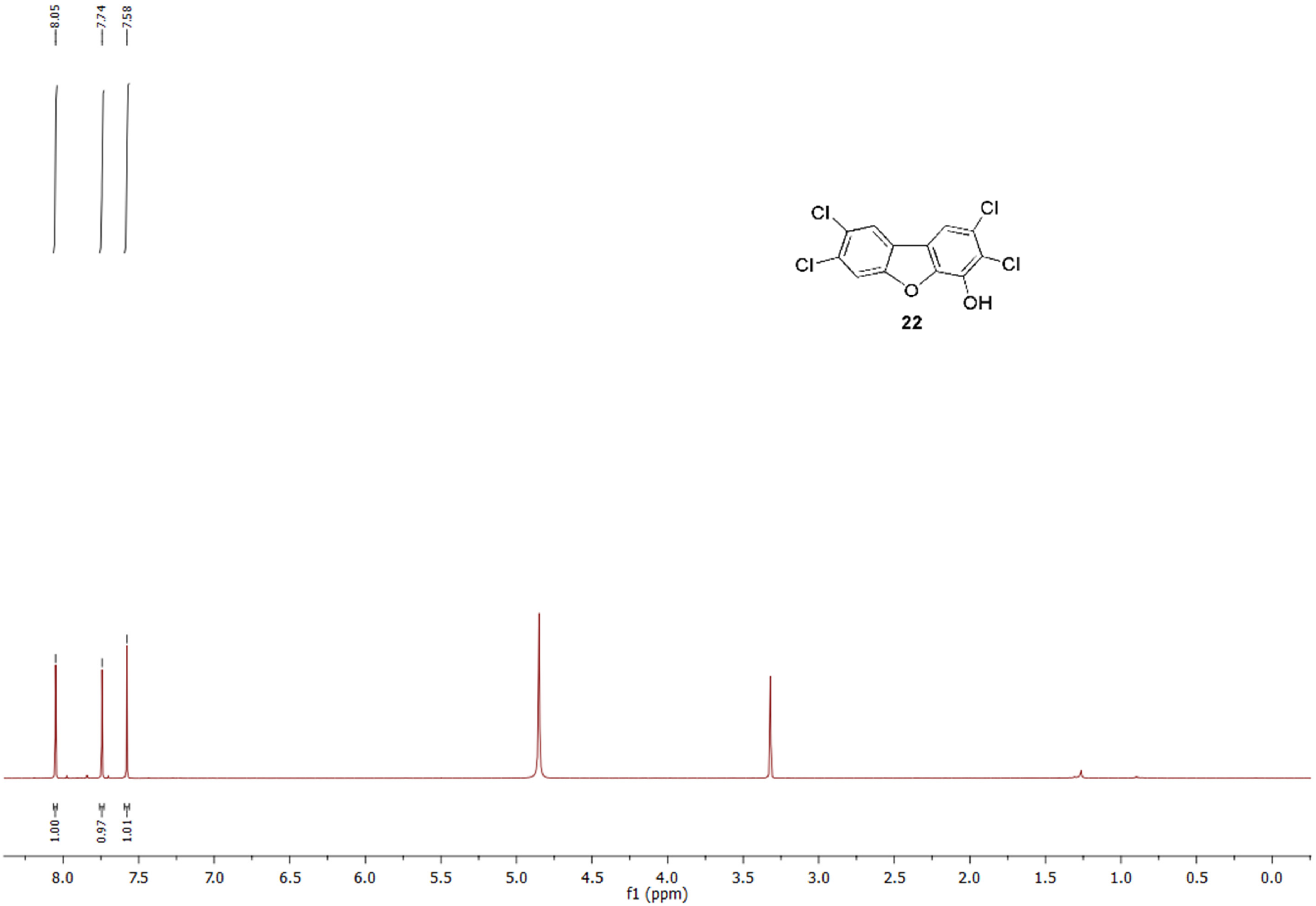
8.05
7.74
7.58
Cl
Cl
Cl
Cl
O
OH
1.00
0.97
1.01
8.0
7.5
7.0
6.5
6.0
5.5
5.0
4.5
4.0
3.5
3.0
2.5
2.0
1.5
1.0
0.5
0.0
f1 (ppm)

22

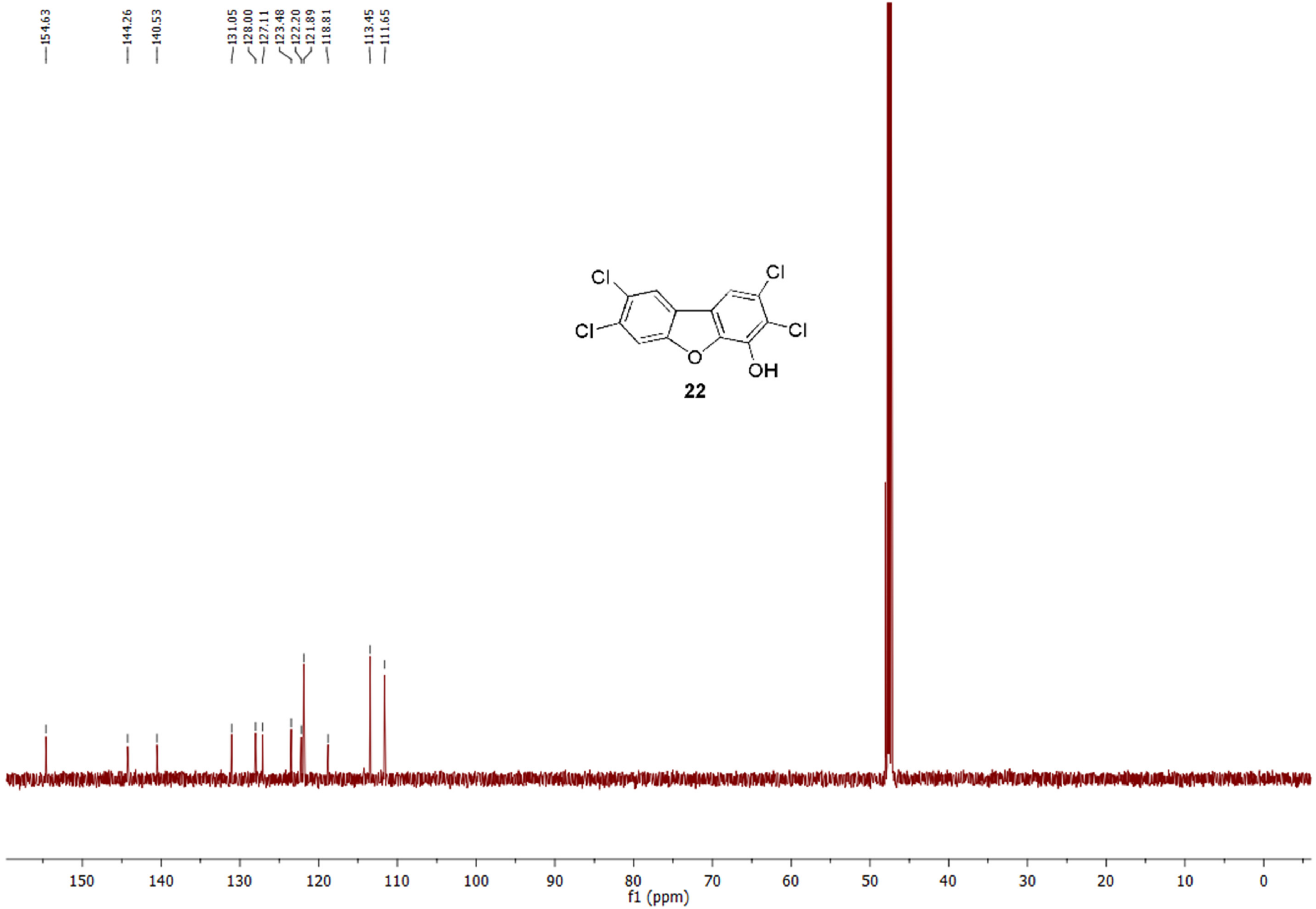
154.63
144.26
140.53
131.05
128.00
127.11
123.48
122.20
121.89
118.81
113.45
111.65
Cl
Cl
Cl
Cl
O
OH
22
150
140
130
120
110
100
90
80
70
60
50
40
30
20
10
0
f1 (ppm)

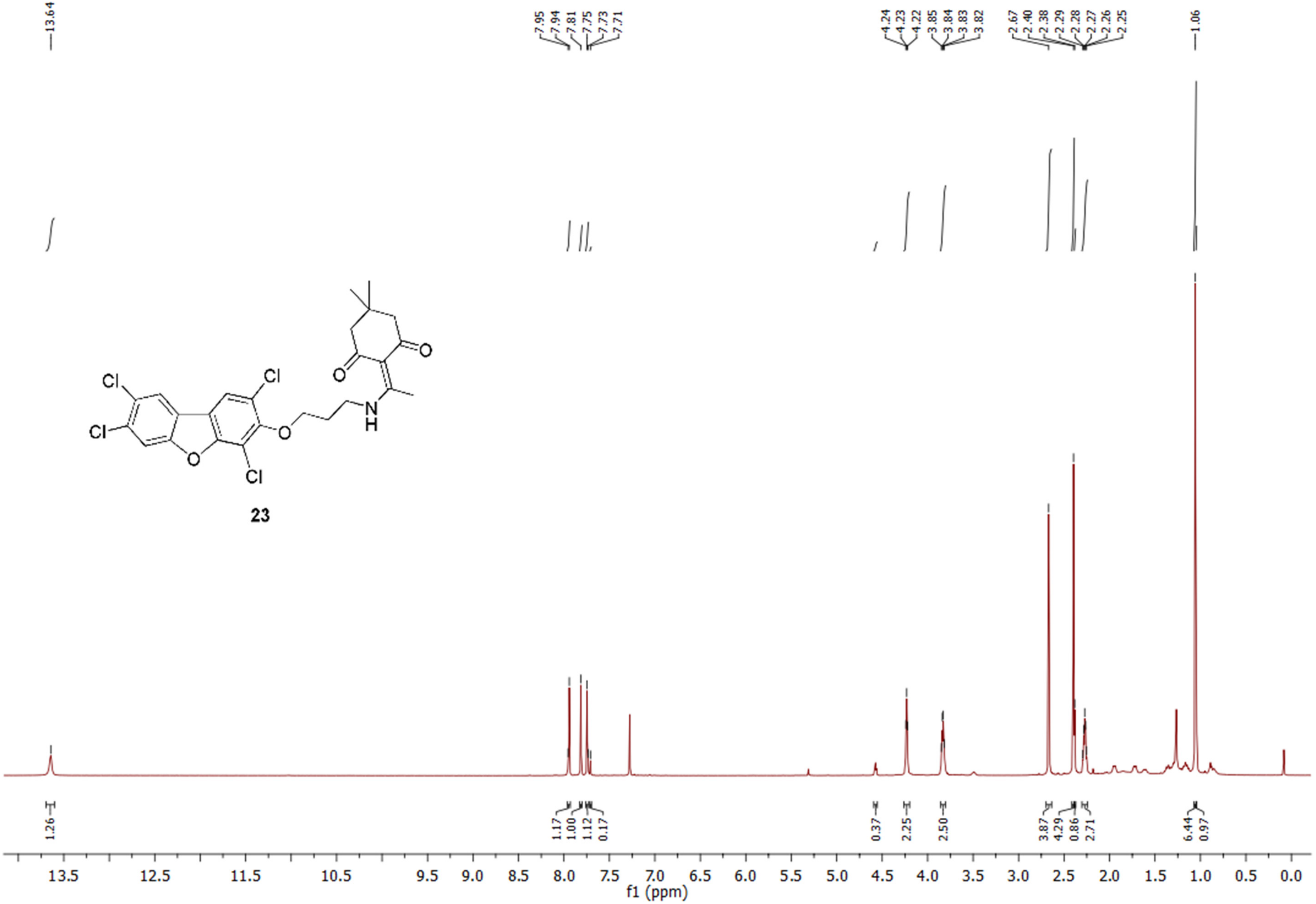
13.64
7.95
7.94
7.81
7.75
7.73
7.71
4.24
4.23
4.22
3.85
3.84
3.83
3.82
2.67
2.40
2.38
2.29
2.28
2.27
2.26
2.25
1.06
1.26
1.17
1.00
1.12
0.17
0.37
2.25
2.50
3.87
4.29
0.86
2.71
6.44
0.97
f1 (ppm)
Cl
O
NH
23

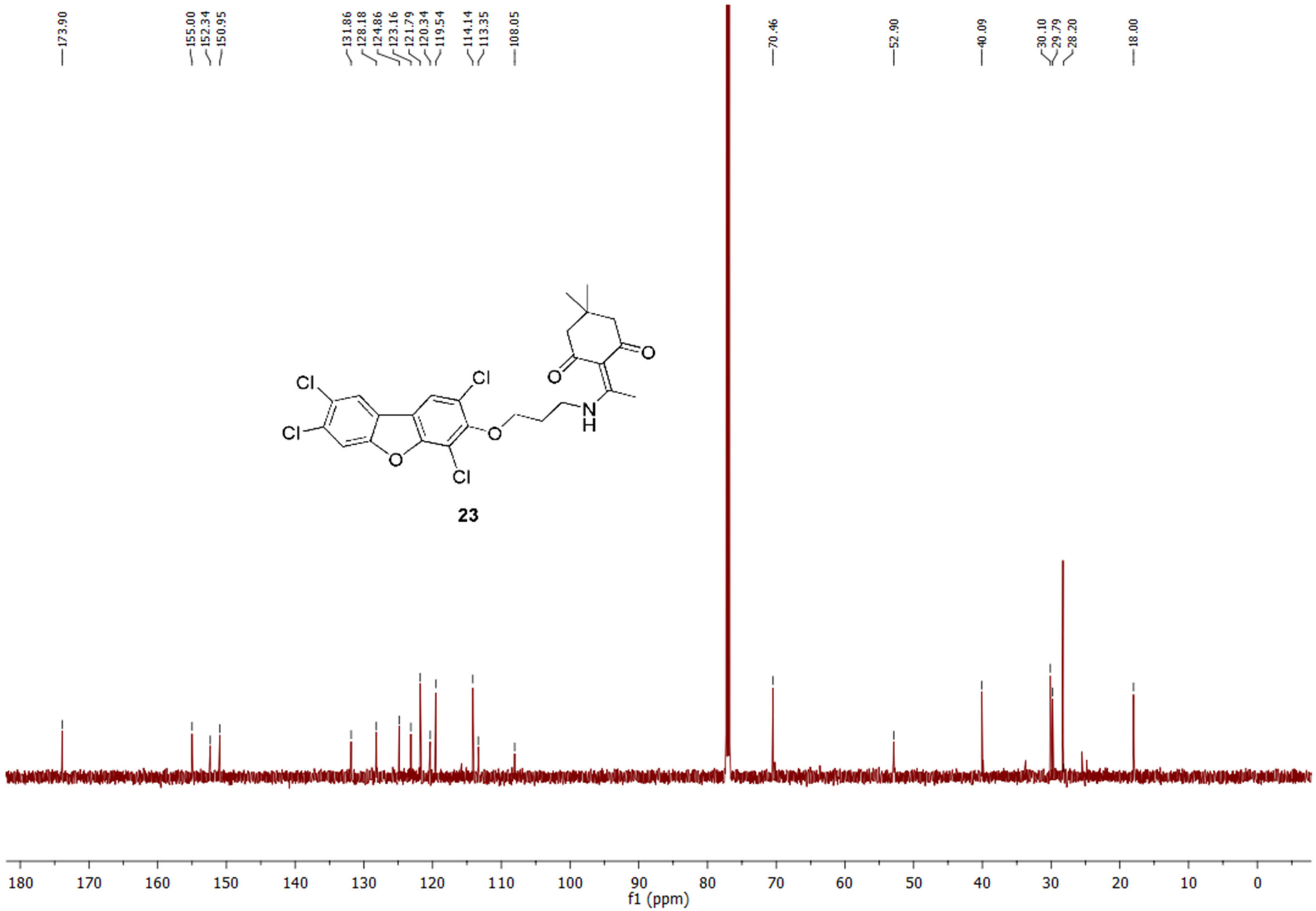
173.90
155.00
152.34
150.95
131.86
128.18
124.86
123.16
121.79
120.34
119.54
114.14
113.35
108.05
70.46
52.90
40.09
30.10
29.79
28.20
18.00
Cl
Cl
Cl
Cl
O
O
N
H
O
O
180
170
160
150
140
130
120
110
100
90
80
70
60
50
40
30
20
10
0
f1 (ppm)

23

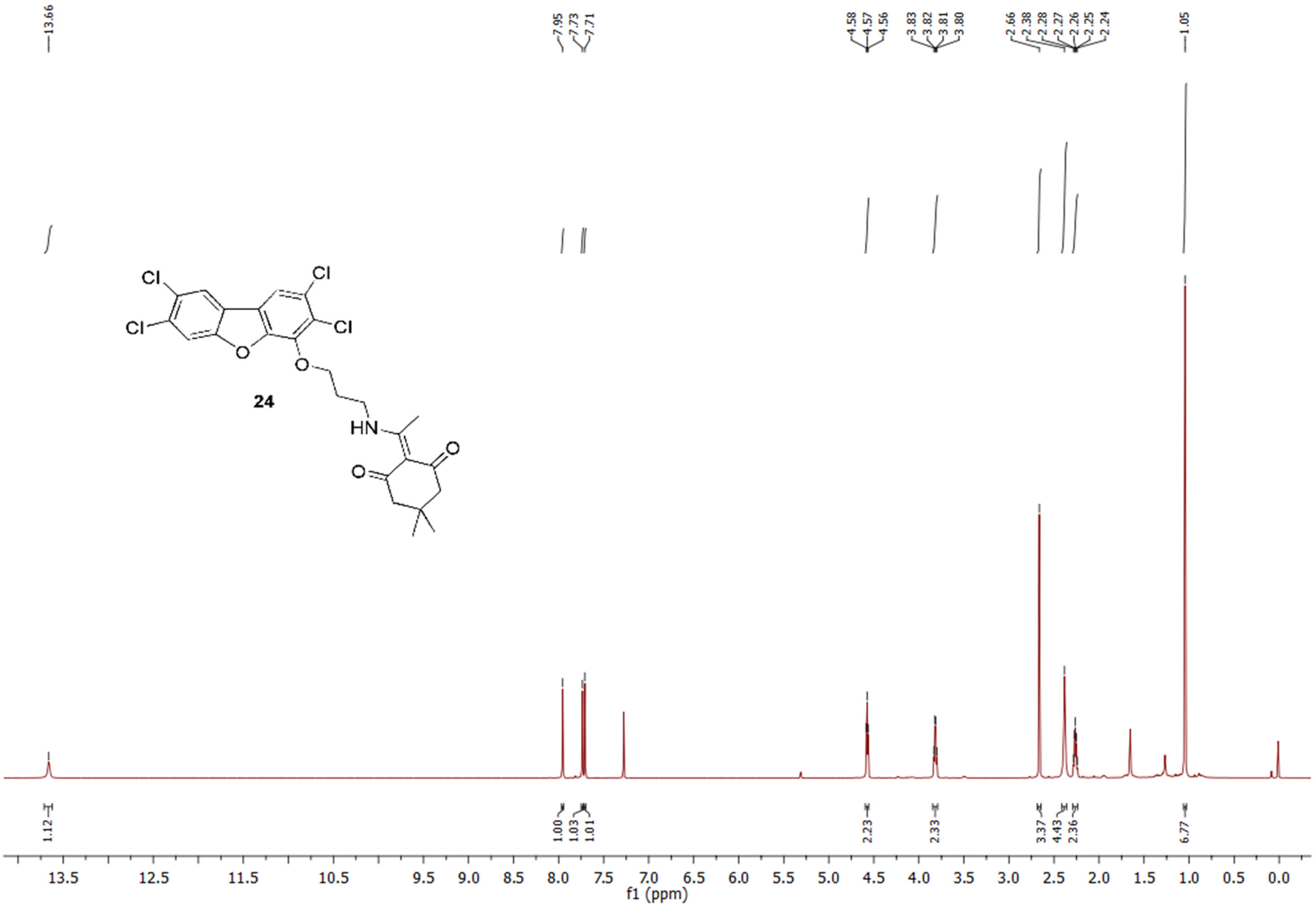
13.66
7.95
7.73
7.71
4.58
4.57
4.56
3.83
3.82
3.81
3.80
2.66
2.38
2.28
2.27
2.26
2.25
2.24
1.05
Cl
Cl
Cl
Cl
O
O
24
HN
O
O
1.12
1.00
1.03
1.01
2.23
2.33
3.37
4.43
2.36
6.77
13.5
12.5
11.5
10.5
9.5
9.0
8.5
8.0
7.5
7.0
6.5
6.0
5.5
5.0
4.5
4.0
3.5
3.0
2.5
2.0
1.5
1.0
0.5
0.0
f1 (ppm)

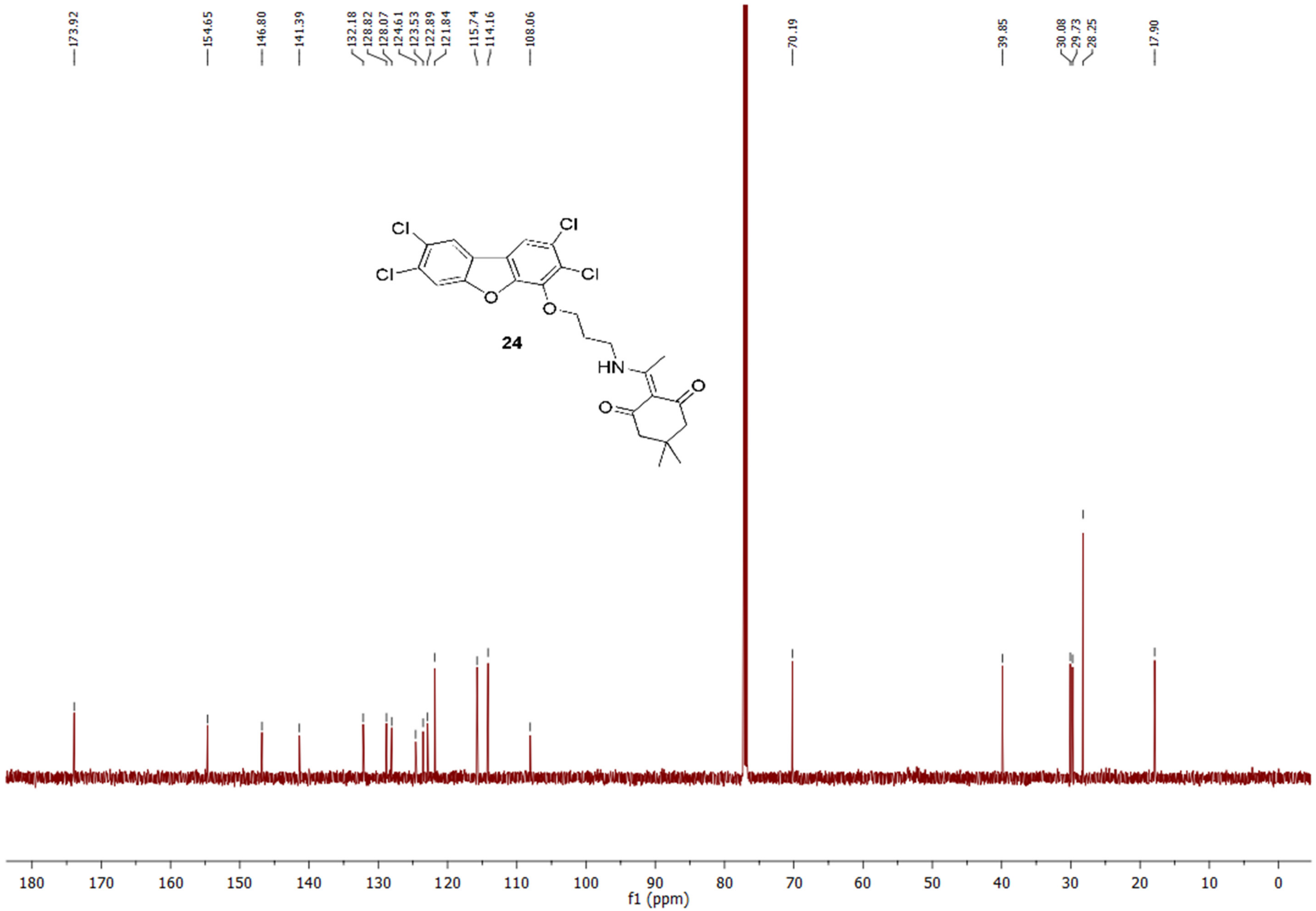

173.92
154.65
146.80
141.39
132.18
128.82
128.07
124.61
123.53
122.89
121.84
115.74
114.16
108.06
70.19
39.85
30.08
29.73
28.25
17.90
Cl
Cl
Cl
Cl
O
O
24
HN
O
O
180 170 160 150 140 130 120 110 100 90 80 70 60 50 40 30 20 10 0
f1 (ppm)

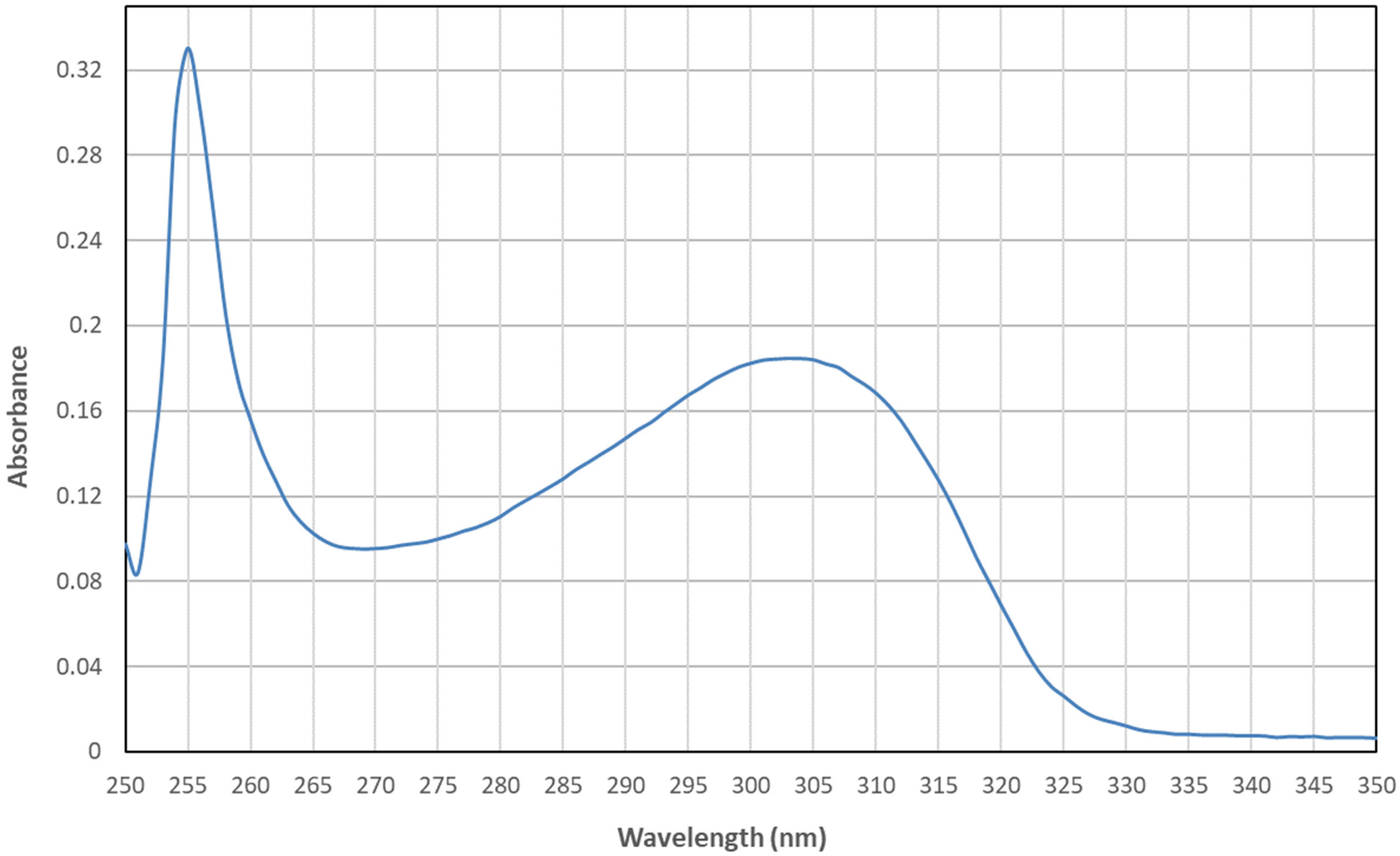

Figure S1. The UV spectrum (250–350 nm) of amino-substituted dioxin **17** in DMF.

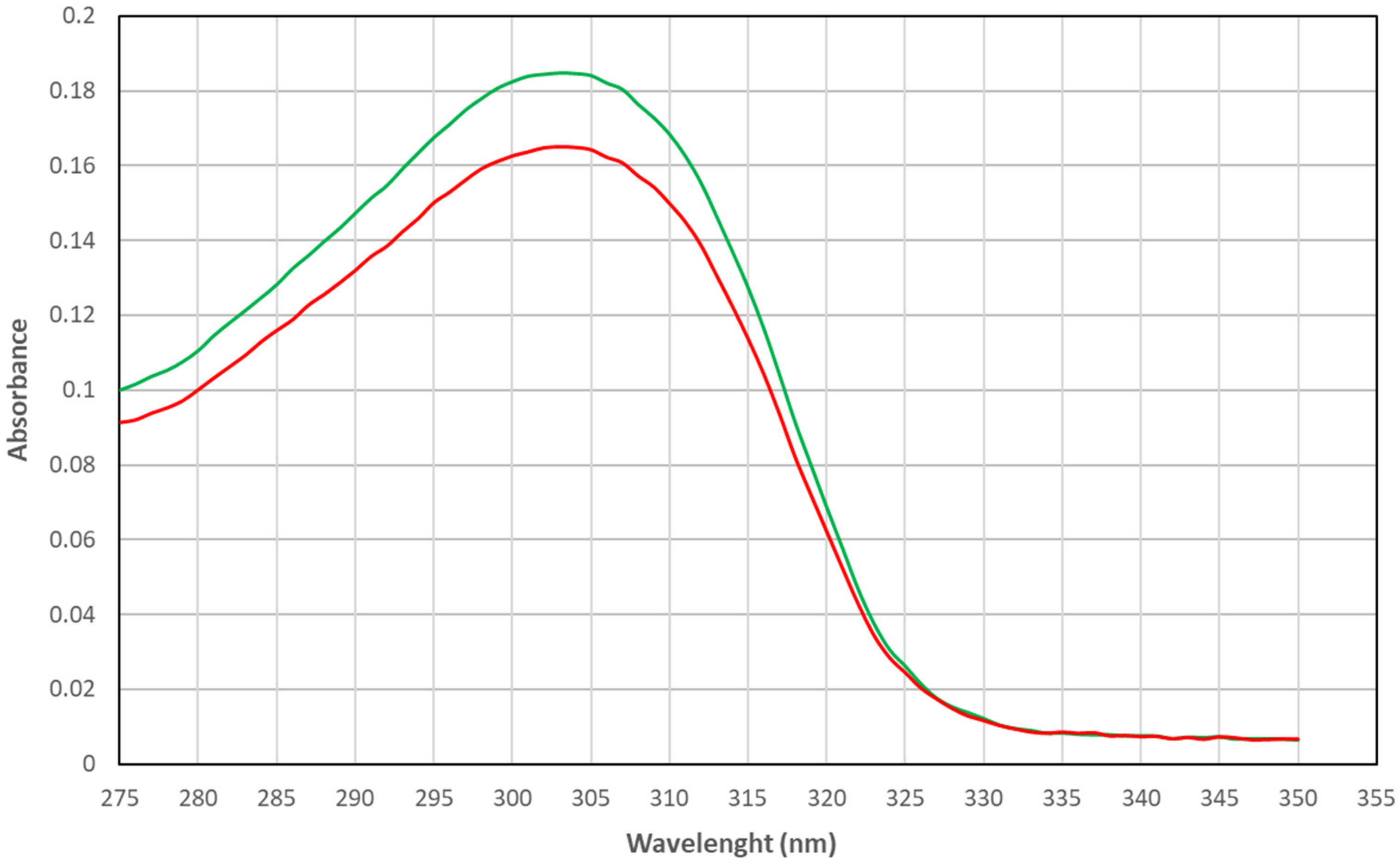

Figure S2. The UV spectrum (275–350 nm), in DMF, of amino-substituted dioxin **17** before (green line) and after (red line) coupling with the carboxy-substituted magnetic beads.

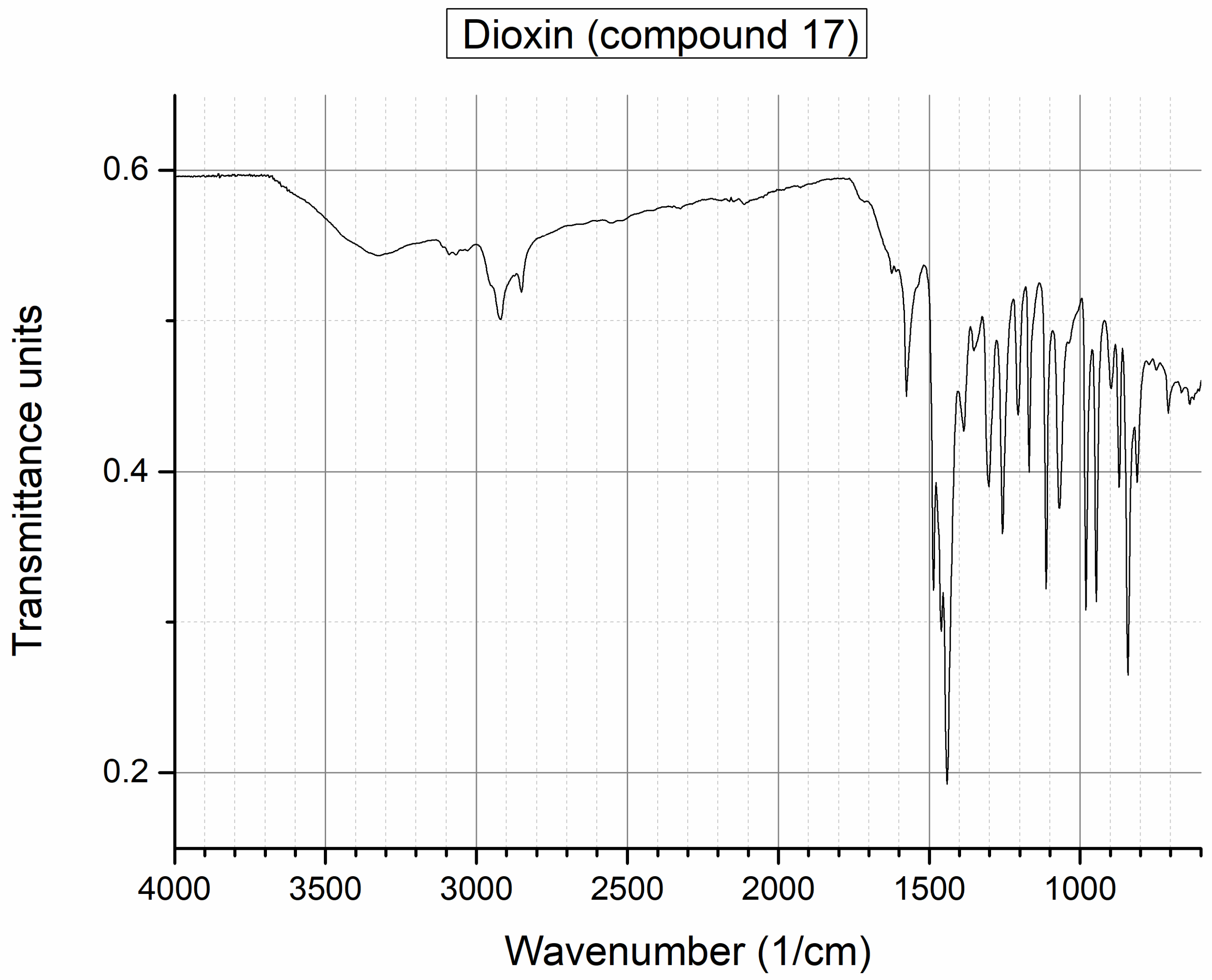

Figure S3. The ATR-FTIR spectrum of amino-substituted dioxin **17**.

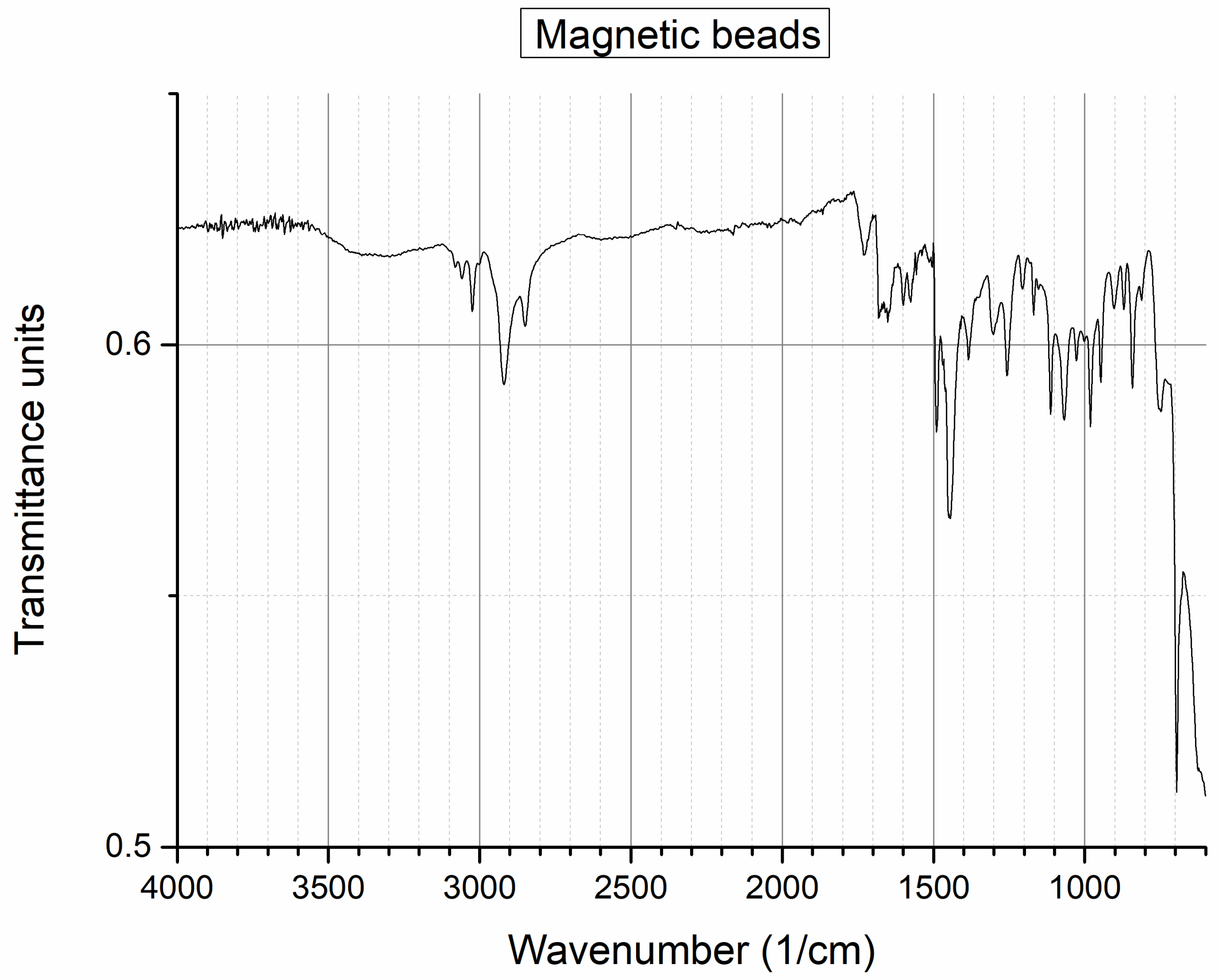

Figure S4. The ATR-FTIR spectrum of magnetic beads.

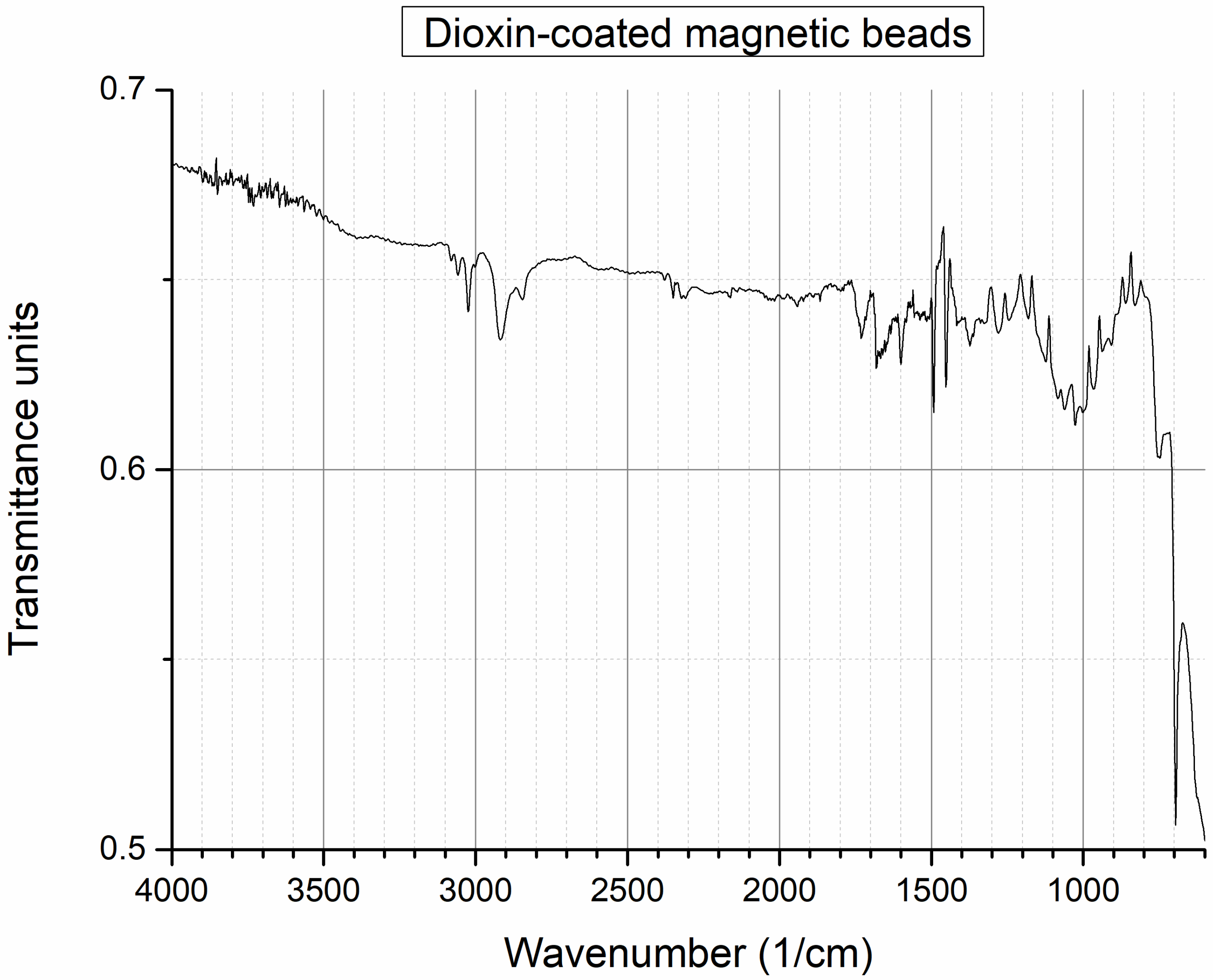

Figure S5. The ATR-FTIR spectrum of magnetic beads coated with the amino-substituted dioxin **17**.

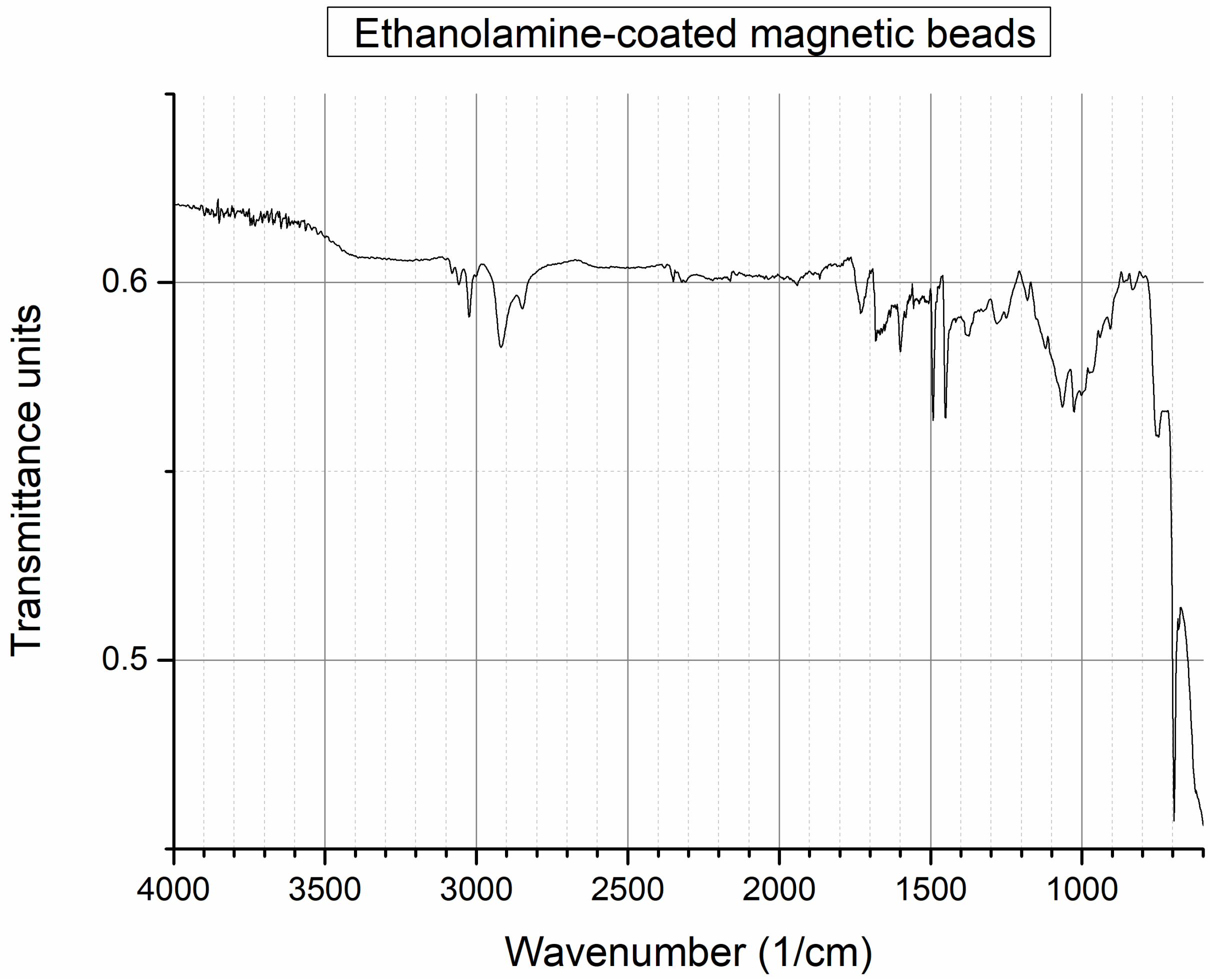

Figure S6. The ATR-FTIR spectrum of magnetic beads coated with ethanolamine.
